# Supplementary material for: Risk factors, symptom reporting, healthcare-seeking behaviour and adherence to public health guidance: protocol for Virus Watch, a prospective community cohort study
Source: BMJ Open. 2021 Jun 23;11(6):e048042. doi: 10.1136/bmjopen-2020-048042 (PMC8230990; doi:10.1136/bmjopen-2020-048042)
Supplement: Supplementary data [file bmjopen-2020-048042supp001.pdf]

Appendix 1 - Study inclusion and exclusion criteria:

Inclusion:

- Households self-select into the study.
- Participants need to join as a household (all must take part).
- They need to have internet connection on a phone, tablet or computer, email and at least one adult that can read English.

Exclusion Criteria

We will exclude participants if:

- Number of householders exceeds 6.
- Those without internet connection on a phone, tablet or computer, or an email address available to them as they will be unable to register
- There is no adult in the household who can read English (from March 2021 this will no longer be an exclusion criteria)
- A household is defined as one or more people (not necessarily related) whose usual residence (4days/week or more) is at the same address. These householders share
- cooking facilities, a living room or sitting room or dining area.
