# Supplementary material for: Risk factors, symptom reporting, healthcare-seeking behaviour and adherence to public health guidance: protocol for Virus Watch, a prospective community cohort study
Source: BMJ Open. 2021 Jun 23;11(6):e048042. doi: 10.1136/bmjopen-2020-048042 (PMC8230990; doi:10.1136/bmjopen-2020-048042)
Supplement: Supplementary data [file bmjopen-2020-048042supp002.pdf]

Confidential

Baseline for Lead Householder

Baseline Survey for [hh1_fname] [hh1_sname]

alive

____

The baseline survey collects some basic information about each person participating in Virus Watch. We will ask about each household member in turn, please ask each adult to complete their own information and help children to complete their sections or complete them for them

[hh1_fname] [hh1_sname]: At birth you were described as?

- ◯ Male
- ◯ Female
- ◯ Intersex
- ◯ Prefer not to say

[hh1_fname] [hh1_sname]: Do you know your NHS Number? This can usually be found on an NHS letter

- ◯ Yes
- ◯ No

[hh1_fname] [hh1_sname]: What is your NHS Number?

____

[hh1_fname] [hh1_sname]: Where is your place of Birth?

- ◯ United Kingdom
- ◯ Other

[hh1_fname] [hh1_sname]: When did you first come to live in the UK (approximately)?
DD/MM/YYYY

____

Hayward A, *et al. BMJ Open* 2021; 11:e048042. doi: 10.1136/bmjopen-2020-048042

[hh1_fname] [hh1_sname]: Please specify where you were born:

- ◯ Afghanistan
- ◯ Albania
- ◯ Algeria
- ◯ American Samoa
- ◯ Andorra
- ◯ Angola
- ◯ Anguilla
- ◯ Antarctica
- ◯ Antigua And Barbuda
- ◯ Argentina
- ◯ Armenia
- ◯ Aruba
- ◯ Australia
- ◯ Austria
- ◯ Azerbaijan
- ◯ Bahamas
- ◯ Bahrain
- ◯ Bangladesh
- ◯ Barbados
- ◯ Belarus
- ◯ Belgium
- ◯ Belize
- ◯ Benin
- ◯ Bermuda
- ◯ Bhutan
- ◯ Bolivia
- ◯ Bosnia And Herzegovina
- ◯ Botswana
- ◯ Bouvet Island
- ◯ Brazil
- ◯ British Indian Ocean Territory
- ◯ Brunei Darussalam
- ◯ Bulgaria
- ◯ Burkina Faso
- ◯ Burundi
- ◯ Cambodia
- ◯ Cameroon
- ◯ Canada
- ◯ Cape Verde
- ◯ Cayman Islands
- ◯ Central African Republic
- ◯ Chad
- ◯ Chile
- ◯ China
- ◯ Christmas Island
- ◯ Cocos (keeling) Islands
- ◯ Colombia
- ◯ Comoros
- ◯ Congo
- ◯ Congo, The Democratic Republic Of The
- ◯ Cook Islands
- ◯ Costa Rica
- ◯ Cote D'ivoire
- ◯ Croatia
- ◯ Cuba
- ◯ Cyprus
- ◯ Czech Republic
- ◯ Denmark
- ◯ Djibouti
- ◯ Dominica
- ◯ Dominican Republic
- ◯ East Timor
- ◯ Ecuador
- ◯ Egypt
- ◯ El Salvador
- ◯ Equatorial Guinea
- ◯ Eritrea
- ◯ Estonia
- ◯ Ethiopia

◯ Falkland Islands (malvinas)
◯ Faroe Islands
◯ Fiji
◯ Finland
◯ France
◯ French Guiana
◯ French Polynesia
◯ French Southern Territories
◯ Gabon
◯ Gambia
◯ Georgia
◯ Germany
◯ Ghana
◯ Gibraltar
◯ Greece
◯ Greenland
◯ Grenada
◯ Guadeloupe
◯ Guam
◯ Guatemala
◯ Guinea
◯ Guinea-bissau
◯ Guyana
◯ Haiti
◯ Heard Island And Mcdonald Islands
◯ Holy See (vatican City State)
◯ Honduras
◯ Hong Kong
◯ Hungary
◯ Iceland
◯ India
◯ Indonesia
◯ Iran, Islamic Republic Of
◯ Iraq
◯ Ireland
◯ Israel
◯ Italy (Italian Republic)
◯ Jamaica
◯ Japan
◯ Jordan
◯ Kazakstan
◯ Kenya
◯ Kiribati
◯ Korea, Democratic People's Republic Of
◯ Korea, Republic Of
◯ Kosovo
◯ Kuwait
◯ Kyrgyzstan
◯ Lao People's Democratic Republic
◯ Latvia
◯ Lebanon
◯ Lesotho
◯ Liberia
◯ Libyan Arab Jamahiriya
◯ Liechtenstein
◯ Lithuania
◯ Luxembourg
◯ Macau
◯ Macedonia, The Former Yugoslav Republic Of
◯ Madagascar
◯ Malawi
◯ Malaysia
◯ Maldives
◯ Mali
◯ Malta
◯ Marshall Islands
◯ Martinique
◯ Mauritania
◯ Mauritius
◯ Mayotte
◯ Mexico

◯ Micronesia, Federated States Of
◯ Moldova, Republic Of
◯ Monaco
◯ Mongolia
◯ Montserrat
◯ Montenegro
◯ Morocco
◯ Mozambique
◯ Myanmar
◯ Namibia
◯ Nauru
◯ Nepal
◯ Netherlands
◯ Netherlands Antilles
◯ New Caledonia
◯ New Zealand
◯ Nicaragua
◯ Niger
◯ Nigeria
◯ Niue
◯ Norfolk Island
◯ Northern Mariana Islands
◯ Norway
◯ Oman
◯ Pakistan
◯ Palau
◯ Palestinian Territory, Occupied
◯ Panama
◯ Papua New Guinea
◯ Paraguay
◯ Peru
◯ Philippines
◯ Pitcairn
◯ Poland
◯ Portugal
◯ Puerto Rico
◯ Qatar
◯ Reunion
◯ Romania
◯ Russian Federation
◯ Rwanda
◯ Saint Helena
◯ Saint Kitts And Nevis
◯ Saint Lucia
◯ Saint Pierre And Miquelon
◯ Saint Vincent And The Grenadines
◯ Samoa
◯ San Marino
◯ Sao Tome And Principe
◯ Saudi Arabia
◯ Senegal
◯ Serbia
◯ Seychelles
◯ Sierra Leone
◯ Singapore
◯ Slovakia
◯ Slovenia
◯ Solomon Islands
◯ Somalia
◯ South Africa
◯ South Georgia And The South Sandwich Islands
◯ Spain
◯ Sri Lanka
◯ Sudan
◯ Suriname
◯ Svalbard And Jan Mayen
◯ Swaziland
◯ Sweden
◯ Switzerland
◯ Syrian Arab Republic
◯ Taiwan, Province Of China

	◯ Tajikistan<br>◯ Tanzania, United Republic Of<br>◯ Thailand<br>◯ Togo<br>◯ Tokelau<br>◯ Tonga<br>◯ Trinidad And Tobago<br>◯ Tunisia<br>◯ Turkey<br>◯ Turkmenistan<br>◯ Turks And Caicos Islands<br>◯ Tuvalu<br>◯ Uganda<br>◯ Ukraine<br>◯ United Arab Emirates<br>◯ United Kingdom<br>◯ United States<br>◯ United States Minor Outlying Islands<br>◯ Uruguay<br>◯ Uzbekistan<br>◯ Vanuatu<br>◯ Venezuela<br>◯ VietNam<br>◯ Virgin Islands, British<br>◯ Virgin Islands, U.s.<br>◯ Wallis And Futuna<br>◯ Western Sahara<br>◯ Yemen<br>◯ Zambia<br>◯ Zimbabwe
[hh1_fname] [hh1_sname]: What is your ethnic group?	◯ White - English/ Welsh/ Scottish/ Northern Irish/ British<br>◯ White - Irish<br>◯ White - Gypsy or Irish Traveller<br>◯ Any other white background (please describe)<br>◯ Asian/ Asian British - Indian<br>◯ Asian/ Asian British - Pakistani<br>◯ Asian/ Asian British - Bangladeshi<br>◯ Asian/ Asian British - Chinese<br>◯ Any other Asian/ Asian British background (please describe)<br>◯ Black African<br>◯ Black Caribbean<br>◯ Any other Black/ African/ Caribbean background (please describe)<br>◯ Arab<br>◯ Any other ethnic group (please describe)<br>◯ Mixed/ multiple ethnic groups - White and Black Caribbean<br>◯ Mixed/ multiple ethnic groups - White and Black African<br>◯ Mixed/ multiple ethnic groups - White and Asian<br>◯ Any other mixed/ multiple ethnic background (please describe)<br>◯ Prefer not to say
[hh1_fname] [hh1_sname]: Please describe your ethnic group:	__________________________________
[hh1_fname] [hh1_sname]: Are you pregnant?	◯ Yes<br>◯ No

BMJ Publishing Group Limited (BMJ) disclaims all liability and responsibility arising from any reliance placed on this supplemental material which has been supplied by the author(s)

Confidential

What trimester of pregnancy are you in?	◯ Less than 12 Weeks<br>◯ 12 Weeks to 24 Weeks<br>◯ More than 24 weeks
Contact details	
[hh1_fname] [hh1_sname]: What is your mobile phone number?<br>This is so we can call you to make blood taking appointments and send your test results if you are selected for the swabbing part of the study.<br>If you do not have a mobile phone, please enter your landline phone number, and we will seek alternative arrangements to send your results if you are selected for the swabbing part of the study.	______________________
Address Line 1:<br>This is to send you swabs (if you are chosen by the study team to partake in the swabbing study)	______________________
Address Line 2:	______________________
Address Line 3:	______________________
Post Code:	______________________

Hayward A, *et al. BMJ Open* 2021; 11:e048042. doi: 10.1136/bmjopen-2020-048042

Medical Background for [hh1_fname] [hh1_sname]

People's health can influence the severity of COVID illness, we want to find out more about this.

[hh1_fname] [hh1_sname]: Please provide your general practitioner's details:
This will allow us to link to your information to hospital records more accurately.

Name of Surgery: ____________________

Address Line 1: ____________________

Address Line 2: ____________________

Address Line 3: ____________________

Post Code: ____________________

[hh1_fname] [hh1_sname] : Has a doctor or other health professional ever told you that you have any of the following conditions?
Please select all that apply.

- ☐ Asthma
- ☐ Arthritis
- ☐ Congestive heart failure
- ☐ Coronary heart disease
- ☐ Angina
- ☐ Heart attack or myocardial infarction
- ☐ Stroke
- ☐ Emphysema
- ☐ Chronic bronchitis
- ☐ COPD (Chronic Obstructive Pulmonary Disease)
- ☐ Cystic fibrosis
- ☐ Hypothyroidism or an under-active thyroid
- ☐ Any kind of liver condition
- ☐ Cancer or malignancy
- ☐ Insulin treated diabetes
- ☐ Other diabetes
- ☐ Epilepsy
- ☐ High blood pressure/hypertension
- ☐ An emotional, nervous or psychiatric problem
- ☐ Multiple Sclerosis
- ☐ HIV
- ☐ Chronic kidney disease
- ☐ Conditions affecting the brain and nerves, such as Parkinson's disease, motor neurone disease, multiple sclerosis (MS), a learning disability or cerebral palsy
- ☐ Problems with your spleen or you've had your spleen removed
- ☐ Sickle cell disease
- ☐ Other long standing/chronic condition
- ☐ None of these

[hh1_fname] [hh1_sname]: Please specify: ____________________

Confidential

[hh1_fname] [hh1_sname]: What type of cancer or malignancy was that?<br>Please select all that apply	☐ Bowel/colorectal<br>☐ Lung<br>☐ Breast<br>☐ Prostate<br>☐ Liver<br>☐ Skin cancer or melanoma<br>☐ Blood or bone marrow cancer, such as leukaemia<br>☐ Other
[hh1_fname] [hh1_sname]: What type of cancer or malignancy was that?<br>Please select all that apply	☐ Bowel/colorectal<br>☐ Lung<br>☐ Breast<br>☐ Liver<br>☐ Skin cancer or melanoma<br>☐ Blood or bone marrow cancer, such as leukaemia<br>☐ Other
[hh1_fname] [hh1_sname]: Has a doctor or other health professional ever told you that you have any of these conditions?<br>Please select all that apply	☐ Asthma<br>☐ Cystic fibrosis<br>☐ Insulin treated diabetes<br>☐ Epilepsy<br>☐ Conditions affecting the brain and nerves, such as Parkinson's disease, motor neurone disease, multiple sclerosis (MS), a learning disability or cerebral palsy<br>☐ Sickle cell disease<br>☐ Other long standing/chronic condition<br>☐ None of these
[hh1_fname] [hh1_sname] : Have you received a letter from the NHS, saying that "the NHS has identified you as someone at risk of severe illness if you catch coronavirus, because you have an underlying disease or health condition that means if you catch the virus, you are more likely to be admitted to hospital than others"?	◯ Yes<br>◯ No
[hh1_fname] [hh1_sname] : Do you know your height and weight?	◯ Yes<br>◯ No
[hh1_fname] [hh1_sname]: Do you know your height in imperial (feet and inches) or metric (centimetres)?<br>Please select the unit you prefer if you know both	◯ Imperial (Feet and Inches)<br>◯ Metric (centimetres)
[hh1_fname] [hh1_sname]: What is your height in centimetres (cm)?<br>Please enter digits only, e.g. '5' and not 'five'	______________________
[hh1_fname] [hh1_sname]: How many feet tall are you (rounded down)?<br>Please enter the feet component of your height. For example if you're 5 foot 4, please enter 5	______________________
[hh1_fname] [hh1_sname]: How many inches tall are you above your feet value?<br>Please enter the inches component of your height. For example if you're 5 foot 4, please enter 4	______________________

Confidential

[hh1_fname] [hh1_sname]: Do you know your weight in imperial (stone and pounds-lbs) or metric(kilograms)? Please select the unit you prefer if you know both	◯ Imperial (stone and pounds)<br>◯ Metric (kilograms)
[hh1_fname] [hh1_sname]: How much do you weigh in kilograms (kg)? Please enter digits only, e.g. '5' and not 'five'	______________________
[hh1_fname] [hh1_sname]: What is your weight in stone, rounded down? For example if you are 8 stone, 10 pounds, please enter 8. If you do not use stone, please feel free to leave this blank and enter your weight fully in pounds	______________________
[hh1_fname] [hh1_sname]: How much do you weight in pounds (lbs) (above your stone weight)? For example, if you are 8 stone, 10 pounds, please enter 10.If you did not enter a value for stone, please enter your weight fully in lbs here	______________________

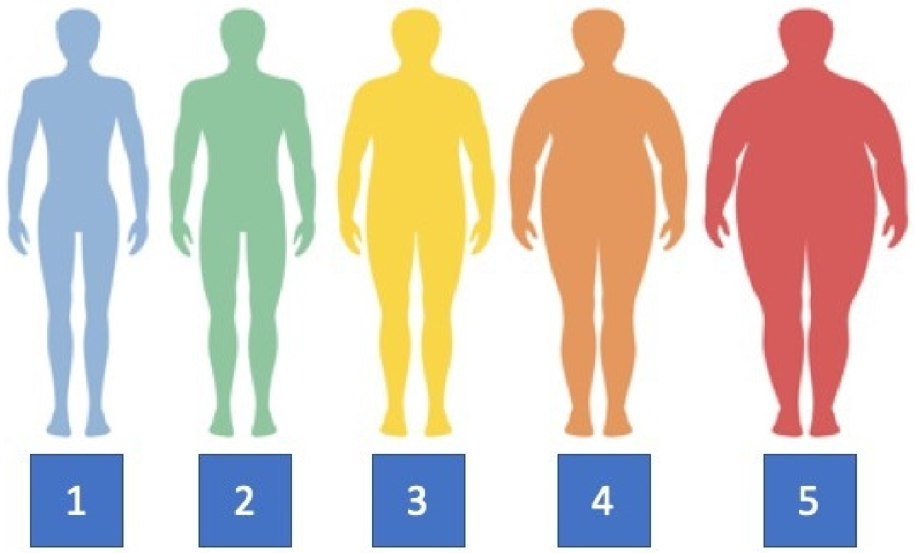

Hayward A, *et al. BMJ Open* 2021; 11:e048042. doi: 10.1136/bmjopen-2020-048042

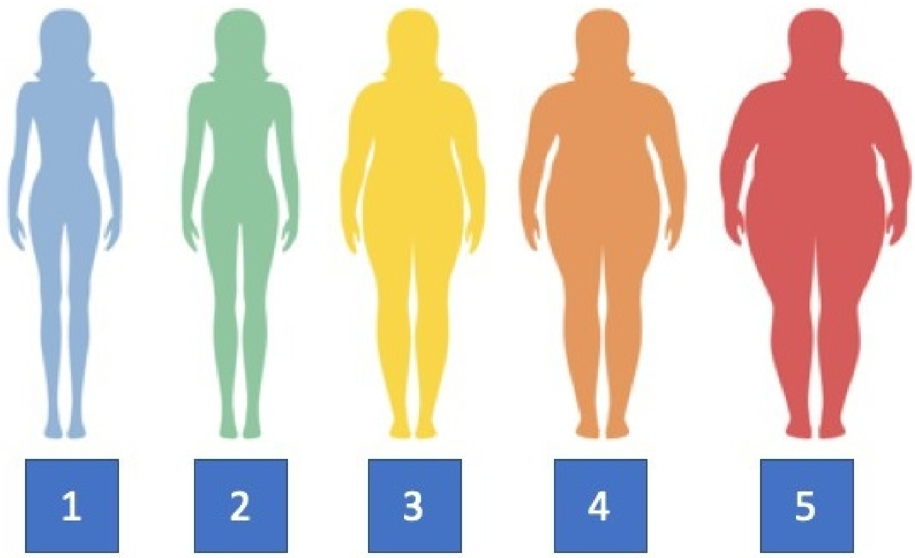

[hh1_fname] [hh1_sname]: Referring to the illustration above, which body shape bests describes your body shape?

◯ 1
◯ 2
◯ 3
◯ 4
◯ 5

HairSex hormones that affect male pattern baldness may also affect COVID severity

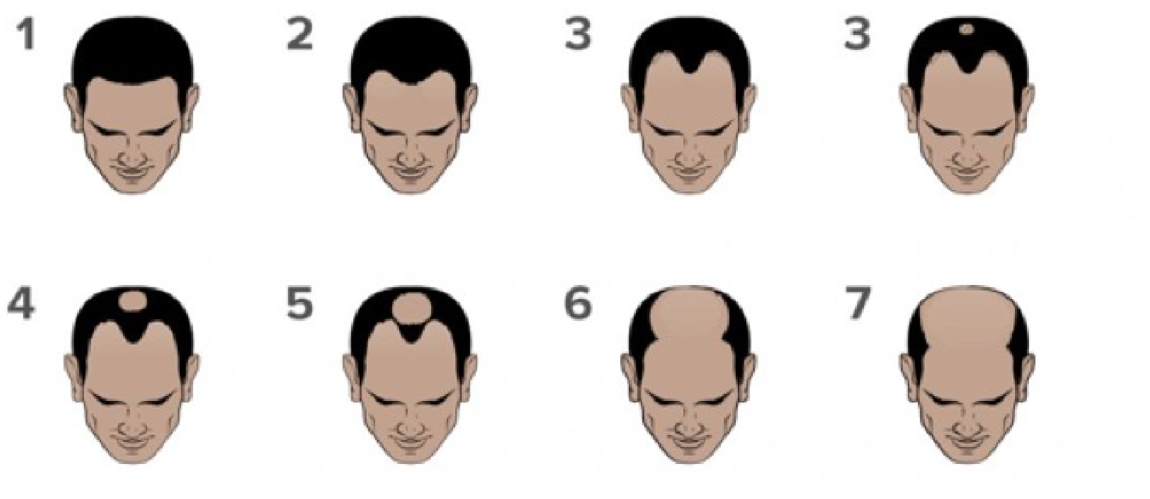

[hh1_fname] [hh1_sname]: Referring to the illustration above, please select a number from 1-7 that best describes your hair	◯ 1<br>◯ 2<br>◯ 3<br>◯ 4<br>◯ 5<br>◯ 6<br>◯ 7

MedicationSome medicines may affect your risk of getting respiratory infections and may either protect against severe diseases or possibly increase the risk. We want to find out more about this

[hh1_fname] [hh1_sname] : Do you take any medication?	◯ Yes<br>◯ No
[hh1_fname] [hh1_sname]: Are you currently receiving treatment or taking medications that may affect your immune system?<br>Please select all that apply	☐ Medication following an organ transplant<br>☐ Medicines such as steroid tablets that weaken the immune system<br>☐ Targeted therapy or chemotherapy for cancer treatment<br>☐ Radiotherapy for cancer treatment<br>☐ Other treatment or medication that may affect immune system<br>☐ None of these
[hh1_fname] [hh1_sname]: Do you regularly take medicine to surpress gastric acid?<br>Please select all that apply	☐ Ranitidine (e.g. Zantac)<br>☐ Omeprazole (e.g. Losec)<br>☐ Antacids (e.g. Rennies)<br>☐ None of these
[hh1_fname] [hh1_sname]: Which of the following medicines do you take?<br>Please select all that apply	☐ Regularly taking Aspirin<br>☐ Regularly taking "NSAIDS" e.g. Ibuprofen, nurofen, diclofenic, naproxen.<br>☐ Regularly taking blood pressure medicines ending in "-pril" such as enalapril, lisinopril, captopril, ramipril<br>☐ Regularly taking blood pressure measurements ending in "-sartan" such as losartan, valsartan, irbesartan<br>☐ Regularly taking anticoagulants e.g warfarin, ivaroxaban (Xarelto), dabigatran (Pradaxa), apixaban (Eliquis), edoxaban (Lixiana)<br>☐ Steroid tablets<br>☐ Regularly use a steroid inhaler<br>☐ Regularly take statins e.g. atorvastatin (Lipitor)<br>☐ None of these
[hh1_fname] [hh1_sname]: Which of the following medicines do you take?<br>Please select all that apply	☐ Steroid tablets<br>☐ Regularly use a steroid inhaler
[hh1_fname] [hh1_sname]: Do you take any vitamin supplements?<br>Please select all that apply	☐ Vitamin C Supplements<br>☐ Vitamin D Supplements<br>☐ Other<br>☐ None
[hh1_fname] [hh1_sname]: Have you ever had a flu vaccine?	◯ Yes<br>◯ No

Hayward A, *et al. BMJ Open* 2021; 11:e048042. doi: 10.1136/bmjopen-2020-048042

[hh1_fname] [hh1_sname]: Approximately when did you have your most recent flu vaccine?<br>If you do not remember the exact date, please select an approximate date	______________________
Drinking and Smoking<br>Drinking and smoking affects the risk of many diseases. We want to find out if it affects the risk of COVID-19 infection	
[hh1_fname] [hh1_sname]: Have you ever smoked cigarettes regularly?	◯ Yes<br>◯ No
[hh1_fname] [hh1_sname]: And do you smoke cigarettes at all nowadays?	◯ Yes<br>◯ No
[hh1_fname] [hh1_sname]: How many cigarettes do you smoke daily?<br>Please enter digits only, e.g. '5' and not 'five'	______________________
[hh1_fname] [hh1_sname]: When did you give up smoking?	◯ Less than 3 months ago<br>◯ 3 - 6 months ago<br>◯ More than 6 months ago but less than 1 year ago<br>◯ 1 year or more ago
[hh1_fname] [hh1_sname]: Thinking about the past month, how often did you have a drink containing alcohol?	◯ Daily<br>◯ 4-6 Times per week<br>◯ 2-3 Times per week<br>◯ Weekly or Less<br>◯ 2-4 times per month<br>◯ Never
[hh1_fname] [hh1_sname]: How many drinks do you have on a typical day when you are drinking?	◯ 1-2 Drinks<br>◯ 3-4 Drinks<br>◯ 5-6 Drinks<br>◯ 7-9 Drinks<br>◯ 10+
Accessing Health care during the lockdown	
[hh1_fname] [hh1_sname]: Have you had any healthcare appointments cancelled, postponed or changed to a telephone or online (including video) consultation since the start of the pandemic?	◯ Yes<br>◯ No

Hayward A, *et al. BMJ Open* 2021; 11:e048042. doi: 10.1136/bmjopen-2020-048042

Please tick all that applied	☐ I had an operation cancelled or postponed<br>☐ I had a planned hospital admission cancelled or postponed<br>☐ I had a hospital outpatient appointment cancelled or postponed<br>☐ I had a hospital outpatient appointment changed to a phone call or online (including video) consultation<br>☐ I had a GP appointment cancelled or postponed<br>☐ I had a GP appointment changed to a phone call or online (including video) consultation<br>☐ I had another NHS appointment cancelled or postponed<br>☐ I had another NHS appointment changed to a phone call or online (including video)

Hayward A, *et al. BMJ Open* 2021; 11:e048042. doi: 10.1136/bmjopen-2020-048042

Confidential

Employment status for [hh1_fname] [hh1_sname]

Many people's work has been affected by the coronavirus and people's work can affect their chance of catching coronavirus. We'd like to know about your work and how it has been effected by the coronavirus.

[hh1_fname] [hh1_sname]: Thinking back to earlier this year, before the outbreak of the coronavirus pandemic. Which of these description best describes your work status?	◯ Employed full time<br>◯ Employed part time<br>◯ Self employed full time<br>◯ Self employed part time<br>◯ Retired<br>◯ Student<br>◯ Looking after house/family (not looking for work)<br>◯ Permanently sick or disabled<br>◯ Unemployed<br>◯ None of the above
[hh1_fname] [hh1_sname]: What is/was the name of your job?	______________________
[hh1_fname] [hh1_sname]: Please describe what you do/did at work	______________________
[hh1_fname] [hh1_sname]: Are you a health or social care worker?	◯ Yes<br>◯ No
What setting do you work in?	◯ Secondary Care<br>◯ Accident and emergency<br>◯ Primary Care<br>◯ Care home (residential or nursing)<br>◯ Community<br>◯ Other (specify)
Other (Please Specify)	______________________
[hh1_fname] [hh1_sname]: Please select your healthcare profession	◯ Doctor<br>◯ Nurse<br>◯ Profession allied to medicine (e.g. occupational therapy, physiotherapy, podiatry)<br>◯ Psychological Professions<br>◯ Pharmacy<br>◯ Midwifery<br>◯ Healthcare science (e.g. laboratory, radiology)<br>◯ Management<br>◯ Porter<br>◯ Cleaner<br>◯ Administrative Staff with regular patient contact<br>◯ Administrative Staff with minimal or no patient contact<br>◯ Care Worker<br>◯ Personal Assistant<br>◯ Social Worker<br>◯ Community support and outreach worker<br>◯ Other
[hh1_fname] [hh1_sname]: Please specify	______________________

[hh1_fname] [hh1_sname]: What was your last job when you were working? __________________________________

[hh1_fname] [hh1_sname]: Are you a healthcare student? e.g. medical student, student nurse
- ◯ Yes
- ◯ No

Employment during the pandemic

[hh1_fname] [hh1_sname]: BEFORE THE PANDEMIC, how many hours of paid work did you work per week?
Please enter digits only, e.g. '5' and not 'five' __________________________________

[hh1_fname] [hh1_sname]: SINCE THE PANDEMIC, during the months of March, April, May, how many hours of paid work have you worked since each week?
Please enter digits only, e.g. '5' and not 'five' __________________________________

It looks like you have reduced your paid work during the lockdown.

[hh1_fname] [hh1_sname] : Why have you had to reduce the number of hours?
- ◯ Laid off by employer with certain recall date
- ◯ Laid off or made redundant by employer with some prospect of recall
- ◯ Employer cut hours
- ◯ Have been put on furlough or paid leave
- ◯ Using annual leave
- ◯ On paid or statutory sick leave
- ◯ On unpaid sick leave
- ◯ Caring for children or others
- ◯ Other reasons

[hh1_fname] [hh1_sname] : Why have you had to reduce the number of hours?
- ◯ My business has been directly affected by regulations on opening or other new regulations
- ◯ My business has been directly affected by limited supplies that I need for my business
- ◯ My business has been directly affected by reduced demand for my services or products
- ◯ Illness
- ◯ Self-isolating
- ◯ Caring for children or others
- ◯ Other reasons

[hh1_fname] [hh1_sname]: Have you received a written letter from your employer informing you that you have been furloughed under the Coronavirus Job Retention scheme?
- ◯ Yes
- ◯ No

Working from home during the pandemic

[hh1_fname] [hh1_sname]: How often did you WORK FROM HOME during the following three time periods:

	(Nearly) Always	Often	Sometimes	(Almost) Never
Before the lockdown (before March 2020)	◯	◯	◯	◯

Confidential

During the main lockdown (Mid March to Mid May) when we were asked to stay at Home, Save Lives, Support the NHS	○	○	○	○
After the easing of restrictions in mid-May, when we were encouraged to go back to work if we could not work from home (Stay Alert, Control the Virus, Save Lives)	○	○	○	○

[hh1_fname] [hh1_sname]: How do/did you usually travel to work?
Please choose one or more to represent a typical day

	Before the lockdown (before March 2020)	During the main lockdown (Mid March to Mid May) when we were asked to stay at Home, Save Lives, Support the NHS	After the easing of restrictions in mid-May, when we were encouraged to go back to work if we could not work from home (Stay Alert, Control the Virus, Save Lives)
By car or van	☐	☐	☐
Motorcycle, moped or scooter	☐	☐	☐
Taxi or minicab	☐	☐	☐
Train	☐	☐	☐
Underground	☐	☐	☐
Bus	☐	☐	☐
Tram or light railway	☐	☐	☐
Cycle	☐	☐	☐
Walk	☐	☐	☐
Other	☐	☐	☐
I did not travel to work during this period	☐	☐	☐

[hh1_fname] [hh1_sname]: BEFORE LOCKDOWN, how long did it take to get to work each day?
Please provide the time for a one-way journey in minutes or enter 0 if you did not travel to work in this period

____

[hh1_fname] [hh1_sname]: DURING LOCKDOWN, how long did it take to get to work each day?
Please provide the time for a one-way journey in minutes or enter 0 if you did not travel to work in this period

____

BMJ Publishing Group Limited (BMJ) disclaims all liability and responsibility arising from any reliance placed on this supplemental material which has been supplied by the author(s)

Confidential

[hh1_fname] [hh1_sname]: AFTER THE EASING OF RESTRICTIONS, how long did it take to get to work each day?
Please provide the time for a one-way journey in minutes or enter 0 if you did not travel to work in this period ________________________________

Hayward A, *et al. BMJ Open* 2021; 11:e048042. doi: 10.1136/bmjopen-2020-048042

Finances details for [hh1_fname] [hh1_sname]

Many people have been affected financially by the coronavirus. We'd like to know how you have been affected and how you and your household are coping.

[hh1_fname] [hh1_sname]: What is your combined household income last year?<br>We want to understand how COVID-19 impacts households with different levels of income	◯ 0-9,999<br>◯ 10,000- 24,999<br>◯ 25,000 - 49,999<br>◯ 50,000 - 74,999<br>◯ 75,000 - 99,999<br>◯ 100,000 - 124,999<br>◯ 125,000 - 149,999<br>◯ 150,000 - 174,999<br>◯ 175,000 - 199,999<br>◯ 200,000 or more<br>◯ Prefer not to say
[hh1_fname] [hh1_sname] : BEFORE THE PANDEMIC, how would you say you were managing financially? Would you say you were:	◯ Living comfortably<br>◯ Doing alright<br>◯ Just about getting by<br>◯ Finding it quite difficult<br>◯ Finding it very difficult
[hh1_fname] [hh1_sname]: How would you say you are managing financially now? Would you say you were:	◯ Living comfortably<br>◯ Doing alright<br>◯ Just about getting by<br>◯ Finding it quite difficult<br>◯ Finding it very difficult
[hh1_fname] [hh1_sname]: BEFORE THE PANDEMIC, have you ever needed to use a food bank?	◯ Never<br>◯ Less than once a week<br>◯ Once a week or more
[hh1_fname] [hh1_sname]: SINCE THE PANDEMIC, have you needed to use a food bank?	◯ Never<br>◯ Less than once a week<br>◯ Once a week or more

Confidential

Caring information during the lockdown - [hh1_fname] [hh1_sname]

[hh1_fname] [hh1_sname]: About how many hours a week did you spend on childcare or home-schooling during the lockdown?
Please enter digits only, e.g. '5' and not 'five'

____

[hh1_fname] [hh1_sname]: Who is mainly responsible for looking after the children or home schooling?

- ◯ Mainly you
- ◯ Mainly your husband/wife/partner
- ◯ Jointly with your husband/wife/partner
- ◯ Someone else

Hayward A, *et al. BMJ Open* 2021; 11:e048042. doi: 10.1136/bmjopen-2020-048042

Previous COVID-19 like illness for [hh1_fname] [hh1_sname]

[hh1_fname] [hh1_sname]: Have you EVER come into contact with anyone that was known or presumed to have COVID-19?
Close contact includes:
Physical contact with another personA five minute conversation with someone less than 2 metres awayBeing less than 2 metres away from someone for 15 minutes or more, even if you didn't talk to each otherPlease select all that apply.

- ☐ No
- ☐ Yes (a household member)
- ☐ Yes (at work)
- ☐ Yes (a non-household friend or relation)
- ☐ Yes (in public)
- ☐ Yes (other)
- ☐ Don't know

Did this person have COVID confirmed by a laboratory test?

- ◯ Yes
- ◯ No
- ◯ Don't know

[hh1_fname] [hh1_sname]: Have you had an illness involving Cough, or Fever, or Loss of sense of smell since the 1st of January 2020?

- ◯ Yes
- ◯ No

[hh1_fname] [hh1_sname]: Which month did the illness start and which symptoms did you have?
If you had more than one illness, you may select multiple start months

	Jan 2020	Feb 2020	Mar 2020	Apr 2020	May 2020	Jun 2020	Jul 2020	Aug 2020	Sep 2020	Oct 2020	Nov 2020	Dec 2020	Jan 2021	Feb 2021
Cough	☐	☐	☐	☐	☐	☐	☐	☐	☐	☐	☐	☐	☐	☐
Fever	☐	☐	☐	☐	☐	☐	☐	☐	☐	☐	☐	☐	☐	☐
Loss of sense of smell	☐	☐	☐	☐	☐	☐	☐	☐	☐	☐	☐	☐	☐	☐

[hh1_fname] [hh1_sname]: COVID-19 can cause a wide range of symptoms. Have you had an illness that you think might have been COVID-19, or which was confirmed as COVID-19?

- ◯ Yes
- ◯ No

Please describe in your own words how did the illness started and what you did when you started to feel ill?

____

Please describe in your own words, what symptoms developed and what did you do?

____

Please describe in your own words, how long did your symptoms last and are any symptoms still there?

____

[hh1_fname] [hh1_sname]: Have you ever had a nose or throat swab test for COVID-19?

- ◯ Yes
- ◯ No
- ◯ Not Sure

What month was the swab taken and what was the result?
Please select all the months in which you took each test (if you took multiple) and their results

Confidential

	The test was positive	The test was negative	The test was unclear	I haven't had the result
Jan 2020	◯	◯	◯	◯
Feb 2020	◯	◯	◯	◯
Mar 2020	◯	◯	◯	◯
Apr 2020	◯	◯	◯	◯
May 2020	◯	◯	◯	◯
Jun 2020	◯	◯	◯	◯
Jul 2020	◯	◯	◯	◯
Aug 2020	◯	◯	◯	◯
Sep 2020	◯	◯	◯	◯
Oct 2020	◯	◯	◯	◯
Nov 2020	◯	◯	◯	◯
Dec 2020	◯	◯	◯	◯
Jan 2021	◯	◯	◯	◯
Feb 2021	◯	◯	◯	◯

Hayward A, *et al. BMJ Open* 2021; 11:e048042. doi: 10.1136/bmjopen-2020-048042

Confidential

Thank You

We want to know what questions you think are important so we can try to answer them with the help of tens of thousands of Virus Watch participants. Each month we will ask our participants to say what questions they would like to see answered.

[hh1_fname] [hh1_sname]: What questions would you like Virus Watch to answer? ______________________________

As we come out of a very difficult and tragic period we want to know about your three main worries related to COVID-19 and the COVID-19 response.

[hh1_fname] [hh1_sname]: What are your three main worries about the COVID-19 pandemic?

1st most worrying aspect: ______________________

2nd most worrying aspect: ______________________

3rd most worrying aspect: ______________________

Please click submit to continue
