# Supplementary material for: Risk factors, symptom reporting, healthcare-seeking behaviour and adherence to public health guidance: protocol for Virus Watch, a prospective community cohort study
Source: BMJ Open. 2021 Jun 23;11(6):e048042. doi: 10.1136/bmjopen-2020-048042 (PMC8230990; doi:10.1136/bmjopen-2020-048042)
Supplement: Supplementary data [file bmjopen-2020-048042supp003.pdf]

Confidential

Weekly Survey

Welcome to the weekly followup survey

surveydate ______________________

In Virus Watch we are interested in the following types of symptoms:
General symptoms (e.g. fevers, general muscle aches, headache, joint pain,extreme tiredness, trouble with daily activities around the house)Respiratory Symptoms (e.g. cough, shortness of breath, earache, sore throat, runny nose, blocked nose, sneezing, wheeze, loss or altered senses of smell or taste).Eyes (e.g. eye redness, eye pain,sticky eye, deterioration of eyesight)RashesDigestive symptoms (e.g. diarrhoea or loose stools, vomiting, nausea, abdominal pain)This survey is about symptoms in the week (Monday to Sunday) before you received the email with the survey link.

Download Symptom Diary

[Attachment: "Virus_Watch_Symptom_Diary.pdf"]

Have you or anyone in the household had any of these symptoms in the past week?

Please continue to report weekly symptoms even if you believe them to be related to a recent vaccine you have had.

○ Yes
○ No

Did any household members receive a COVID-19 test result in the past week?

○ Yes
○ No

Has anyone in the household been advised to self-isolate in the past week?

○ Yes
○ No

Please indicate who received a result for a COVID-19 (swab or lateral flow) test in the past week: If you have had multiple tests this week please report any positive test dates OR your last negative test dates (if all were negative)

[go_arm_1][hh1_fname] [go_arm_1][hh1_sname] ☐

[go_arm_1][hh1_fname] [go_arm_1][hh1_sname]: What was the result of the COVID-19 test?

○ Positive
○ Negative
○ Unclear

[go_arm_1][hh1_fname] [go_arm_1][hh1_sname]: When was the test taken? ______________________

[go_arm_1][hh1_fname] [go_arm_1][hh1_sname]: When did you receive the test result? ______________________

[go_arm_1][hh2_fname] [go_arm_1][hh2_sname] ☐

[go_arm_1][hh2_fname] [go_arm_1][hh2_sname]: What was the result of the COVID-19 test?

○ Positive
○ Negative
○ Unclear

[go_arm_1][hh2_fname] [go_arm_1][hh2_sname]: When was the test taken? ______________________

Hayward A, *et al. BMJ Open* 2021; 11:e048042. doi: 10.1136/bmjopen-2020-048042

Confidential

[go_arm_1][hh2_fname] [go_arm_1][hh2_sname]: When did you receive the test result?	______________________
[go_arm_1][hh3_fname] [go_arm_1][hh3_sname]	☐
[go_arm_1][hh3_fname] [go_arm_1][hh3_sname]: What was the result of the COVID-19 test?	◯ Positive<br>◯ Negative<br>◯ Unclear
[go_arm_1][hh3_fname] [go_arm_1][hh3_sname]: When was the test taken?	______________________
[go_arm_1][hh3_fname] [go_arm_1][hh3_sname]: When did you receive the test result?	______________________
[go_arm_1][hh4_fname] [go_arm_1][hh4_sname]	☐
[go_arm_1][hh4_fname] [go_arm_1][hh4_sname]: What was the result of the COVID-19 test?	◯ Positive<br>◯ Negative<br>◯ Unclear
[go_arm_1][hh4_fname] [go_arm_1][hh4_sname]: When was the test taken?	______________________
[go_arm_1][hh4_fname] [go_arm_1][hh4_sname]: When did you receive the test result?	______________________
[go_arm_1][hh5_fname] [go_arm_1][hh5_sname]	☐
[go_arm_1][hh5_fname] [go_arm_1][hh5_sname]: What was the result of the COVID-19 test?	◯ Positive<br>◯ Negative<br>◯ Unclear
[go_arm_1][hh5_fname] [go_arm_1][hh5_sname]: When was the test taken?	______________________
[go_arm_1][hh5_fname] [go_arm_1][hh5_sname]: When did you receive the test result?	______________________
[go_arm_1][hh6_fname] [go_arm_1][hh6_sname]	☐
[go_arm_1][hh6_fname] [go_arm_1][hh6_sname]: What was the result of the COVID-19 test?	◯ Positive<br>◯ Negative<br>◯ Unclear
[go_arm_1][hh6_fname] [go_arm_1][hh6_sname]: When was the test taken?	______________________
[go_arm_1][hh6_fname] [go_arm_1][hh6_sname]: When did you receive the test result?	______________________
Please indicate who was advised to self isolate in the past week:	
[go_arm_1][hh1_fname] [go_arm_1][hh1_sname]	☐

Hayward A, *et al. BMJ Open* 2021; 11:e048042. doi: 10.1136/bmjopen-2020-048042

Confidential

[go_arm_1][hh1_fname] [go_arm_1][hh1_sname]: Which of the following led to the request to self-isolate?	◯ The Test and Trace programme advised me to self-isolate because I have symptoms of COVID-19<br>◯ The Test and Trace programme advised me to self-isolate because I am had a positive test result<br>◯ The Test and Trace programme advised me to self isolate because I was in contact with a COVID-19 case<br>◯ The NHS COVID-19 app alerted me that I had been in contact with a COVID-19 case<br>◯ I have returned from a country where quarantine is advised after return.<br>◯ Other reason
[go_arm_1][hh2_fname] [go_arm_1][hh2_sname]	☐
[go_arm_1][hh2_fname] [go_arm_1][hh2_sname]: Which of the following led to the request to self-isolate?	◯ The Test and Trace programme advised me to self-isolate because I have symptoms of COVID-19<br>◯ The Test and Trace programme advised me to self-isolate because I am had a positive test result<br>◯ The Test and Trace programme advised me to self isolate because I was in contact with a COVID-19 case<br>◯ The NHS COVID-19 app alerted me that I had been in contact with a COVID-19 case<br>◯ I have returned from a country where quarantine is advised after return.<br>◯ Other reason
[go_arm_1][hh3_fname] [go_arm_1][hh3_sname]	☐
[go_arm_1][hh3_fname] [go_arm_1][hh3_sname]: Which of the following led to the request to self-isolate?	◯ The Test and Trace programme advised me to self-isolate because I have symptoms of COVID-19<br>◯ The Test and Trace programme advised me to self-isolate because I am had a positive test result<br>◯ The Test and Trace programme advised me to self isolate because I was in contact with a COVID-19 case<br>◯ The NHS COVID-19 app alerted me that I had been in contact with a COVID-19 case<br>◯ I have returned from a country where quarantine is advised after return.<br>◯ Other reason
[go_arm_1][hh4_fname] [go_arm_1][hh4_sname]	☐

Hayward A, *et al. BMJ Open* 2021; 11:e048042. doi: 10.1136/bmjopen-2020-048042

[go_arm_1][hh4_fname] [go_arm_1][hh4_sname]: Which of the following led to the request to self-isolate?	◯ The Test and Trace programme advised me to self-isolate because I have symptoms of COVID-19<br>◯ The Test and Trace programme advised me to self-isolate because I am had a positive test result<br>◯ The Test and Trace programme advised me to self isolate because I was in contact with a COVID-19 case<br>◯ The NHS COVID-19 app alerted me that I had been in contact with a COVID-19 case<br>◯ I have returned from a country where quarantine is advised after return.<br>◯ Other reason
[go_arm_1][hh5_fname] [go_arm_1][hh5_sname]	☐
[go_arm_1][hh5_fname] [go_arm_1][hh5_sname]: Which of the following led to the request to self-isolate?	◯ The Test and Trace programme advised me to self-isolate because I have symptoms of COVID-19<br>◯ The Test and Trace programme advised me to self-isolate because I am had a positive test result<br>◯ The Test and Trace programme advised me to self isolate because I was in contact with a COVID-19 case<br>◯ The NHS COVID-19 app alerted me that I had been in contact with a COVID-19 case<br>◯ I have returned from a country where quarantine is advised after return.<br>◯ Other reason
[go_arm_1][hh6_fname] [go_arm_1][hh6_sname]	☐
[go_arm_1][hh6_fname] [go_arm_1][hh6_sname]: Which of the following led to the request to self-isolate?	◯ The Test and Trace programme advised me to self-isolate because I have symptoms of COVID-19<br>◯ The Test and Trace programme advised me to self-isolate because I am had a positive test result<br>◯ The Test and Trace programme advised me to self isolate because I was in contact with a COVID-19 case<br>◯ The NHS COVID-19 app alerted me that I had been in contact with a COVID-19 case<br>◯ I have returned from a country where quarantine is advised after return.<br>◯ Other reason
Please indicate who has had symptoms in the past week:	
[go_arm_1][hh1_fname] [go_arm_1][hh1_sname]:	◯ Yes, symptoms that have been present for less than 2 months<br>◯ Yes, symptoms that developed after a COVID-19 like illness and have lasted for more than 2 months<br>◯ Yes, symptoms that are part of a long term chronic illness<br>◯ No symptoms

Confidential

[go_arm_1][hh2_fname] [go_arm_1][hh2_sname]:	◯ Yes, symptoms that have been present for less than 2 months<br>◯ Yes, symptoms that developed after a COVID-19 like illness and have lasted for more than 2 months<br>◯ Yes, symptoms that are part of a long term chronic illness<br>◯ No symptoms
[go_arm_1][hh3_fname] [go_arm_1][hh3_sname]:	◯ Yes, symptoms that have been present for less than 2 months<br>◯ Yes, symptoms that developed after a COVID-19 like illness and have lasted for more than 2 months<br>◯ Yes, symptoms that are part of a long term chronic illness<br>◯ No symptoms
[go_arm_1][hh4_fname] [go_arm_1][hh4_sname]:	◯ Yes, symptoms that have been present for less than 2 months<br>◯ Yes, symptoms that developed after a COVID-19 like illness and have lasted for more than 2 months<br>◯ Yes, symptoms that are part of a long term chronic illness<br>◯ No symptoms
[go_arm_1][hh5_fname] [go_arm_1][hh5_sname]:	◯ Yes, symptoms that have been present for less than 2 months<br>◯ Yes, symptoms that developed after a COVID-19 like illness and have lasted for more than 2 months<br>◯ Yes, symptoms that are part of a long term chronic illness<br>◯ No symptoms
[go_arm_1][hh6_fname] [go_arm_1][hh6_sname]:	◯ Yes, symptoms that have been present for less than 2 months<br>◯ Yes, symptoms that developed after a COVID-19 like illness and have lasted for more than 2 months<br>◯ Yes, symptoms that are part of a long term chronic illness<br>◯ No symptoms

Please indicate whether the following members have received a result for a COVID-19 (swab or lateral flow) test in the past week: If you have had multiple tests this week please report any positive test dates OR your last negative test dates (if all were negative)

[go_arm_1][hh1_fname] [go_arm_1][hh1_sname]:	☐
[go_arm_1][hh1_fname] [go_arm_1][hh1_sname]: What was the result of this test?	◯ Positive<br>◯ Negative<br>◯ Unclear
[go_arm_1][hh1_fname] [go_arm_1][hh1_sname]: When was the test taken?	______________________________
[go_arm_1][hh1_fname] [go_arm_1][hh1_sname]: When did you receive the test result?	______________________________
[go_arm_1][hh2_fname] [go_arm_1][hh2_sname]:	☐

[go_arm_1][hh2_fname] [go_arm_1][hh2_sname]: What was the result of this test?	◯ Positive<br>◯ Negative<br>◯ Unclear
[go_arm_1][hh2_fname] [go_arm_1][hh2_sname]: When was the test taken?	______________________
[go_arm_1][hh2_fname] [go_arm_1][hh2_sname]: When did you receive the test result?	______________________
[go_arm_1][hh3_fname] [go_arm_1][hh3_sname]:	☐
[go_arm_1][hh3_fname] [go_arm_1][hh3_sname]: What was the result of this test?	◯ Positive<br>◯ Negative<br>◯ Unclear
[go_arm_1][hh3_fname] [go_arm_1][hh3_sname]: When was the test taken?	______________________
[go_arm_1][hh3_fname] [go_arm_1][hh3_sname]: When did you receive the test result?	______________________
[go_arm_1][hh4_fname] [go_arm_1][hh4_sname]:	☐
[go_arm_1][hh4_fname] [go_arm_1][hh4_sname]: What was the result of this test?	◯ Positive<br>◯ Negative<br>◯ Unclear
[go_arm_1][hh4_fname] [go_arm_1][hh4_sname]: When was the test taken?	______________________
[go_arm_1][hh4_fname] [go_arm_1][hh4_sname]: When did you receive the test result?	______________________
[go_arm_1][hh5_fname] [go_arm_1][hh5_sname]:	☐
[go_arm_1][hh5_fname] [go_arm_1][hh5_sname]: What was the result of this test?	◯ Positive<br>◯ Negative<br>◯ Unclear
[go_arm_1][hh5_fname] [go_arm_1][hh5_sname]: When was the test taken?	______________________
[go_arm_1][hh5_fname] [go_arm_1][hh5_sname]: When did you receive the test result?	______________________
[go_arm_1][hh6_fname] [go_arm_1][hh6_sname]:	☐
[go_arm_1][hh6_fname] [go_arm_1][hh6_sname]: What was the result of this test?	◯ Positive<br>◯ Negative<br>◯ Unclear
[go_arm_1][hh6_fname] [go_arm_1][hh6_sname]: When was the test taken?	______________________

Confidential

[go_arm_1][hh6_fname] [go_arm_1][hh6_sname]: When did you receive the test result?	______________________________
Please indiciate whether the following household members been advised to self-isolate in the past week:	
[go_arm_1][hh1_fname] [go_arm_1][hh1_sname]	☐
[go_arm_1][hh1_fname] [go_arm_1][hh1_sname]: Which of the following led to the request to self-isolate?	◯ The Test and Trace programme advised me to self-isolate because I have symptoms of COVID-19<br>◯ The Test and Trace programme advised me to self-isolate because I am had a positive test result<br>◯ The Test and Trace programme advised me to self isolate because I was in contact with a COVID-19 case<br>◯ The NHS COVID-19 app alerted me that I had been in contact with a COVID-19 case<br>◯ I have returned from a country where quarantine is advised after return.<br>◯ Other reason
[go_arm_1][hh2_fname] [go_arm_1][hh2_sname]	☐
[go_arm_1][hh2_fname] [go_arm_1][hh2_sname]: Which of the following led to the request to self-isolate?	◯ The Test and Trace programme advised me to self-isolate because I have symptoms of COVID-19<br>◯ The Test and Trace programme advised me to self-isolate because I am had a positive test result<br>◯ The Test and Trace programme advised me to self isolate because I was in contact with a COVID-19 case<br>◯ The NHS COVID-19 app alerted me that I had been in contact with a COVID-19 case<br>◯ I have returned from a country where quarantine is advised after return.<br>◯ Other reason
[go_arm_1][hh3_fname] [go_arm_1][hh3_sname]	☐
[go_arm_1][hh3_fname] [go_arm_1][hh3_sname]: Which of the following led to the request to self-isolate?	◯ The Test and Trace programme advised me to self-isolate because I have symptoms of COVID-19<br>◯ The Test and Trace programme advised me to self-isolate because I am had a positive test result<br>◯ The Test and Trace programme advised me to self isolate because I was in contact with a COVID-19 case<br>◯ The NHS COVID-19 app alerted me that I had been in contact with a COVID-19 case<br>◯ I have returned from a country where quarantine is advised after return.<br>◯ Other reason
[go_arm_1][hh4_fname] [go_arm_1][hh4_sname]	☐

Confidential

[go_arm_1][hh4_fname] [go_arm_1][hh4_sname]: Which of the following led to the request to self-isolate?	◯ The Test and Trace programme advised me to self-isolate because I have symptoms of COVID-19<br>◯ The Test and Trace programme advised me to self-isolate because I am had a positive test result<br>◯ The Test and Trace programme advised me to self isolate because I was in contact with a COVID-19 case<br>◯ The NHS COVID-19 app alerted me that I had been in contact with a COVID-19 case<br>◯ I have returned from a country where quarantine is advised after return.<br>◯ Other reason
[go_arm_1][hh5_fname] [go_arm_1][hh5_sname]	☐
[go_arm_1][hh5_fname] [go_arm_1][hh5_sname]: Which of the following led to the request to self-isolate?	◯ The Test and Trace programme advised me to self-isolate because I have symptoms of COVID-19<br>◯ The Test and Trace programme advised me to self-isolate because I am had a positive test result<br>◯ The Test and Trace programme advised me to self isolate because I was in contact with a COVID-19 case<br>◯ The NHS COVID-19 app alerted me that I had been in contact with a COVID-19 case<br>◯ I have returned from a country where quarantine is advised after return.<br>◯ Other reason
[go_arm_1][hh6_fname] [go_arm_1][hh6_sname]	☐
[go_arm_1][hh6_fname] [go_arm_1][hh6_sname]: Which of the following led to the request to self-isolate?	◯ The Test and Trace programme advised me to self-isolate because I have symptoms of COVID-19<br>◯ The Test and Trace programme advised me to self-isolate because I am had a positive test result<br>◯ The Test and Trace programme advised me to self isolate because I was in contact with a COVID-19 case<br>◯ The NHS COVID-19 app alerted me that I had been in contact with a COVID-19 case<br>◯ I have returned from a country where quarantine is advised after return.<br>◯ Other reason

Hayward A, *et al. BMJ Open* 2021; 11:e048042. doi: 10.1136/bmjopen-2020-048042

Confidential

Vaccination

Has anyone in the household received a COVID-19 vaccine in the past week?	◯ Yes<br>◯ No<br>◯ Unsure (e.g. as part of a blinded COVID-19 trial)
Please indicate who received a COVID-19 vaccine:	
[go_arm_1][hh1_fname] [go_arm_1][hh1_sname]	☐
[go_arm_1][hh1_fname] [go_arm_1][hh1_sname]: Please select which dose(s) of the COVID-19 vaccine you received?	☐ 1st Dose<br>☐ 2nd Dose
[go_arm_1][hh1_fname] [go_arm_1][hh1_sname]: Which type of vaccine did you receive as the 1st dose?	◯ Pfizer Biontech vaccine<br>◯ Oxford AstraZeneca vaccine<br>◯ Other vaccine<br>◯ Don't know/Don't remember
[go_arm_1][hh1_fname] [go_arm_1][hh1_sname]: What date did you receive the 1st dose?<br>(dd-mm-yyyy)<br><br>Please provide an estimate if you cannot recall the date	__________________________
[go_arm_1][hh1_fname] [go_arm_1][hh1_sname]: Which type of vaccine did you receive as the 2nd dose?	◯ Pfizer Biontech vaccine<br>◯ Oxford AstraZeneca vaccine<br>◯ Other vaccine<br>◯ Don't know/Don't remember
[go_arm_1][hh1_fname] [go_arm_1][hh1_sname]: What date did you receive the 2nd dose?<br>(dd-mm-yyyy)<br><br>Please provide an estimate if you cannot recall the date	__________________________
[go_arm_1][hh2_fname] [go_arm_1][hh2_sname]	☐
[go_arm_1][hh2_fname] [go_arm_1][hh2_sname]: Please select which dose(s) of the COVID-19 vaccine you received?	☐ 1st Dose<br>☐ 2nd Dose
[go_arm_1][hh2_fname] [go_arm_1][hh2_sname]: Which type of vaccine did you receive as the 1st dose?	◯ Pfizer Biontech vaccine<br>◯ Oxford AstraZeneca vaccine<br>◯ Other vaccine<br>◯ Don't know/Don't remember
[go_arm_1][hh2_fname] [go_arm_1][hh2_sname]: What date did you receive the 1st dose? (dd-mm-yyyy)<br><br>Please provide an estimate if you cannot recall the date	__________________________

Hayward A, *et al. BMJ Open* 2021; 11:e048042. doi: 10.1136/bmjopen-2020-048042

Confidential

[go_arm_1][hh2_fname] [go_arm_1][hh2_sname]: Which type of vaccine did you receive as the 2nd dose?	◯ Pfizer Biontech vaccine<br>◯ Oxford AstraZeneca vaccine<br>◯ Other vaccine<br>◯ Don't know/Don't remember
[go_arm_1][hh2_fname] [go_arm_1][hh2_sname]: What date did you receive the 2nd dose? (dd-mm-yyyy)<br><br>Please provide an estimate if you cannot recall the date	________________________________
[go_arm_1][hh3_fname] [go_arm_1][hh3_sname]	☐
[go_arm_1][hh3_fname] [go_arm_1][hh3_sname]: Please select which dose(s) of the COVID-19 vaccine you received?	☐ 1st Dose<br>☐ 2nd Dose
[go_arm_1][hh3_fname] [go_arm_1][hh3_sname]: Which type of vaccine did you receive as the 1st dose?	◯ Pfizer Biontech vaccine<br>◯ Oxford AstraZeneca vaccine<br>◯ Other vaccine<br>◯ Don't know/Don't remember
[go_arm_1][hh3_fname] [go_arm_1][hh3_sname]: What date did you receive the 1st dose? (dd-mm-yyyy)<br><br>Please provide an estimate if you cannot recall the date	________________________________
[go_arm_1][hh3_fname] [go_arm_1][hh3_sname]: Which type of vaccine did you receive as the 2nd dose?	◯ Pfizer Biontech vaccine<br>◯ Oxford AstraZeneca vaccine<br>◯ Other vaccine<br>◯ Don't know/Don't remember
[go_arm_1][hh3_fname] [go_arm_1][hh3_sname]: What date did you receive the 2nd dose? (dd-mm-yyyy)<br><br>Please provide an estimate if you cannot recall the date	________________________________
[go_arm_1][hh4_fname] [go_arm_1][hh4_sname]	☐
[go_arm_1][hh4_fname] [go_arm_1][hh4_sname]: Please select which dose(s) of the COVID-19 vaccine you received?	☐ 1st Dose<br>☐ 2nd Dose
[go_arm_1][hh4_fname] [go_arm_1][hh4_sname]: Which type of vaccine did you receive as the 1st dose?	◯ Pfizer Biontech vaccine<br>◯ Oxford AstraZeneca vaccine<br>◯ Other vaccine<br>◯ Don't know/Don't remember
[go_arm_1][hh4_fname] [go_arm_1][hh4_sname]: What date did you receive the 1st dose? (dd-mm-yyyy)<br><br>Please provide an estimate if you cannot recall the date	________________________________

Hayward A, *et al. BMJ Open* 2021; 11:e048042. doi: 10.1136/bmjopen-2020-048042

Confidential

[go_arm_1][hh4_fname] [go_arm_1][hh4_sname]: Which type of vaccine did you receive as the 2nd dose?	◯ Pfizer Biontech vaccine<br>◯ Oxford AstraZeneca vaccine<br>◯ Other vaccine<br>◯ Don't know/Don't remember
[go_arm_1][hh4_fname] [go_arm_1][hh4_sname]: What date did you receive the 2nd dose? (dd-mm-yyyy)<br><br>Please provide an estimate if you cannot recall the date	______________________
[go_arm_1][hh5_fname] [go_arm_1][hh5_sname]	☐
[go_arm_1][hh5_fname] [go_arm_1][hh5_sname]: Please select which dose(s) of the COVID-19 vaccine you received?	☐ 1st Dose<br>☐ 2nd Dose
[go_arm_1][hh5_fname] [go_arm_1][hh5_sname]: Which type of vaccine did you receive as the 1st dose?	◯ Pfizer Biontech vaccine<br>◯ Oxford AstraZeneca vaccine<br>◯ Other vaccine<br>◯ Don't know/Don't remember
[go_arm_1][hh5_fname] [go_arm_1][hh5_sname]: What date did you receive the 1st dose? (dd-mm-yyyy)<br><br>Please provide an estimate if you cannot recall the date	______________________
[go_arm_1][hh5_fname] [go_arm_1][hh5_sname]: Which type of vaccine did you receive as the 2nd dose?	◯ Pfizer Biontech vaccine<br>◯ Oxford AstraZeneca vaccine<br>◯ Other vaccine<br>◯ Don't know/Don't remember
[go_arm_1][hh5_fname] [go_arm_1][hh5_sname]: What date did you receive the 2nd dose? (dd-mm-yyyy)<br><br>Please provide an estimate if you cannot recall the date	______________________
[go_arm_1][hh6_fname] [go_arm_1][hh6_sname]	☐
[go_arm_1][hh6_fname] [go_arm_1][hh6_sname]: Please select which dose(s) of the COVID-19 vaccine you received?	☐ 1st Dose<br>☐ 2nd Dose
[go_arm_1][hh6_fname] [go_arm_1][hh6_sname]: Which type of vaccine did you receive as the 1st dose?	◯ Pfizer Biontech vaccine<br>◯ Oxford AstraZeneca vaccine<br>◯ Other vaccine<br>◯ Don't know/Don't remember
[go_arm_1][hh6_fname] [go_arm_1][hh6_sname]: What date did you receive the 1st dose? (dd-mm-yyyy)<br><br>Please provide an estimate if you cannot recall the date	______________________

[go_arm_1][hh6_fname] [go_arm_1][hh6_sname]: Which type of vaccine did you receive as the 2nd dose?	◯ Pfizer Biontech vaccine<br>◯ Oxford AstraZeneca vaccine<br>◯ Other vaccine<br>◯ Don't know/Don't remember
[go_arm_1][hh6_fname] [go_arm_1][hh6_sname]: What date did you receive the 2nd dose? (dd-mm-yyyy)<br><br>Please provide an estimate if you cannot recall the date	________________________________

Thank you for letting us know that someone in your household has been ill. We hope they feel better soon. Please always follow NHS and Public Health advice when someone is ill. We will always have a link to the latest COVID-19 advice on the Virus Watch Website.

If anyone has new symptoms to report, the following survey will ask about any illness and related health care as well as asking about isolation, time off work, measures to help stop infections spreading and activities that household members have done in the last week.

The survey usually takes about 10 minutes to complete for each member of the household who has been ill.

Thank you for completing the survey - we will be in touch again next week.

Hayward A, *et al. BMJ Open* 2021; 11:e048042. doi: 10.1136/bmjopen-2020-048042
