# Supplementary material for: Risk factors, symptom reporting, healthcare-seeking behaviour and adherence to public health guidance: protocol for Virus Watch, a prospective community cohort study
Source: BMJ Open. 2021 Jun 23;11(6):e048042. doi: 10.1136/bmjopen-2020-048042 (PMC8230990; doi:10.1136/bmjopen-2020-048042)
Supplement: Supplementary data [file bmjopen-2020-048042supp004.pdf]

Confidential

Wkhh1

Symptoms - [go_arm_1][hh1_fname] [go_arm_1][hh1_sname]

The following sections are about the symptoms, use of treatments and access of medical services in the past week (the Monday-Sunday before you received the email with this survey link).

[go_arm_1][hh1_fname]: What parts of the body did your symptoms affect?<br>Select all that apply	☐ General symptoms (fevers, general muscle aches, headache, joint pain,extreme tiredness, trouble with daily activities around the house)<br>☐ Respiratory Symptoms (e.g. cough, shortness of breath, earache, sore throat, runny nose, blocked nose, sneezing, wheeze, loss or altered senses of smell or taste)<br>☐ Eyes (e.g. eye redness, eye pain, sticky eye, deterioration of eyesight)<br>☐ Rash<br>☐ Digestive symptoms (e.g. diarrhoea or loose stools, vomiting, nausea, abdominal pain)
[go_arm_1][hh1_fname] [go_arm_1][hh1_sname]: Which days did you have symptoms?<br>Please check all days that you had any of the above symptoms.	☐ Monday<br>☐ Tuesday<br>☐ Wednesday<br>☐ Thursday<br>☐ Friday<br>☐ Saturday<br>☐ Sunday

Hayward A, *et al. BMJ Open* 2021; 11:e048042. doi: 10.1136/bmjopen-2020-048042

General symptoms - [go_arm_1][hh1_fname] [go_arm_1][hh1_sname]

[go_arm_1][hh1_fname] [go_arm_1][hh1_sname]: Which of these general symptoms did you have?
Select all that apply

- ☐ Fever
- ☐ Feeling Feverish
- ☐ Chills and shakes
- ☐ Night Sweats
- ☐ Muscle Aches
- ☐ Bone or Joint Aches
- ☐ Loss of Appetite
- ☐ Headache
- ☐ Lack of concentration
- ☐ Lighheaded or dizzy
- ☐ Not sleeping
- ☐ Fatigue / Feeling unusually tired
- ☐ Difficulty with daily activities around the house
- ☐ Needed extra time in bed
- ☐ Could not get out of bed
- ☐ Confusion, disorientation, or hallucinations (altered mental state)
- ☐ None of these symptoms

Please identify how severe your symptoms were

Fever

	Less than 37.8 C (100.0 F)	37.8-38.9 C (100-102 F)	39-39.9 C (102-103.9 F)	40 C (104 F) or more	Did not take temperature Don't remember
Monday	◯	◯	◯	◯	◯
Tuesday	◯	◯	◯	◯	◯
Wednesday	◯	◯	◯	◯	◯
Thursday	◯	◯	◯	◯	◯
Friday	◯	◯	◯	◯	◯
Saturday	◯	◯	◯	◯	◯
Sunday	◯	◯	◯	◯	◯

Feeling Feverish

	None	Mild	Moderate	Severe
Monday	◯	◯	◯	◯
Tuesday	◯	◯	◯	◯
Wednesday	◯	◯	◯	◯
Thursday	◯	◯	◯	◯
Friday	◯	◯	◯	◯
Saturday	◯	◯	◯	◯
Sunday	◯	◯	◯	◯

Chills and Shakes

Confidential

	None	Mild	Moderate	Severe
Monday	○	○	○	○
Tuesday	○	○	○	○
Wednesday	○	○	○	○
Thursday	○	○	○	○
Friday	○	○	○	○
Saturday	○	○	○	○
Sunday	○	○	○	○

Night Sweats

	None	Mild	Moderate	Severe
Monday	○	○	○	○
Tuesday	○	○	○	○
Wednesday	○	○	○	○
Thursday	○	○	○	○
Friday	○	○	○	○
Saturday	○	○	○	○
Sunday	○	○	○	○

Muscle Ache

	None	Mild	Moderate	Severe
Monday	○	○	○	○
Tuesday	○	○	○	○
Wednesday	○	○	○	○
Thursday	○	○	○	○
Friday	○	○	○	○
Saturday	○	○	○	○
Sunday	○	○	○	○

Bone or Joint ache

	None	Mild	Moderate	Severe
Monday	○	○	○	○
Tuesday	○	○	○	○
Wednesday	○	○	○	○
Thursday	○	○	○	○
Friday	○	○	○	○
Saturday	○	○	○	○
Sunday	○	○	○	○

Loss of Appetite

Hayward A, *et al. BMJ Open* 2021; 11:e048042. doi: 10.1136/bmjopen-2020-048042

Confidential

	None	Mild	Moderate	Severe
Monday	○	○	○	○
Tuesday	○	○	○	○
Wednesday	○	○	○	○
Thursday	○	○	○	○
Friday	○	○	○	○
Saturday	○	○	○	○
Sunday	○	○	○	○

Headache

	None	Mild	Moderate	Severe
Monday	○	○	○	○
Tuesday	○	○	○	○
Wednesday	○	○	○	○
Thursday	○	○	○	○
Friday	○	○	○	○
Saturday	○	○	○	○
Sunday	○	○	○	○

Confusion, disorientation, or hallucinations (altered mental state)

	None	Mild	Moderate	Severe
Monday	○	○	○	○
Tuesday	○	○	○	○
Wednesday	○	○	○	○
Thursday	○	○	○	○
Friday	○	○	○	○
Saturday	○	○	○	○
Sunday	○	○	○	○

Lack of Concentration

	None	Mild	Moderate	Severe
Monday	○	○	○	○
Tuesday	○	○	○	○
Wednesday	○	○	○	○
Thursday	○	○	○	○
Friday	○	○	○	○
Saturday	○	○	○	○
Sunday	○	○	○	○

Lightheaded or Dizzy

Hayward A, *et al. BMJ Open* 2021; 11:e048042. doi: 10.1136/bmjopen-2020-048042

Confidential

	None	Mild	Moderate	Severe
Monday	○	○	○	○
Tuesday	○	○	○	○
Wednesday	○	○	○	○
Thursday	○	○	○	○
Friday	○	○	○	○
Saturday	○	○	○	○
Sunday	○	○	○	○

Not Sleeping

	None	Mild	Moderate	Severe
Monday	○	○	○	○
Tuesday	○	○	○	○
Wednesday	○	○	○	○
Thursday	○	○	○	○
Friday	○	○	○	○
Saturday	○	○	○	○
Sunday	○	○	○	○

Fatigue/Feeling Unusually Tired

	None	Mild	Moderate	Severe
Monday	○	○	○	○
Tuesday	○	○	○	○
Wednesday	○	○	○	○
Thursday	○	○	○	○
Friday	○	○	○	○
Saturday	○	○	○	○
Sunday	○	○	○	○

Difficulties with Daily Activities

	None	Mild	Moderate	Severe
Monday	○	○	○	○
Tuesday	○	○	○	○
Wednesday	○	○	○	○
Thursday	○	○	○	○
Friday	○	○	○	○
Saturday	○	○	○	○
Sunday	○	○	○	○

Needed extra time in bed

Hayward A, *et al. BMJ Open* 2021; 11:e048042. doi: 10.1136/bmjopen-2020-048042

Confidential

	None	Mild	Moderate	Severe
Monday	○	○	○	○
Tuesday	○	○	○	○
Wednesday	○	○	○	○
Thursday	○	○	○	○
Friday	○	○	○	○
Saturday	○	○	○	○
Sunday	○	○	○	○

Could not get out of bed

	None	Mild	Moderate	Severe
Monday	○	○	○	○
Tuesday	○	○	○	○
Wednesday	○	○	○	○
Thursday	○	○	○	○
Friday	○	○	○	○
Saturday	○	○	○	○
Sunday	○	○	○	○

Hayward A, *et al. BMJ Open* 2021; 11:e048042. doi: 10.1136/bmjopen-2020-048042

Respiratory Symptoms - [go_arm_1][hh1_fname] [go_arm_1][hh1_sname]

[go_arm_1][hh1_fname] [go_arm_1][hh1_sname]: Which Respiratory symptoms did you have?
Select all that apply

- ☐ Runny Nose
- ☐ Blocked Nose
- ☐ Sinus pain / congestion
- ☐ Dry cough
- ☐ Coughing up Green Phlegm
- ☐ Coughing up White Phlegm
- ☐ Loss or change to sense of smell
- ☐ Loss or change to sense of taste
- ☐ Sneezing
- ☐ Sore Throat
- ☐ Swollen tonsils
- ☐ Swollen glands (enlarged lymph nodes)
- ☐ Ear pain or change in hearing
- ☐ Fluid leaking from ear
- ☐ Shortness of breath difficulty breathing
- ☐ Wheezing
- ☐ Chest pain (not changed by breathing or moving)
- ☐ Chest pain when breathing in
- ☐ None of these symptoms

Please identify how severe your symptoms were

Runny Nose

	None	Mild	Moderate	Severe
Monday	○	○	○	○
Tuesday	○	○	○	○
Wednesday	○	○	○	○
Thursday	○	○	○	○
Friday	○	○	○	○
Saturday	○	○	○	○
Sunday	○	○	○	○

Blocked Nose

	None	Mild	Moderate	Severe
Monday	○	○	○	○
Tuesday	○	○	○	○
Wednesday	○	○	○	○
Thursday	○	○	○	○
Friday	○	○	○	○
Saturday	○	○	○	○
Sunday	○	○	○	○

Sinus Pain/Congestion

	None	Mild	Moderate	Severe
Monday	○	○	○	○

Hayward A, *et al. BMJ Open* 2021; 11:e048042. doi: 10.1136/bmjopen-2020-048042

Tuesday	○	○	○	○
Wednesday	○	○	○	○
Thursday	○	○	○	○
Friday	○	○	○	○
Saturday	○	○	○	○
Sunday	○	○	○	○

Dry cough

	None	Mild	Moderate	Severe
Monday	○	○	○	○
Tuesday	○	○	○	○
Wednesday	○	○	○	○
Thursday	○	○	○	○
Friday	○	○	○	○
Saturday	○	○	○	○
Sunday	○	○	○	○

Coughing up Green Phlegm

	None	Mild	Moderate	Severe
Monday	○	○	○	○
Tuesday	○	○	○	○
Wednesday	○	○	○	○
Thursday	○	○	○	○
Friday	○	○	○	○
Saturday	○	○	○	○
Sunday	○	○	○	○

Coughing up White Phlegm

	None	Mild	Moderate	Severe
Monday	○	○	○	○
Tuesday	○	○	○	○
Wednesday	○	○	○	○
Thursday	○	○	○	○
Friday	○	○	○	○
Saturday	○	○	○	○
Sunday	○	○	○	○

Loss or change to sense of smell

	None	Mild	Moderate	Severe
Monday	○	○	○	○
Tuesday	○	○	○	○
Wednesday	○	○	○	○
Thursday	○	○	○	○
Friday	○	○	○	○
Saturday	○	○	○	○
Sunday	○	○	○	○

Loss or change to sense of taste

	None	Mild	Moderate	Severe
Monday	○	○	○	○
Tuesday	○	○	○	○
Wednesday	○	○	○	○
Thursday	○	○	○	○
Friday	○	○	○	○
Saturday	○	○	○	○
Sunday	○	○	○	○

Sneezing

	None	Mild	Moderate	Severe
Monday	○	○	○	○
Tuesday	○	○	○	○
Wednesday	○	○	○	○
Thursday	○	○	○	○
Friday	○	○	○	○
Saturday	○	○	○	○
Sunday	○	○	○	○

Sore Throat

	None	Mild	Moderate	Severe
Monday	○	○	○	○
Tuesday	○	○	○	○
Wednesday	○	○	○	○
Thursday	○	○	○	○
Friday	○	○	○	○
Saturday	○	○	○	○
Sunday	○	○	○	○

Swollen Tonsils

Hayward A, *et al. BMJ Open* 2021; 11:e048042. doi: 10.1136/bmjopen-2020-048042

	None	Mild	Moderate	Severe
Monday	○	○	○	○
Tuesday	○	○	○	○
Wednesday	○	○	○	○
Thursday	○	○	○	○
Friday	○	○	○	○
Saturday	○	○	○	○
Sunday	○	○	○	○

Swollen Glands (enlarged lymph nodes)

	None	Mild	Moderate	Severe
Monday	○	○	○	○
Tuesday	○	○	○	○
Wednesday	○	○	○	○
Thursday	○	○	○	○
Friday	○	○	○	○
Saturday	○	○	○	○
Sunday	○	○	○	○

Ear pain or change in hearing

	None	Mild	Moderate	Severe
Monday	○	○	○	○
Tuesday	○	○	○	○
Wednesday	○	○	○	○
Thursday	○	○	○	○
Friday	○	○	○	○
Saturday	○	○	○	○
Sunday	○	○	○	○

Fluid leaking from ear

	None	Mild	Moderate	Severe
Monday	○	○	○	○
Tuesday	○	○	○	○
Wednesday	○	○	○	○
Thursday	○	○	○	○
Friday	○	○	○	○
Saturday	○	○	○	○
Sunday	○	○	○	○

Shortness of Breath/difficulty breathing

Confidential

	None	Mild	Moderate	Severe
Monday	○	○	○	○
Tuesday	○	○	○	○
Wednesday	○	○	○	○
Thursday	○	○	○	○
Friday	○	○	○	○
Saturday	○	○	○	○
Sunday	○	○	○	○

Wheezing

	None	Mild	Moderate	Severe
Monday	○	○	○	○
Tuesday	○	○	○	○
Wednesday	○	○	○	○
Thursday	○	○	○	○
Friday	○	○	○	○
Saturday	○	○	○	○
Sunday	○	○	○	○

Chest pain (not changed by breathing or moving)

	None	Mild	Moderate	Severe
Monday	○	○	○	○
Tuesday	○	○	○	○
Wednesday	○	○	○	○
Thursday	○	○	○	○
Friday	○	○	○	○
Saturday	○	○	○	○
Sunday	○	○	○	○

Chest pain when breathing in

	None	Mild	Moderate	Severe
Monday	○	○	○	○
Tuesday	○	○	○	○
Wednesday	○	○	○	○
Thursday	○	○	○	○
Friday	○	○	○	○
Saturday	○	○	○	○
Sunday	○	○	○	○

Hayward A, *et al. BMJ Open* 2021; 11:e048042. doi: 10.1136/bmjopen-2020-048042

Other Symptoms - [go_arm_1][hh1_fname] [go_arm_1][hh1_sname]

[go_arm_1][hh1_fname] [go_arm_1][hh1_sname]: Which eye related symptoms did you have this week?
Select all that apply

- ☐ Eye redness
- ☐ Sticky Eye
- ☐ Eye pain
- ☐ Deterioration of eyesight
- ☐ None of these symptoms

[go_arm_1][hh1_fname] [go_arm_1][hh1_sname]: Where did the rash affect you?

- ☐ Rash (all over)
- ☐ Rash (local)
- ☐ None of these symptoms

Where on the body did the rash affect you?

____

[go_arm_1][hh1_fname] [go_arm_1][hh1_sname]: Which gastrointestinal symptoms did you have?
Select all that apply

- ☐ Diarrhoea (even mild)
- ☐ Vomiting (being sick)
- ☐ Nausea (feeling sick)
- ☐ Abdominal pain (not including menstrual pain)
- ☐ None of these symptoms

Please identify how severe your symptoms were

Red Eye(s)

	None	Mild	Moderate	Severe
Monday	○	○	○	○
Tuesday	○	○	○	○
Wednesday	○	○	○	○
Thursday	○	○	○	○
Friday	○	○	○	○
Saturday	○	○	○	○
Sunday	○	○	○	○

Sticky Eye(s)

	None	Mild	Moderate	Severe
Monday	○	○	○	○
Tuesday	○	○	○	○
Wednesday	○	○	○	○
Thursday	○	○	○	○
Friday	○	○	○	○
Saturday	○	○	○	○
Sunday	○	○	○	○

Eye Pain

Hayward A, *et al. BMJ Open* 2021; 11:e048042. doi: 10.1136/bmjopen-2020-048042

Confidential

	None	Mild	Moderate	Severe
Monday	○	○	○	○
Tuesday	○	○	○	○
Wednesday	○	○	○	○
Thursday	○	○	○	○
Friday	○	○	○	○
Saturday	○	○	○	○
Sunday	○	○	○	○

Deterioration of eyesight

	None	Mild	Moderate	Severe
Monday	○	○	○	○
Tuesday	○	○	○	○
Wednesday	○	○	○	○
Thursday	○	○	○	○
Friday	○	○	○	○
Saturday	○	○	○	○
Sunday	○	○	○	○

Rash - All Over

	None	Mild	Moderate	Severe
Monday	○	○	○	○
Tuesday	○	○	○	○
Wednesday	○	○	○	○
Thursday	○	○	○	○
Friday	○	○	○	○
Saturday	○	○	○	○
Sunday	○	○	○	○

Rash - Local

	None	Mild	Moderate	Severe
Monday	○	○	○	○
Tuesday	○	○	○	○
Wednesday	○	○	○	○
Thursday	○	○	○	○
Friday	○	○	○	○
Saturday	○	○	○	○
Sunday	○	○	○	○

Diarrhoea (even mild)

Hayward A, *et al. BMJ Open* 2021; 11:e048042. doi: 10.1136/bmjopen-2020-048042

Confidential

	None	Mild	Moderate	Severe
Monday	○	○	○	○
Tuesday	○	○	○	○
Wednesday	○	○	○	○
Thursday	○	○	○	○
Friday	○	○	○	○
Saturday	○	○	○	○
Sunday	○	○	○	○

Vomiting (being sick)

	None	Mild	Moderate	Severe
Monday	○	○	○	○
Tuesday	○	○	○	○
Wednesday	○	○	○	○
Thursday	○	○	○	○
Friday	○	○	○	○
Saturday	○	○	○	○
Sunday	○	○	○	○

Nausea (feeling sick)

	None	Mild	Moderate	Severe
Monday	○	○	○	○
Tuesday	○	○	○	○
Wednesday	○	○	○	○
Thursday	○	○	○	○
Friday	○	○	○	○
Saturday	○	○	○	○
Sunday	○	○	○	○

Abdominal pain (not including menstrual pain)

	None	Mild	Moderate	Severe
Monday	○	○	○	○
Tuesday	○	○	○	○
Wednesday	○	○	○	○
Thursday	○	○	○	○
Friday	○	○	○	○
Saturday	○	○	○	○
Sunday	○	○	○	○

Hayward A, *et al. BMJ Open* 2021; 11:e048042. doi: 10.1136/bmjopen-2020-048042

COVID-19 testing for [go_arm_1][hh1_fname] [go_arm_1][hh1_sname]

[go_arm_1][hh1_fname] [go_arm_1][hh1_sname]: Have you sought COVID-19 testing for this illness via the NHS, government or your employer?	◯ Yes<br>◯ No
[go_arm_1][hh1_fname] [go_arm_1][hh1_sname]: You said you tried to get tested for COVID-19 via the NHS, government or your employer for this illness. Did you get tested?	◯ Yes<br>◯ No
[go_arm_1][hh1_fname] [go_arm_1][hh1_sname]: Why did you not get an NHS test for COVID-19?	◯ I was advised the test was not needed<br>◯ I am waiting for an NHS test kit to arrive by post<br>◯ I could not get to a testing centre<br>◯ I felt better so decided not to get tested<br>◯ Other - please specify
Other (please specify):	________________________________
[go_arm_1][hh1_fname] [go_arm_1][hh1_sname]: How long was the gap between your symptoms starting and you having an NHS COVID-19 test?	◯ Same day<br>◯ Next day<br>◯ 2 days later<br>◯ 3 to 4 days later<br>◯ 5 to 7 days later<br>◯ More than a week
What was the result of that test?	◯ Positive for COVID-19<br>◯ Negative for COVID-19<br>◯ The result was unclear<br>◯ Still awaiting result
[go_arm_1][hh1_fname] [go_arm_1][hh1_sname]: How long was the gap between your symptoms starting and you getting the NHS test results?	◯ Same day<br>◯ Next day<br>◯ 2 days later<br>◯ 3 to 4 days later<br>◯ 5 to 7 days later<br>◯ More than a week

Confidential

Tracing - [go_arm_1][hh1_fname] [go_arm_1][hh1_sname]

If you had an illness that may be COVID-19 you should follow national advice on reporting this illness and seeking care. We will always have a link to the latest advice on the Virus Watch website.

[go_arm_1][hh1_fname] [go_arm_1][hh1_sname]: Have you reported your illness to any organisation other than Virus Watch?<br>Select all that apply	☐ I did not report my illness to any other organisation<br>☐ Yes to the NHS Contact tracing app<br>☐ Yes to Google/Android contact tracing app<br>☐ Yes to the NHS Test and Trace service<br>☐ Yes to my employer<br>☐ Yes to my GP<br>☐ Yes to the NHS 111 online coronavirus service<br>☐ Yes to the general NHS 111 service<br>☐ Other (please specify)
Other (please specify)	__________________________________
[go_arm_1][hh1_fname] [go_arm_1][hh1_sname]: Why did you not report your illness?	☐ I did not think it was COVID-19<br>☐ I didn't feel ill enough to need help<br>☐ I didn't know where to report it<br>☐ I didn't know I was supposed to report it<br>☐ I didn't want to self isolate<br>☐ I didn't want my contacts to have to self isolate<br>☐ I didn't want others to know that I might have COVID-19
[go_arm_1][hh1_fname] [go_arm_1][hh1_sname]: From the time two days before your illness started to now, have you had direct or close contact with anyone OTHER THAN HOUSEHOLD MEMBERS?	☐ Non-Household Direct Contacts - these are people you had direct physical contact with or with whom you exchanged at least a few words within a 2 metre distance (e.g. a handshake, embracing, kissing, contact sports).<br>☐ Non-Household Close contacts - these are people who were within 2 metres of you for 15 minutes or more but who you did not speak to or touch.<br>☐ I did not have direct or close contact with anybody other than household members
How many people did you have DIRECT CONTACT with other than household members?<br>Please enter digits only, e.g. '5' and not 'five'	__________________________________
How many people did you have CLOSE CONTACT with other than household members?<br>Please enter digits only, e.g. '5' and not 'five'	__________________________________
[go_arm_1][hh1_fname] [go_arm_1][hh1_sname]: Has anyone contacted you to ask about who you have been in contact with prior to or during your illness(contact tracing)?<br>Please select all that apply	☐ No<br>☐ Yes - my GP<br>☐ Yes - the NHS Test and Trace System<br>☐ Yes - telephone advisory service<br>☐ Yes - through an online form<br>☐ Yes - through an app<br>☐ Yes - through my employer<br>☐ Yes - through my place of education<br>☐ Yes - the local public team<br>☐ Other - please specify
Other please specify:	__________________________________

Hayward A, *et al. BMJ Open* 2021; 11:e048042. doi: 10.1136/bmjopen-2020-048042

[go_arm_1][hh1_fname] [go_arm_1][hh1_sname]: Was anyone other than a household member asked to self-isolate because of contact with you?	◯ Yes - and I know how many were asked to self isolate<br>◯ Yes - but I don't know how many were asked to self isolate<br>◯ Nobody was asked to self isolate<br>◯ I don't know if anyone was asked to self isolate
How many people other than household contacts were asked to self-isolate because of contact with you?<br>Please enter digits only, e.g. '5' and not 'five'	__________________________________

Health Advice / Consultation - [go_arm_1][hh1_fname] [go_arm_1][hh1_sname]

[go_arm_1][hh1_fname] [go_arm_1][hh1_sname]: You should follow official national advice about what to do if you have symptoms of COVID-19. We will always include a link to the latest advice on the Virus Watch website.

[go_arm_1][hh1_fname] [go_arm_1][hh1_sname]: Did the illness lead you (or someone else on your behalf) to seek advice about your symptoms this week?
This includes advice from: NHS 111 PharmaciesNurses or doctorsThe internetFriends and family

○ Yes
○ No

Where was the advice or information sought from?

☐ NHS 111 COVID-19 website
☐ NHS 111 COVID-19 phone line
☐ COVID-19 testing centre
☐ Internet (e.g. WebMD , NHS choices)
☐ Pharmacist
☐ GP (by phone)
☐ GP (visit to practice)
☐ GP (online (including video))
☐ Walk-in centre
☐ A&E
☐ Hospital
☐ Friends or family
☐ Other (please specify)

Other (Please Specify): __________________________________

On which days was the medical advice or information sought from these sources Please check all that that apply on each day

	Monday	Tuesday	Wednesday	Thursday	Friday	Saturday	Sunday
Internet (e.g. WebMD , NHS choices)	☐	☐	☐	☐	☐	☐	☐
NHS 111 COVID-19 phone line	☐	☐	☐	☐	☐	☐	☐
NHS 111 COVID-19 website	☐	☐	☐	☐	☐	☐	☐
Pharmacist	☐	☐	☐	☐	☐	☐	☐
GP (by phone)	☐	☐	☐	☐	☐	☐	☐
GP (visit to practice)	☐	☐	☐	☐	☐	☐	☐
GP (online (including video))	☐	☐	☐	☐	☐	☐	☐
Walk-in centre	☐	☐	☐	☐	☐	☐	☐
COVID-19 testing centre	☐	☐	☐	☐	☐	☐	☐
A&E	☐	☐	☐	☐	☐	☐	☐
Hospital	☐	☐	☐	☐	☐	☐	☐

Confidential

Medication - [go_arm_1][hh1_fname] [go_arm_1][hh1_sname]

[go_arm_1][hh1_fname] [go_arm_1][hh1_sname]: Did your symptoms lead to taking any medicines during the survey week?
This includes prescribed medicines, medicine brought at the chemist or shops, or vitamin supplements

◯ Yes
◯ No

On which days did you take the medicines:
Please select all that apply

	Mon	Tues	Wed	Thurs	Fri	Sat	Sun
Paracetamol	☐	☐	☐	☐	☐	☐	☐
Ibuprofen, nurofen, diclofenac, naproxen or other NSAID	☐	☐	☐	☐	☐	☐	☐
Aspirin	☐	☐	☐	☐	☐	☐	☐
Antibiotics	☐	☐	☐	☐	☐	☐	☐
Cold or flu remedies - over the counter	☐	☐	☐	☐	☐	☐	☐
Vitamin Supplements	☐	☐	☐	☐	☐	☐	☐
Other (please describe)	☐	☐	☐	☐	☐	☐	☐

Please specify:

____

Hayward A, *et al. BMJ Open* 2021; 11:e048042. doi: 10.1136/bmjopen-2020-048042

Confidential

Isolation and Infection Control - [go_arm_1][hh1_fname]

[go_arm_1][hh1_fname] [go_arm_1][hh1_sname]: During the past week, on which days did you:

	Mon	Tues	Wed	Thurs	Fri	Sat	Sun	None
Leave the house/flat or garden	☐	☐	☐	☐	☐	☐	☐	☐
Wear a facemask or face covering outside the home	☐	☐	☐	☐	☐	☐	☐	☐
Sleep in a room with no one else in it?	☐	☐	☐	☐	☐	☐	☐	☐
Wear a face mask or face covering at home?	☐	☐	☐	☐	☐	☐	☐	☐
Have a meal with other members of your household?	☐	☐	☐	☐	☐	☐	☐	☐
Watch television with other members of your household?	☐	☐	☐	☐	☐	☐	☐	☐

[go_arm_1][hh1_fname] [go_arm_1][hh1_sname]: During your illness, what best describes your approach to preventing spread of infection in the household?

- ◯ I did not think my illness was COVID-19, so I did not take any special precautions
- ◯ I thought it would spread whatever I did so did not take special precautions
- ◯ I was worried about the illness spreading to others in the household so did what I could to stop this
- ◯ None of the above

[go_arm_1][hh1_fname] [go_arm_1][hh1_sname]: Since your illness started, on which days did OTHER MEMBERS OF YOUR HOUSEHOLD:

	Mon	Tues	Wed	Thur	Fri	Sat	Sun	None
Wear a face mask or face covering when in the same room as you	☐	☐	☐	☐	☐	☐	☐	☐
Leave the house/flat or garden at all	☐	☐	☐	☐	☐	☐	☐	☐
Go to work	☐	☐	☐	☐	☐	☐	☐	☐
Wear a facemask or face covering outside the home	☐	☐	☐	☐	☐	☐	☐	☐

[go_arm_1][hh1_fname] [go_arm_1][hh1_sname]: Why did other household members leave the house?
Please select all that apply

- ☐ They did not think my symptoms were related to COVID-19
- ☐ They did not think there was any need for people without symptoms to stay at home
- ☐ They needed to go out to earn money
- ☐ They needed to go out to an important meeting
- ☐ Other reason

During your illness, how frequently have you washed your hands thoroughly and regularly with soap and water	◯ Not at all<br>◯ 1 or 2 times a day<br>◯ 3 or 4 times a day<br>◯ 5 or 6 times a day<br>◯ 7 or 8 times a day<br>◯ 9 or 10 times a day<br>◯ More than 10 times a day
During your illness, on average, how frequently have you (or someone else) disinfected surfaces you might touch?<br>Such as door knobs or hard surfaces	◯ Several times a day<br>◯ Daily<br>◯ Less than daily<br>◯ Never

[go_arm_1][hh1_fname] [go_arm_1][hh1_sname]: During your illness, how frequently have you:

	Not applicable	Almost always	Most of the time	Sometimes	Rarely	Never
Washed your hands after blowing your nose, sneezing or coughing	◯	◯	◯	◯	◯	◯
Used tissues when sneezing or coughing	◯	◯	◯	◯	◯	◯
Put tissues in the bin immediately after use	◯	◯	◯	◯	◯	◯

Thank You

Please click submit to continue

Hayward A, *et al. BMJ Open* 2021; 11:e048042. doi: 10.1136/bmjopen-2020-048042
