# Supplementary material for: Risk factors, symptom reporting, healthcare-seeking behaviour and adherence to public health guidance: protocol for Virus Watch, a prospective community cohort study
Source: BMJ Open. 2021 Jun 23;11(6):e048042. doi: 10.1136/bmjopen-2020-048042 (PMC8230990; doi:10.1136/bmjopen-2020-048042)
Supplement: Author's manuscript [file bmjopen-2020-048042.draft_revisions.pdf]

BMJ Open is committed to open peer review. As part of this commitment we make the peer review history of every article we publish publicly available.

When an article is published we post the peer reviewers' comments and the authors' responses online. We also post the versions of the paper that were used during peer review. These are the versions that the peer review comments apply to.

The versions of the paper that follow are the versions that were submitted during the peer review process. They are not the versions of record or the final published versions. They should not be cited or distributed as the published version of this manuscript.

BMJ Open is an open access journal and the full, final, typeset and author-corrected version of record of the manuscript is available on our site with no access controls, subscription charges or pay-per-view fees (http://bmjopen.bmj.com).

If you have any questions on BMJ Open's open peer review process please email info.bmjopen@bmj.com

BMJ Open

Risk factors, symptom reporting, healthcare-seeking behaviour and adherence to public health guidance: protocol for Virus Watch, a prospective community cohort study

Journal:	*BMJ Open*
Manuscript ID	bmjopen-2020-048042
Article Type:	Protocol
Date Submitted by the Author:	22-Dec-2020
Complete List of Authors:	Hayward, Andrew; University College London, Institute of Epidemiology and Health Care<br>Fragaszy, Ellen; University College London, Centre for Public Health Data Science, Institute of Health Informatics; LSHTM, Department of Infectious Disease Epidemiology<br>Kovar, Jana; University College London, Institute of Epidemiology and Health Care<br>Nguyen, Vincent; University College London, Institute of Epidemiology and Health Care; University College London, Centre for Public Health Data Science, Institute of Health Informatics<br>Beale, Sarah; University College London, Institute of Epidemiology and Health Care; University College London, Centre for Public Health Data Science, Institute of Health Informatics<br>Byrne, Thomas; University College London, Centre for Public Health Data Science, Institute of Health Informatics<br>Aryee, Anna; University College London, Centre for Public Health Data Science, Institute of Health Informatics<br>Hardelid, Pia; University College London, Centre for Paediatric Epidemiology and Biostatistics, Institute of Child Health<br>Wijlaars, Linda; UCL, Population, Policy and Practice; University College London, Primary Care and Population Health<br>Fong, Wing Lam Erica; University College London, Centre for Public Health Data Science, Institute of Health Informatics<br>Geismar, Cyril; University College London, Institute of Epidemiology and Health Care; University College London, Centre for Public Health Data Science, Institute of Health Informatics<br>Patel, Parth; University College London, Centre for Public Health Data Science, Institute of Health Informatics<br>Shrotri, Madhumita; University College London, Centre for Public Health Data Science, Institute of Health Informatics<br>Navaratnam, Annalan M D; University College London, Institute of Epidemiology and Health Care; University College London, Centre for Public Health Data Science, Institute of Health Informatics<br>Nastouli, Eleni ; Francis Crick Institute; University College London, Department of Population, Policy and Practice<br>Spyer, Moira; Francis Crick Institute<br>Killingley, Ben; University of Nottingham School of Medicine; University College London Hospital

Cox, Ingemar; University College London, Department of Computer Science
Lampos, Vasileios; University College London, Department of Computer Science
McKendry, Rachel; University College London, London Centre for Nanotechnology and Division of Medicine
Liu, Yunzhe; University College London, SpaceTimeLab, Civil, Environmental and Geomatic Engineering.
Cheng, Tao; University College London, SpaceTimeLab, Department of Civil, Environmental and Geomatic Engineering
Johnson, Anne; University College London, Institute for Global Health
Michie, Susan; University College London, Centre for Behaviour Change
Gibbs, Jo; University College London, Institute for Global Health
Gilson, Richard; University College London, Institute for Global Health
Rodger, Alison; University College London, Department of Infection and Population Health; Royal Free London NHS Foundation Trust
Aldridge, Robert; University College London, Centre for Public Health Data Science, Institute of Health Informatics

Keywords: COVID-19, Public health < INFECTIOUS DISEASES, Epidemiology < INFECTIOUS DISEASES

SCHOLARONE™
Manuscripts

I, the Submitting Author has the right to grant and does grant on behalf of all authors of the Work (as defined in the below author licence), an exclusive licence and/or a non-exclusive licence for contributions from authors who are: i) UK Crown employees; ii) where BMJ has agreed a CC-BY licence shall apply, and/or iii) in accordance with the terms applicable for US Federal Government officers or employees acting as part of their official duties; on a worldwide, perpetual, irrevocable, royalty-free basis to BMJ Publishing Group Ltd ("BMJ") its licensees and where the relevant Journal is co-owned by BMJ to the co-owners of the Journal, to publish the Work in this journal and any other BMJ products and to exploit all rights, as set out in our licence.

The Submitting Author accepts and understands that any supply made under these terms is made by BMJ to the Submitting Author unless you are acting as an employee on behalf of your employer or a postgraduate student of an affiliated institution which is paying any applicable article publishing charge ("APC") for Open Access articles. Where the Submitting Author wishes to make the Work available on an Open Access basis (and intends to pay the relevant APC), the terms of reuse of such Open Access shall be governed by a Creative Commons licence – details of these licences and which Creative Commons licence will apply to this Work are set out in our licence referred to above.

Other than as permitted in any relevant BMJ Author's Self Archiving Policies, I confirm this Work has not been accepted for publication elsewhere, is not being considered for publication elsewhere and does not duplicate material already published. I confirm all authors consent to publication of this Work and authorise the granting of this licence.

For peer review only

Risk factors, symptom reporting, healthcare-seeking behaviour and adherence to public health guidance: protocol for Virus Watch, a prospective community cohort study

Andrew Hayward[1], Ellen Fragaszy[2,3], Jana Kovar[1], Vincent Nguyen[1,2], Sarah Beale[1,2], Thomas Byrne[2], Anna Aryee[2], Pia Hardelid[4], Linda Wijlaars[4], Wing Lam Erica Fong[2], Cyril Geismar[1,2], Parth Patel[2], Madhumita Shrotri[2], Annalan M D Navaratnam[1,2], Eleni Nastouli[5,6], Moira Spyer[6,], Ben Killingley[7,8], Ingemar Cox[9], Vasileios Lampos[9],Rachel A McKendry[10], Yunzhe Liu[11], Tao Cheng[11], Anne M Johnson[13], Susan Michie[12], Jo Gibbs[13], Richard Gilson[13], Alison Rodger[13,14], Robert W Aldridge[2]

Affiliations

1 Institute of Epidemiology and Health Care, University College London, London, UK

2 Centre for Public Health Data Science, Institute of Health Informatics, University College London, UK.

3 Department of Infectious Disease Epidemiology, London School of Hygiene and Tropical Medicine, Keppel Street, London, UK.

4 UCL Great Ormond Street Institute of Child Health, London, UK.

5 Department of Population, Policy and Practice, UCL Great Ormond Street Institute of Child Health, London, UK.

6 Francis Crick Institute, London, UK.

7 Health Protection and Influenza Research Group, Division of Epidemiology and Public Heath, University of Nottingham School of Medicine, Nottingham, United Kingdom.

8 University College London Hospital, London, United Kingdom.

9 Department of Computer Science, University College London, London, UK.

10 London Centre for Nanotechnology and Division of Medicine, London, UCL.

11 SpaceTimeLab, Department of Civil, Environmental and Geomatic Engineering, University College London, London, UK.

12 Centre for Behaviour Change, University College London, London, UK.

13 Institute for Global Health, University College London, London, UK.

14 Royal Free London NHS Foundation Trust, London, UK.

For peer review only

Corresponding Author:

Andrew Hayward, Institute of Epidemiology and Health Care, University College London, London, WC1E 7HB, UK

Emails:

Andrew Hayward a.hayward@ucl.ac.uk, Ellen Fragaszy ellen.fragaszy@ucl.ac.uk , Jana Kovar j.kovar@ucl.ac.uk, Vincent Nguyen vincent.nguyen.11@ucl.ac.uk, Sarah Beale sarah.beale.19@ucl.ac.uk, Thomas Byrne t.byrne@ucl.ac.uk, Anna Aryee a.aryee@ucl.ac.uk, Pia Hardelid p.hardelid@ucl.ac.uk, Linda Wijlaars linda.wijlaars@ucl.ac.uk, Wing Lam Erica Fong erica.fong.16@ucl.ac.uk, Cyril Geismar cyril.geismar.16@ucl.ac.uk, Parth Patel parth.patel@ucl.ac.uk, Madhumita Shrotri madhumita.shrotri.09@ucl.ac.uk, Annalan M D Navaratnam a.navaratnam@ucl.ac.uk, Eleni Nastouli e.nastouli@ucl.ac.uk, Moira Spyer moira.spyer@ucl.ac.uk, Ben Killingley ben.killingley@nhs.net, Ingemar Cox i.cox@ucl.ac.uk, Vasileios Lampos v.lampos@ucl.ac.uk, Rachel A McKendry r.a.mckendry@ucl.ac.uk Tao Cheng tao.cheng@ucl.ac.uk, Anne M Johnson anne.johnson@ucl.ac.uk, Susan Michie s.michie@ucl.ac.uk, Jo Gibbs jo.gibbs@ucl.ac.uk, Richard Gilson r.gilson@ucl.ac.uk, Alison Rodger alison.rodger@ucl.ac.uk, Robert W Aldridge r.aldridge@ucl.ac.uk

Word count: 3975

For peer review only

Abstract

Introduction: The Coronavirus (COVID-19) Pandemic has caused significant global mortality and impacted lives around the world. Virus Watch aims to provide evidence on which public health approaches are most likely to be effective in reducing transmission and impact of the virus, and will investigate community incidence, symptom profiles, and transmission of COVID-19 in relation to population movement and behaviours.

Methods and analysis: Virus Watch is a household community cohort study of acute respiratory infections in England & Wales and will run from June 2020 to Sept 2021. The study aims to recruit 42,500 people, including 12,500 from minority ethnic backgrounds, for an online survey cohort. Nested within this larger study will be a sub-cohort of 10,000 individuals, including 3,000 people from minority ethnic backgrounds. This cohort of 10,000 people will have full blood serology taken between October 2020 and January 2021 and repeat serology between May 2021 and August 2021. Participants will also post self-administered nasal swabs for PCR assays of SARS-CoV-2 and will follow one of three different PCR testing schedules based upon symptoms.

Ethics and dissemination: This study has been approved by the Hampstead NHS Health Research Authority Ethics Committee. Ethics approval number – 20/HRA/2320. We are monitoring participant queries and using these to refine methodology where necessary, and are providing summaries of our preliminary findings to inform public health action by working through our partnerships with our study advisory group, Public Health England, NHS and Government Scientific Advisory panels.

Keywords: COVID-19; UK; cohort study; epidemiology

Strengths and limitations of this study

For peer review only

1. Virus Watch is a large national household community cohort study of the occurrence and risk factors for COVID-19 infection that aims to recruit 42,500 people, including 12,500 from minority ethnic backgrounds.
2. Virus Watch is designed to estimate incidence of PCR confirmed COVID-19 in those with respiratory and non-respiratory presentations and the incidence of hospitalisation among PCR confirmed COVID-19 cases.
3. Virus Watch will measure effectiveness and impact of recommended COVID-19 control measures including testing, isolation, social distancing, respiratory and hand hygiene measures on risk of respiratory infection.
4. Only households with a lead householder able to speak English were able to take part in the study up until December 2020. From Jan 2021, translations of the online survey will be implemented for individuals recruited from this point onwards.
5. Only households of up to six people were eligible for inclusion.

Introduction

The Coronavirus disease 2019 (COVID-19) pandemic has caused millions of deaths and impacted lives around the world with the closure of schools, workplaces, and limitations on freedom of movement. Vaccines and effective scalable treatments for COVID-19 are being discovered, but whilst these are still being approved and further refined, we will need to rely on other measures to stop the spread of COVID-19. We will also require studies to examine their effectiveness as they are implemented across England and Wales.

COVID-19 transmission in the UK has started to increase since the end of August 2020. Governments, including those of the UK devolved nations, are adopting a wide range of control measures to limit the spread of infection. These include isolation of people with COVID-19 symptoms and their household contacts, widespread testing and contact tracing, digital contact tracing using mobile phone apps, broad social distancing measures, and local control measures. Environmental cleaning, hand hygiene and face mask use are also advised.

For peer review only

Much of our current knowledge of COVID-19 comes from observations at the more severe end of the disease spectrum, in hospitalised patients and individuals who die having tested positive for the disease.[1–3] Although large-scale studies of prevalence of PCR positive infection and seroprevalence have been established, there is currently limited information on symptom profiles through the course of illness in non hospitalised populations, children, social and behavioural risk factors for infection, strength and duration of immunity, household and community transmission risk, and population behaviours during periods of wellness and illness (including social contacts, use of public spaces, testing behaviours, isolation, mask use, hand and respiratory hygiene). This information can only be gathered accurately through prospective large-scale community cohorts. Our experience of the MRC/Wellcome Flu Watch study[4,5] and ESRC Bug Watch[6] study has allowed us to rapidly establish a national household cohort study of 42,500 individuals.

Virus Watch aims to provide evidence on which public health approaches are most likely to be effective in reducing the spread and impact of the virus and will investigate community incidence, symptom profiles, and transmission of COVID-19 in relation to population movement and behaviour.

For peer review only

Methods

Study design and setting

Virus Watch is a household community cohort study of acute respiratory infections in England and Wales covering the second and potential subsequent waves of the COVID-19 pandemic. The study period will be 1st June 2020 to 30th Sept 2021. The study aims to recruit 42,500 individuals, including 12,500 from minority ethnic backgrounds for an online survey cohort (study 1). Nested within this larger study will be a sub-cohort of 10,000 individuals (study 2), including 3,000 people from minority ethnic backgrounds. Participants in this laboratory sub-cohort will be selected based on their geographical distance away from one of our blood taking clinics; either a 10km radius from a clinic in cities, or a 20km radius in rural areas. Participants will be balanced to be representative of the UK population for sex, age and region. Figure 1 provides an overview of the study design.

Figure 1. Overview of cohort recruitment and data collection for the Virus Watch household community cohort study.

Households self-select into the study if they live in England and Wales and all members of a household need to consent to take part in the study to meet our inclusion criteria. Households need to have an internet connection on a phone, tablet or computer, email, and, up until the end of November 2020, at least one adult household member that can read English. From December 2020 onwards, online surveys will be translated into multiple languages. A household is defined as one or more people (not necessarily related) whose usual residence (4 days/week or more) is at the same address. These householders share cooking facilities, a living room or sitting room or dining area.

For peer review only

Primary outcomes

Study 1: Online Survey Cohort

1. Incidence of respiratory infection symptoms, including COVID-19 disease case definitions.
2. Effectiveness and impact of recommended COVID-19 control measures including testing, isolation, social distancing, respiratory and hand hygiene measures on risk of respiratory infection.
3. Frequency of adherence with public-health recommendations for these control measures.
4. Proportion of community infections that result in hospital admissions and death.

Study 2: Laboratory testing sub-cohort

1. Incidence of PCR confirmed COVID-19.
2. Incidence of PCR confirmed COVID-19 in those with non-respiratory presentations.
3. Incidence of hospitalisation among PCR confirmed COVID-19 cases.
4. Proportion of individuals with SARS-CoV-2 antibodies acquired through natural infection to pandemic coronavirus.
5. Proportion of individuals with cross-reacting antibodies to seasonal coronaviruses acquiring (or not) SARS-CoV-2.
6. Household secondary attack rates.
7. Protective effect of antibodies on infection and re-infection as well as the severity and spectrum of presentation.

Recruitment

We will use the Royal Mail Post Office Address File to generate a list of residential address lists from which households can be sampled and sent Virus Watch recruitment postcards to. The proposed initial sample design is a single-stage stratified probability sample where implicit stratification is employed to benefit from the precision gains that

For peer review only

stratified sampling can bring. Within each region, residential addresses are sorted by (a) quintiles of Index of Multiple Deprivation 2019 (IMD), (b) within quintiles by Local Authorities, (c) postcodes and (d) address. We will perform this in the 9 Government Office Regions of England as well as Wales (10 study regions in total).

We will assess recruitment rates and the representativeness of this initial sample following the mail out of 50,000 postcards. If recruitment is lower than expected or under-representative of the national population, we will redesign our recruitment campaign to include a range of methods in order to build the cohort. This mixed recruitment strategy will be flexible and use a variety of methods including social media, study leaflet drops, text messaging, personalised letters and incentives. Social media adverts will be used to inform individuals about the study and direct them to our website http://ucl-virus-watch.net/ where they can read the participant information sheets and consent to taking part. Digital invitations will also be created for sharing via WhatsApp. Text messages and postal letters inviting patients from their General Practitioner clinics will be organised via Local Clinical Research Networks.[7] We will also work with trusted community partners and religious organisations to promote recruitment into the study.

In order for a household to be enrolled, they will require an internet connection (Wi-Fi, fixed or on a mobile phone), email address, and all household members must agree to take part. Households will nominate a lead householder who will submit study questionnaires. Up until December 2020, the lead householder will need to be able to read English to support other household members in survey completion. From December 2020 onwards, online surveys will be translated into multiple languages and this will no longer be an inclusion criteria. A household is defined as one or more people (not necessarily related) whose usual residence (4 days/week or more) is at the same address. These householders share cooking facilities, and may share a living room or sitting room or dining area if available. Households with more than six members will not be eligible for the study - this criteria was set due to limitations of the REDCap survey infrastructure which did not function correctly when attempting to work with household sizes of greater than six during our pilot testing of the survey.

For peer review only

Virus Watch is powered for our primary aims in study 2 and the estimation of population-level symptomatic COVID-19 attack rate over time. Based on an estimated clinical attack rate of 30% of whom 20% need hospitalisation, and 0.5% die we expect the following number of outcome events in our cohort of 10,000 individuals in study 2: 3000 COVID-19 illnesses, 600 hospitalised cases, and 15 deaths. At one month into the outbreak we would be able to detect a 1.7-fold greater risk of disease in a population subgroup that constitutes 1/5 of the population, and by 2 months the detectable relative risk would be only 1.2. At one month we could detect a 4% hospital admission rate amongst cases with 95% CI of 0.5-6.8, and by 2 months the confidence intervals would narrow to 3.1-4.1. We have used estimates of the expected number of events over time to provide an indication of the fact that the cohort is sufficiently large to provide valuable information through the course of the pandemic. Sample size calculations have been informed by a realistic assessment of what we can achieve based on our previous experience[4,6]. For the serology cohort of 3000 people from minority ethnic backgrounds we assume a modest design effect (DE) due to household and geographical clustering, and 500 participants for six different minority ethnic backgrounds to enable the measurement of a cumulative incidence of 10% with 95% confidence intervals of 3% by each minority ethnic group.

Participant materials and incentives

Participant information sheets will be held on our study website. In order to participate, the whole household must take part. Each adult participant will need to read through study information, and provide online informed consent for themselves and any children they are legally responsible for. Children aged 6-9 and 10-15 years respectively will also be asked to read through age specific study participant information sheets and provide online informed assent. For children aged 5 and under, parents/guardians will consent on their behalf. Copies of translated consent questions will be provided where possible for those unable to read English. Informed consent data will be securely stored in UCL's Data Safe Haven which has been certified to the ISO27001 information security standard and conforms to NHS Digital's Data Security and Protection Toolkit.

For peer review only - http://bmjopen.bmj.com/site/about/guidelines.xhtml

Local study teams will re-consent participants face to face, prior to undertaking blood sampling, and adult participants in study 2 will be offered a £10 voucher to reimburse travel costs. We will seek ethical approval for the use of recruitment incentives if levels of recruitment are lower than expected.

Data collection and follow-up

Study 1: Online Survey Cohort

For peer review only

The online survey cohort will collect data and follow up participants through six different sources. Survey data will be collected using Research Electronic Data Capture (REDCap) electronic data capture tools hosted on the UCL Data Safe Haven.[8] REDCap is a secure, web-based application for research studies. The UCL Data Safe Haven provides a technical solution for storing, handling and analysing identifiable data. It has been certified to the ISO27001 information security standard and conforms to NHS Digital's Data Security and Protection Toolkit .

1) Baseline Survey. The Lead Householder will be asked to complete an online baseline survey for each member of their household. Information collected includes: demographics, occupation, income, ethnicity, country of birth, year of entry to UK, chronic medical conditions, medications, pregnancy status, vaccines, mode of transport to work, any previous contact with someone with COVID-19, previous symptoms of COVID-19-like illness and infection-prevention behaviours such as social distancing and hand hygiene.

2) Illness Surveys. Participants will be followed-up weekly via an email with a link to an illness survey. This is a weekly survey of the presence or absence of symptoms that could indicate COVID-19 disease including respiratory, general infection symptoms or gastrointestinal symptoms. During illness, prospective daily symptom recording, quality of life, health seeking behaviour (NHS 111, GP in person, GP by phone, A&E, Pharmacy, Hospital), treatments, and NHS investigations will be recorded. This survey will also include any respiratory and hygiene measures, self-isolation, activities and social contact, travel and

face mask use. The survey includes questions to the household on activities undertaken in the week prior to symptom onset. The weekly survey will also be used to capture test results received from outside the study and requests to self isolate eg. via the UK Test-Trace-Isolate system.

3) Monthly Surveys. A number of questions will be asked every month. The monthly surveys also provide flexibility to ask additional questions (eg. behavioural changes) to reflect any new government directives on social distancing, testing, contact tracing, and vaccine delivery. Core questions will also allow us to follow up reasons for any non-response in a given month- (e.g. because of illness, hospitalisation or holiday). We will also ask about online health information seeking, social distancing, including recent (week before) contacts, activities, places visited and hand & respiratory hygiene. We will ask about finances, employment, and mental health to see how the COVID-19 response is affecting participants' wellbeing and ability to work. We will ask about access to healthcare for non–COVID-19 health problems to explore the indirect health impacts of the pandemic. We will ask about any COVID-19 PCR or antibody test results performed outside the study and not already reported through baseline surveys. We will ask about influenza vaccine uptake and COVID-19 vaccination intentions and uptake.

4) Data Linkage. NHS Digital will undertake quarterly data linkage between cohort 1 and Hospital Episode Statistics (HES), which includes admitted patient and critical care episodes, outpatient department bookings, and emergency care contacts. This linkage will also include Office for National Statistics mortality data and virology testing data routinely collected by Public Health England, Public Health Wales, and the Department of Health and Social Care through 'Pillar 1' (testing in hospital patients and health and care workers) and 'Pillar 2' (community testing). These data sources will be linked to the cohort using name, NHS numbers, dates of birth and postal address. Identifying variables will be removed before the linked data are transferred back to UCL for analysis. These data linkages will continue for up to 5 years after the end of the study as we anticipate COVID-19 will become a recurring winter infection and we wish to understand its impact

For peer review only

on health services in subsequent years. These linkage studies will identify any participants that have been admitted to hospital or died due to causes that could be directly or indirectly linked to the COVID-19 pandemic. Indirect causes include those related to limitations in healthcare access during the pandemic. Reductions in the use of routine health services will also be monitored via linkage to HES data.

5) Geo-location Tracking. All adult participants will be asked about optional consent to use a secure geo-location tracking app (Tracker for ArcGIS) installed on their mobile phone for the duration of the study.

6) Home antibody finger prick tests. 5000 members of the online cohort who are not part of the Laboratory testing sub-cohort (including 2500 minority ethnic and 2500 White British people) will be offered home finger prick antibody testing kits as soon as available after the first wave of the pandemic and after the second wave of the pandemic.

Study 2: Laboratory testing sub-cohort

All participants agreeing to take part in the main cohort (study 1) will be asked to provide consent to be contacted and invited to participate in one of the three laboratory testing sub-groups. This will enable a cohort of 10,000 individuals selected from the main cohort of 42,500 individuals to be maximally representative of the population of England and Wales. All participants taking part in study 2 will be asked to use the national test, trace and isolation system in addition to providing samples as part of Virus Watch.

Study 2 will consist of three groups that will follow different schedules of antibody testing and nasal/throat swabs for PCR testing.

Group 1 (*n*=7000):

For peer review only

With data from this group we aim to identify infection in those with a wide range of respiratory symptoms. Participants will be asked to submit a nose/throat swab if they experience two consecutive days of: fever (>37.8), feeling feverish, or new persistent cough, or loss or altered sense of smell or taste (COVID-19 suspected case definition), or shortness of breath, or ear pain or change in hearing, or sore throat, or sneezing, or blocked nose, or runny nose, or wheeze or sinus pain or congestion (other respiratory manifestations).

Group 2 (*n*=1000):
This group aims to identify the importance of non-respiratory presentations. Participants will be asked to submit a self-taken nasal/throat swab for PCR identification of COVID-19 and other respiratory viruses if:

- Either - two consecutive days of respiratory symptoms (e.g. cough, runny nose, sneezing, shortness of breath, sore throat, blocked nose, sinus pain or congestion, ear pain or change in hearing, wheezing, loss of or altered sense of taste or sense of smell).
- OR – two consecutive days of gastrointestinal symptoms (e.g. diarrhoea/loose stools, abdominal pain, nausea or vomiting, loss of appetite).
- OR - two consecutive days of general infection symptoms (e.g. feeling feverish, having a high temperature, feelings of severe unexplained tiredness, generalised muscle or joint aches)

Group 3 (*n* = 2000):
This group aims to identify the extent of household transmission. Participants will be asked to submit a nose/throat swab if they experience two consecutive days of cough or fever or loss of sense of taste or smell. Household contacts of the index case will also be asked to submit a swab on the same day whether or not they have symptoms.

If any of the swabs indicate SARS-COV-2 infection, all household members will be asked to repeat the swab on Day 7 and Day 14. If there are no new SARS-COV-2 cases in the household arising from swabs on Day 7 and 14 (assumed secondary cases) then all household members will be asked to undertake a home finger prick

For peer review only

antibody test on Day 21. If there is one or more secondary cases in the household then the entire household will be asked to take an additional swab on day 21 and then undertake the fingerprick antibody tests on Day 28.

End of follow-up

Online participant follow-up will end in May 2021 although depending on the progression of COVID-19 we may ask participants to continue in the study for longer. Participants will be sent an exit survey via email which will also ask participants for permission to be contacted for involvement in future related research. Participants will be contacted to arrange a second blood sample collection from May 2021. Follow-up through data linkage with Hospital Episode Statistics and Mortality data will continue for 5 years after the end of the study.

Laboratory testing

Antibody testing

Study 2 will be using two different types of antibody tests. First, full blood serology will be taken between October 2020 and January 2021. We will use experienced health care professionals, including research nurses from the NIHR Clinical Research Networks.[9] Depending on local circumstances, visits to participants' homes to take blood may also be arranged. Children aged 15 years or less can opt out of having their blood taken but will be offered a finger prick antibody test conducted by a healthcare worker instead. All participants from laboratory group 3 will additionally be offered a fingerprick antibody test at the same time as blood taking. From May 2021 until September 2021, we will invite all participants back for full blood tests or, for children who do not wish to have a full bleed, healthcare worker-delivered finger prick based antibody tests.

Families of children who have not been able to attend for a blood test, or for a healthcare worker-delivered finger prick antibody test, will be provided with postal kits to perform these at home. We also plan to use finger prick antibody testing where local clinics are no longer able to undertake full blood tests due to COVID-19 travel

For peer review only - http://bmjopen.bmj.com/site/about/guidelines.xhtml

restrictions. Extremely clinically vulnerable participants will be sent home fingerprick tests instead of being asked to provide a serological sample.

Virus detection

Participants will post swab samples for PCR assays of COVID-19, and subsequent testing for influenza virus, seasonal coronavirus, rhinovirus and respiratory syncytial virus (RSV). When COVID-19 is identified we will also undertake whole genome sequencing of the virus. Samples for COVID-19 diagnostics will be handled and processed according to the NHS and UCL guidance on sample handling during the COVID-19 pandemic.

COVID-19 PCR and serology results will be returned to participants via text and email message systems. These messages will include links to official support, information and advice from NHS and PHE as well as advice on how to interpret results based on current evidence. In laboratory group 3, where positive test results will trigger further testing of the household, the results email will also include details explaining the additional testing requests.

Statistical analysis

Our primary analyses during the winter 2020/21 will focus on estimating age-specific weekly rates of symptoms and PCR-confirmed COVID-19 illness and hospitalisation. For this analysis we will use appropriate regression models that account for clustering by household and we will explore the use of stratification or weighting of the sample by age and region as necessary to give nationally representative estimates. Weekly rates will be expressed per 100,000 person-weeks for ease of comparison with national surveillance data.

We will examine the proportion of the population infected during the first wave (e.g. Feb 2020 to Sept 2020) and second and potentially future pandemics waves. We will estimate the percentage of the population infected by calculating age and wave-specific rates of serological infection and PCR-confirmed disease per 100 person-seasons. A

For peer review only - http://bmjopen.bmj.com/site/about/guidelines.xhtml

person-season will be defined by the epidemic curve in the cohort and therefore rates will account for differential follow-up time during each epidemic peak. In these analyses we will examine risk factors for infection, disease, disease severity and disease transmission.

We will estimate the proportion of serologically confirmed SARS-CoV-2 infections leading to symptomatic disease. First, we will calculate age-adjusted attributable rates of illness due to infection (subtracting rates of respiratory illness in non seroconverters from those in seroconverters). Second, we will measure the proportion of seroconverters with PCR-confirmed COVID-19. Analyses plans will be developed prior to conducting all analyses.

Whilst the study is being conducted, we will produce early, preliminary results for participants, the general public and policy makers in order to inform the public health response to the pandemic. These analyses will be reactive to the epidemiological circumstances and are therefore not defined in this protocol.

Modelling

We will build on our experience of working with PHE, Google, and Microsoft to use anonymous national or subnational aggregate web search engine data[10,11] to monitor the spreading of the disease. We will use our study data as ground-truth to train real-time disease prevalence estimation algorithms. We will annotate GPS tracking data into standard categories including time at work and home, social venues, supermarkets, hospitals, GPs, and transport mode for incorporation in classical epidemiological analyses. Integrating the linked survey data, we will develop multi-level spatio-temporal transmission models predicting the impact of various social distancing strategies.

For peer review only

Patient and public involvement

Due to the urgent nature of this study, we did not involve participants in its original design. We have previously conducted PPI to support similar community cohort studies of acute infections using similar methodologies. We have engaged the Young Persons' Advisory Group for research at Great Ormond Street Hospital to provide feedback on our Children's Participant Information Sheets. We will provide opportunities for survey participants to comment on survey methodology at the first monthly survey and consider revisions based on this. At the baseline survey, and each month, we will ask participants what questions are important to them (in relation to COVID-19 epidemiology and response), and what research questions they would like us to answer. We are also monitoring participant queries through our study email address and using these to refine methodology where necessary. We have worked with the Race Equality Foundation and Doctors of the World in advising on the inclusion of people from minority ethnic backgrounds in Virus Watch and have set up an advisory group to inform the ongoing design and dissemination of health equity aspects of Virus Watch. This advisory group (consisting of lay members of the public, community leaders, charities and policy organisation) will guide our health equity analyses and steer us on its implications for people, communities and policy.

Data sharing and access

We aim to share aggregate data from this project on our website and via a "Findings so far" section on our website - https://ucl-virus-watch.net/. We will also be sharing individual record level data with personal identifiers removed on a research data sharing service such as the UK Data Archive. In sharing the data we will work within the principles set out in the UKRI Guidance on best practice in the management of research data. https://www.ukri.org/files/legacy/documents/rcukcommonprinciplesondatapolicy-pdf/. Access to use of the data whilst research is being conducted will be managed by the Chief Investigators (ACH and RWA) in accordance with the principles set out in the UKRI guidance on best practice in the management of research data. It is the intention that the data arising from this research will initially be collected, cleaned and validated

by the UCL research team and once this has been completed will be shared for wider use. We aim to make subsets of the data more rapidly available both on our study website and via the public facing dashboard during the ongoing phase of data collection. In line with Principle 5 of the UKRI guidance on best practice in the management of research data, we plan to release data in batches as they become available or as updated results are published. Individual record data linked using NHS Digital will not be shared, only aggregated results. HES and mortality data may be obtained from a third party and are not publicly available. These data are owned by a third party and can be accessed by researchers applying to the Health and Social Care Information Centre for England. We will put analysis code on publicly available repositories to enable their reuse.

Ethics

This is a national study that has been approved by the Hampstead NHS Health Research Authority Ethics Committee. Ethics approval number – 20/HRA/2320. The study is compliant with the requirements of General Data Protection Regulation (2016/679) and the Data Protection Act (2018). All investigators and study site staff will comply with the requirements of the General Data Protection Regulation (2016/679) with regards to the collection, storage, processing and disclosure of personal information, and will uphold the Act's core principles.

Author Contributions

Roles: Conceptualization (AH, EF, JK, PH, EN, BK, IC, VL, RAMcK, TC, AMJ, SM, JG, RG, AR, RWA) Investigation, Methodology (All authors), Project Administration (AH, EF, JK, VN, SB, TB, AA, PH, LW, WLEF, CG, PP, MSh, AMDN, EN, MSp, RWA) , Writing – Original Draft Preparation (All Authors), Software (VN, TB, SB, RWA), Resources (AH, EF, JK, PH, EN, BK, IC, VL, RAMcK, TC, AMJ, SM, JG, RG, AR, RWA), Writing – Review & Editing (All Authors).

For peer review only

Funding

The research costs for the study have been supported by the MRC Grant Ref: MC_PC 19070 awarded to UCL on 30 March 2020 and MRC Grant Ref: MR/V028375/1 awarded on 17 August 2020. The study also received $15,000 of advertising credit to support a pilot social media recruitment campaign on 18th August 2020.

Competing interests: ACH serves on the UK New and Emerging Respiratory Virus Threats Advisory Group. AMJ was a Governor of Wellcome Trust from 2011-18 and is Chair of the Committee for Strategic Coordination for Health of the Public Research.

References

1 Official UK Coronavirus Dashboard. https://coronavirus.data.gov.uk/ (accessed 30 Nov 2020).

2 Statistics. Statistics » COVID-19 Daily Deaths. https://www.england.nhs.uk/statistics/statistical-work-areas/covid-19-daily-deaths/ (accessed 30 Nov 2020).

3 Coronavirus (COVID-19). https://www.ons.gov.uk/peoplepopulationandcommunity/healthandsocialcare/conditionsanddiseases (accessed 30 Nov 2020).

4 Fragaszy EB, Warren-Gash C, Wang L, *et al.* Cohort Profile: The Flu Watch Study. *Int J Epidemiol* 2017;**46**:e18. doi:10.1093/ije/dyv370

5 Hayward AC, Fragaszy EB, Bermingham A, *et al.* Comparative community burden and severity of seasonal and pandemic influenza: results of the Flu Watch cohort study. *Lancet Respir Med* 2014;**2**:445–54. doi:10.1016/S2213-2600(14)70034-7

6 Smith CM, Conolly A, Fuller C, *et al.* Symptom reporting, healthcare-seeking behaviour and antibiotic use for common infections: protocol for Bug Watch, a prospective community cohort study. *BMJ Open* 2019;**9**:e028676. doi:10.1136/bmjopen-2018-028676

7 Clinical Research Network. https://www.nihr.ac.uk/explore-nihr/support/clinical-research-network.htm (accessed 30 Nov 2020).

8 Harris PA, Taylor R, Thielke R, *et al.* Research electronic data capture (REDCap)--a metadata-driven methodology and workflow process for providing translational research informatics support. *J Biomed Inform* 2009;**42**:377–81. doi:10.1016/j.jbi.2008.08.010

9 Clinical Research Network. https://www.nihr.ac.uk/explore-nihr/support/clinical-research-

For peer review only

network.htm (accessed 30 Nov 2020).

10 Wagner M, Lampos V, Cox IJ, *et al.* The added value of online user-generated content in traditional methods for influenza surveillance. *Sci Rep* 2018;**8**:13963. doi:10.1038/s41598-018-32029-6

11 Lampos V, Majumder MS, Yom-Tov E, *et al.* Tracking COVID-19 using online search. arXiv [cs.SI]. 2020.http://arxiv.org/abs/2003.08086v10

For peer review only

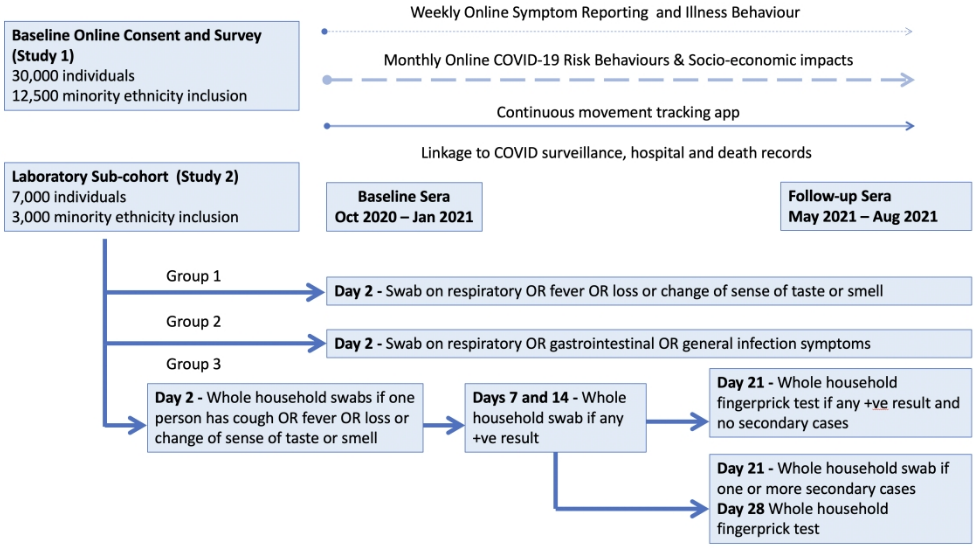

Figure 1. Overview of cohort recruitment and data collection for the Virus Watch household community cohort study.

BMJ Open

Risk factors, symptom reporting, healthcare-seeking behaviour and adherence to public health guidance: protocol for Virus Watch, a prospective community cohort study

Journal:	*BMJ Open*
Manuscript ID	bmjopen-2020-048042.R1
Article Type:	Protocol
Date Submitted by the Author:	29-Mar-2021

Complete List of Authors:	Hayward, Andrew; UCL, Institute of Epidemiology and Health Care<br>Fragaszy, Ellen; UCL, Centre for Public Health Data Science, Institute of Health Informatics; LSHTM, Department of Infectious Disease Epidemiology<br>Kovar, Jana; UCL, Institute of Epidemiology and Health Care<br>Nguyen, Vincent; UCL, Institute of Epidemiology and Health Care; University College London, Centre for Public Health Data Science, Institute of Health Informatics<br>Beale, Sarah; UCL, Institute of Epidemiology and Health Care; University College London, Centre for Public Health Data Science, Institute of Health Informatics<br>Byrne, Thomas; UCL, Centre for Public Health Data Science, Institute of Health Informatics<br>Aryee, Anna; University College London, Centre for Public Health Data Science, Institute of Health Informatics<br>Hardelid, Pia; University College London, Centre for Paediatric Epidemiology and Biostatistics, Institute of Child Health<br>Wijlaars, Linda; UCL, Population, Policy and Practice; University College London, Primary Care and Population Health<br>Fong, Wing Lam Erica; University College London, Centre for Public Health Data Science, Institute of Health Informatics<br>Geismar, Cyril; University College London, Institute of Epidemiology and Health Care; University College London, Centre for Public Health Data Science, Institute of Health Informatics<br>Patel, Parth; University College London, Centre for Public Health Data Science, Institute of Health Informatics<br>Shrotri, Madhumita; University College London, Centre for Public Health Data Science, Institute of Health Informatics<br>Navaratnam, Annalan M D; University College London, Institute of Epidemiology and Health Care; University College London, Centre for Public Health Data Science, Institute of Health Informatics<br>Nastouli, Eleni ; Francis Crick Institute; University College London, Department of Population, Policy and Practice<br>Spyer, Moira; Francis Crick Institute<br>Killingley, Ben; University of Nottingham School of Medicine; University College London Hospital<br>Cox, Ingemar; UCL, Department of Computer Science<br>Lampos, Vasileios; University College London, Department of Computer

	Science<br>McKendry, Rachel; University College London, London Centre for Nanotechnology and Division of Medicine<br>Liu, Yunzhe; University College London, SpaceTimeLab, Civil, Environmental and Geomatic Engineering.<br>Cheng, Tao; University College London, SpaceTimeLab, Department of Civil, Environmental and Geomatic Engineering<br>Johnson, Anne; University College London, Institute for Global Health<br>Michie, Susan; University College London, Centre for Behaviour Change<br>Gibbs, Jo; University College London, Institute for Global Health<br>Gilson, Richard; University College London, Institute for Global Health<br>Rodger, Alison; UCL, Institute for Global Health; Royal Free London NHS Foundation Trust<br>Aldridge, Robert; UCL, Centre for Public Health Data Science, Institute of Health Informatics

**Primary Subject Heading**:	Public health
Secondary Subject Heading:	Epidemiology, Infectious diseases
Keywords:	COVID-19, Public health < INFECTIOUS DISEASES, Epidemiology < INFECTIOUS DISEASES

SCHOLARONE™
Manuscripts

I, the Submitting Author has the right to grant and does grant on behalf of all authors of the Work (as defined in the below author licence), an exclusive licence and/or a non-exclusive licence for contributions from authors who are: i) UK Crown employees; ii) where BMJ has agreed a CC-BY licence shall apply, and/or iii) in accordance with the terms applicable for US Federal Government officers or employees acting as part of their official duties; on a worldwide, perpetual, irrevocable, royalty-free basis to BMJ Publishing Group Ltd ("BMJ") its licensees and where the relevant Journal is co-owned by BMJ to the co-owners of the Journal, to publish the Work in this journal and any other BMJ products and to exploit all rights, as set out in our licence.

The Submitting Author accepts and understands that any supply made under these terms is made by BMJ to the Submitting Author unless you are acting as an employee on behalf of your employer or a postgraduate student of an affiliated institution which is paying any applicable article publishing charge ("APC") for Open Access articles. Where the Submitting Author wishes to make the Work available on an Open Access basis (and intends to pay the relevant APC), the terms of reuse of such Open Access shall be governed by a Creative Commons licence – details of these licences and which Creative Commons licence will apply to this Work are set out in our licence referred to above.

Other than as permitted in any relevant BMJ Author's Self Archiving Policies, I confirm this Work has not been accepted for publication elsewhere, is not being considered for publication elsewhere and does not duplicate material already published. I confirm all authors consent to publication of this Work and authorise the granting of this licence.

For peer review only

Risk factors, symptom reporting, healthcare-seeking behaviour and adherence to public health guidance: protocol for Virus Watch, a prospective community cohort study

Andrew Hayward[1], Ellen Fragaszy[2,3], Jana Kovar[1], Vincent Nguyen[1,2], Sarah Beale[1,2], Thomas Byrne[2], Anna Aryee[2], Pia Hardelid[4], Linda Wijlaars[4], Wing Lam Erica Fong[2], Cyril Geismar[1,2], Parth Patel[2], Madhumita Shrotri[2], Annalan M D Navaratnam[1,2], Eleni Nastouli[5,6], Moira Spyer[6,], Ben Killingley[7,8], Ingemar Cox[9], Vasileios Lampos[9], Rachel A McKendry[10], Yunzhe Liu[11], Tao Cheng[11], Anne M Johnson[13], Susan Michie[12], Jo Gibbs[13], Richard Gilson[13], Alison Rodger[13,14], Robert W Aldridge[2]

Affiliations

1 Institute of Epidemiology and Health Care, University College London, London, UK

2 Centre for Public Health Data Science, Institute of Health Informatics, University College London, UK.

3 Department of Infectious Disease Epidemiology, London School of Hygiene and Tropical Medicine, Keppel Street, London, UK.

4 UCL Great Ormond Street Institute of Child Health, London, UK.

5 Department of Population, Policy and Practice, UCL Great Ormond Street Institute of Child Health, London, UK.

6 Francis Crick Institute, London, UK.

7 Health Protection and Influenza Research Group, Division of Epidemiology and Public Heath, University of Nottingham School of Medicine, Nottingham, United Kingdom.

8 University College London Hospital, London, United Kingdom.

9 Department of Computer Science, University College London, London, UK.

10 London Centre for Nanotechnology and Division of Medicine, London, UCL.

11 SpaceTimeLab, Department of Civil, Environmental and Geomatic Engineering, University College London, London, UK.

12 Centre for Behaviour Change, University College London, London, UK.

13 Institute for Global Health, University College London, London, UK.

14. Royal Free London NHS Foundation Trust, London, UK.

Corresponding Author:

For peer review only

Andrew Hayward, Institute of Epidemiology and Health Care, University College London, London, WC1E 7HB, UK

Emails:

Andrew Hayward a.hayward@ucl.ac.uk, Ellen Fragaszy ellen.fragaszy@ucl.ac.uk , Jana Kovar j.kovar@ucl.ac.uk, Vincent Nguyen vincent.nguyen.11@ucl.ac.uk, Sarah Beale sarah.beale.19@ucl.ac.uk, Thomas Byrne t.byrne@ucl.ac.uk, Anna Aryee a.aryee@ucl.ac.uk, Pia Hardelid p.hardelid@ucl.ac.uk, Linda Wijlaars linda.wijlaars@ucl.ac.uk, Wing Lam Erica Fong erica.fong.16@ucl.ac.uk, Cyril Geismar cyril.geismar.16@ucl.ac.uk, Parth Patel parth.patel@ucl.ac.uk, Madhumita Shrotri madhumita.shrotri.09@ucl.ac.uk, Annalan M D Navaratnam a.navaratnam@ucl.ac.uk, Eleni Nastouli e.nastouli@ucl.ac.uk, Moira Spyer moira.spyer@ucl.ac.uk, Ben Killingley ben.killingley@nhs.net, Ingemar Cox i.cox@ucl.ac.uk, Vasileios Lampos v.lampos@ucl.ac.uk, Rachel A McKendry r.a.mckendry@ucl.ac.uk Tao Cheng tao.cheng@ucl.ac.uk, Anne M Johnson anne.johnson@ucl.ac.uk, Susan Michie s.michie@ucl.ac.uk, Jo Gibbs jo.gibbs@ucl.ac.uk, Richard Gilson r.gilson@ucl.ac.uk, Alison Rodger alison.rodger@ucl.ac.uk, Robert W Aldridge r.aldridge@ucl.ac.uk

Word count: 4,600

For peer review only

Abstract

Introduction: The Coronavirus (COVID-19) Pandemic has caused significant global mortality and impacted lives around the world. Virus Watch aims to provide evidence on which public health approaches are most likely to be effective in reducing transmission and impact of the virus, and will investigate community incidence, symptom profiles, and transmission of COVID-19 in relation to population movement and behaviours.

Methods and analysis: Virus Watch is a household community cohort study of acute respiratory infections in England & Wales and will run from June 2020 to August 2021. The study aims to recruit 50,000 people, including 12,500 from minority ethnic backgrounds, for an online survey cohort and monthly antibody testing using home finger prick kits. Nested within this larger study will be a sub-cohort of 10,000 individuals, including 3,000 people from minority ethnic backgrounds. This cohort of 10,000 people will have full blood serology taken between October 2020 and January 2021 and repeat serology between May 2021 and August 2021. Participants will also post self-administered nasal swabs for PCR assays of SARS-CoV-2 and will follow one of three different PCR testing schedules based upon symptoms.

Ethics and dissemination: This study has been approved by the Hampstead NHS Health Research Authority Ethics Committee. Ethics approval number – 20/HRA/2320. We are monitoring participant queries and using these to refine methodology where necessary, and are providing summaries and policy briefings of our preliminary findings to inform public health action by working through our partnerships with our study advisory group, Public Health England, NHS and Government Scientific Advisory panels.

Keywords: COVID-19; UK; cohort study; epidemiology

Strengths and limitations of this study

For peer review only

1. Virus Watch is a large national household community cohort study of the occurrence and risk factors for COVID-19 infection that aims to recruit 50,000 people, including 12,500 from minority ethnic backgrounds.
2. Virus Watch is designed to estimate incidence of PCR confirmed COVID-19 in those with respiratory and non-respiratory presentations and the incidence of hospitalisation among PCR confirmed COVID-19 cases.
3. Virus Watch will measure effectiveness and impact of recommended COVID-19 control measures including testing, isolation, social distancing, respiratory and hand hygiene measures on risk of respiratory infection.
4. Only households with a lead householder able to speak English were able to take part in the study up until March 2021. From March 2021, translations of the online survey will be implemented for individuals recruited from this point onwards.
5. Only households of up to six people were eligible for inclusion and they were also required to have access to an internet connection. These restrictions will limit the generalisability to large or multigenerational households, and those without access to the internet.

Introduction

The Coronavirus disease 2019 (COVID-19) pandemic has caused millions of deaths and impacted lives around the world with the closure of schools, workplaces, and limitations on freedom of movement. Vaccines and effective scalable treatments for COVID-19 have been developed and whilst these are rolled out across England and Waleswe will need to rely on other measures to stop the spread of COVID-19. We will also require studies to examine their long-term effectiveness as they are implemented across England and Wales.
Governments, including those of the UK devolved nations, are adopting a wide range of control measures to limit the spread of infection. These include isolation of people with COVID-19 symptoms and their household contacts, widespread testing and contact tracing, digital contact tracing using mobile phone apps, broad social distancing

measures, and local control measures. Environmental cleaning, hand hygiene and face mask use are also advised.

Much of our current knowledge of COVID-19 comes from observations at the more severe end of the disease spectrum, in hospitalised patients and individuals who die having tested positive for the disease.[1–3] Although large-scale studies of prevalence of PCR positive infection and seroprevalence have been established, there is currently limited information on symptom profiles through the course of illness in non hospitalised populations, children, social and behavioural risk factors for infection, strength and duration of immunity, household and community transmission risk, and population behaviours during periods of wellness and illness (including social contacts, use of public spaces, testing behaviours, isolation, mask use, hand and respiratory hygiene). This information can only be gathered accurately through prospective large-scale community cohorts. Our experience of the MRC/Wellcome Flu Watch study[4,5] and ESRC Bug Watch[6] study has allowed us to rapidly establish a national household cohort study of 50,000 individuals.

Virus Watch aims to provide evidence on which public health approaches are most likely to be effective in reducing the spread and impact of the virus and will investigate community incidence, symptom profiles, and transmission of COVID-19 in relation to population movement and behaviour.

For peer review only

Methods and analysis

Study design and setting

Virus Watch is a household community cohort study of acute respiratory infections in England and Wales covering the second and potential subsequent waves of the COVID-19 pandemic. The study period will be 1st June 2020 to 31st August 2021 . The study aims to recruit 50,000 individuals, including 12,500 from minority ethnic backgrounds for an online survey cohort (study 1). Nested within this larger study will be a sub-cohort of 10,000 individuals (study 2), including 3,000 people from minority ethnic backgrounds. Participants in this laboratory sub-cohort will be selected based on their geographical distance away from one of our blood taking clinics; either a 10km radius from a clinic in cities, or a 20km radius in rural areas. Participants will be balanced to be representative of the UK population for sex, age and region. Figure 1 provides an overview of the study design.

Households self-select into the study if they live in England and Wales and all members of a household need to consent to take part in the study to meet our inclusion criteria (Appendix 1). Households need to have an internet connection on a phone, tablet or computer, email, and, up until the end of November 2020, at least one adult household member that can read English. From March 2020 onwards, online surveys will be translated into multiple languages. A household is defined as one or more people (not necessarily related) whose usual residence (4 days/week or more) is at the same address. These householders share cooking facilities, a living room or sitting room or dining area.

Primary outcomes

Study 1: Online Survey Cohort

1. Incidence of respiratory infection symptoms, including COVID-19 disease case definitions.

For peer review only

2. Effectiveness and impact of recommended COVID-19 control measures including testing, isolation, social distancing, respiratory and hand hygiene measures on risk of respiratory infection.
3. Frequency of adherence with public-health recommendations for these control measures.
4. Proportion of community infections that result in hospital admissions and death.
5. Vaccine effectiveness against asymptomatic and symptomatic infections.

Study 2: Laboratory testing sub-cohort

1. Incidence of PCR confirmed COVID-19.
2. Incidence of PCR confirmed COVID-19 in those with non-respiratory presentations.
3. Incidence of hospitalisation among PCR confirmed COVID-19 cases.
4. Proportion of individuals with SARS-CoV-2 antibodies acquired through natural infection to pandemic coronavirus.
5. Proportion of individuals with cross-reacting antibodies to seasonal coronaviruses acquiring (or not) SARS-CoV-2.
6. Household secondary attack rates.
7. Protective effect of antibodies on infection and re-infection as well as the severity and spectrum of presentation.

Recruitment

We will use the Royal Mail Post Office Address File to generate a list of residential address lists from which households can be sampled and sent Virus Watch recruitment postcards to. The proposed initial sample design is a single-stage stratified probability sample where implicit stratification is employed to benefit from the precision gains that stratified sampling can bring. Within each region, residential addresses are sorted by (a) quintiles of Index of Multiple Deprivation 2019 (IMD), (b) within quintiles by Local Authorities, (c) postcodes and (d) address. We will perform this in the nine Government Office Regions of England as well as Wales (10 study regions in total).

For peer review only - http://bmjopen.bmj.com/site/about/guidelines.xhtml

We will assess recruitment rates and the representativeness of this initial sample following the mail out of 50,000 postcards. If recruitment is lower than expected or under-representative of the national population, we will redesign our recruitment campaign to include a range of methods in order to build the cohort. This mixed recruitment strategy will be flexible and use a variety of methods including social media, study leaflet drops, text messaging, personalised letters and incentives. Social media adverts will be used to inform individuals about the study and direct them to our website http://ucl-virus-watch.net/ where they can read the participant information sheets and consent to taking part. Digital invitations will also be created for sharing via WhatsApp. Text messages and postal letters inviting patients from their General Practitioner clinics will be organised via Local Clinical Research Networks.[7] We will also work with trusted community partners and religious organisations to promote recruitment into the study.

In order for a household to be enrolled, they will require an internet connection (Wi-Fi, fixed or on a mobile phone), email address, and all household members must agree to take part. Households will nominate a lead householder who will submit study questionnaires. Up until March 2021, the lead householder will need to be able to read English to support other household members in survey completion. From March 2021 onwards, online surveys will be translated into multiple languages and this will no longer be an inclusion criteria. A household is defined as one or more people (not necessarily related) whose usual residence (4 days/week or more) is at the same address. These householders share cooking facilities, and may share a living room or sitting room or dining area if available. Households with more than six members will not be eligible for the study - this criteria was set due to limitations of the REDCap survey infrastructure which did not function correctly when attempting to work with household sizes of greater than six during our pilot testing of the survey.

Virus Watch is powered for our primary aims in study 2 and the estimation of population-level symptomatic COVID-19 attack rate over time. Recruiting a cohort that is representative of the population is time consuming as it requires an initial invitation into a study followed by multiple follow-up contacts encouraging invited individuals to

For peer review only

register. Given the urgency of the public health situation to roll out our study as quickly as possible we chose a different approach whereby we recruit a large cohort of 50,000 individuals and from within that cohort we select a sub-sample for the testing cohort (sub-cohort 1) which is representative of the population in terms of age, sex, ethnicity, region, household size and proportion of households with children. The larger cohort will be important in assessing rates and predictors of less frequent outcomes such as hospitalisation and death. Given recent information of marked ethnicity differences in mortality rates from COVID-19 we also chose to recruit an ethnicity sample designed to be sufficiently large to provide early indicators of whether these differential mortality rates are due to differences in disease incidence or in differences in severity or both.

Power Analysis

The testing sub-cohort is powered for accurate weekly age-specific disease incidence rates to be measured assuming 20-30% clinical attack rate over 18 weeks. With a clinical attack rate of 30% of whom 20% need hospitalisation, and 0.5% die we expect the following number of outcome events in our testing cohort of 10,000 individuals in study 2: 3000 COVID-19 illnesses, 600 hospitalised cases, and 15 deaths. At one month into the outbreak we would be able to detect a 1.7-fold greater risk of disease in a population subgroup that constitutes 1/5 of the population, and by 2 months the detectable relative risk would be only 1.2. At one month we could detect a 4% hospital admission rate amongst cases with 95% CI of 0.5-6.8, and by 2 months the confidence intervals would narrow to 3.1-4.1. We have used estimates of the expected number of events over time to provide an indication of the fact that the cohort is sufficiently large to provide valuable information through the course of the pandemic. Sample size calculations have been informed by a realistic assessment of what we can achieve based on our previous experience[4,6]. For the serology cohort of 3000 people from minority ethnic backgrounds we assume a modest design effect (DE) due to household and geographical clustering, and 500 participants for six different minority ethnic backgrounds would enable the measurement of a cumulative incidence of 10% with 95% confidence intervals of 3% by each minority ethnic group.

Participant materials and incentives

Participant information sheets will be held on our study website (these along with consent forms were translated into 6 languages and a further 3 languages were added from December 2020). In order to participate, the whole household must take part. Each adult participant will need to read through study information, and provide online informed consent for themselves and any children they are legally responsible for. Children aged 6-9 and 10-15 years respectively will also be asked to read through age specific study participant information sheets and provide online informed assent. For children aged 5 and under, parents/guardians will consent on their behalf. Informed consent data will be securely stored in UCL's Data Safe Haven which has been certified to the ISO27001 information security standard and conforms to NHS Digital's Data Security and Protection Toolkit. Local study teams will re-consent participants face to face, prior to undertaking blood sampling, and adult participants in study 2 will be offered a £10 voucher to reimburse travel costs. From February 2021, invitation letters sent by GP clinics will include a £20 voucher for households that agree to take part in the study.

Data collection and follow-up

Study 1: Online Survey Cohort

The online survey cohort will collect data and follow up participants through six different sources. Survey data will be collected using Research Electronic Data Capture (REDCap) electronic data capture tools hosted on the UCL Data Safe Haven (Appendix 2, Appendix 3, Appendix 4).[8] REDCap is a secure, web-based application for research studies. The UCL Data Safe Haven provides a technical solution for storing, handling and analysing identifiable data. It has been certified to the ISO27001 information security standard and conforms to NHS Digital's Data Security and Protection Toolkit .

1) Baseline Survey. The Lead Householder will be asked to complete an online baseline survey for each

member of their household. Information collected includes: demographics, occupation, income, ethnicity, country of birth, year of entry to UK, chronic medical conditions, medications, pregnancy status, vaccines, mode of transport to work, any previous contact with someone with COVID-19, previous symptoms of COVID-19-like illness and infection-prevention behaviours such as social distancing and hand hygiene.

2) Illness Surveys. Participants will be followed-up weekly via an email with a link to an illness survey. This is a weekly survey of the presence or absence of symptoms that could indicate COVID-19 disease including respiratory, general infection symptoms or gastrointestinal symptoms. During illness, prospective daily symptom recording, quality of life, health seeking behaviour (NHS 111, GP in person, GP by phone, A&E, Pharmacy, Hospital), treatments, and NHS investigations will be recorded. This survey will also include any respiratory and hand hygiene measures, self-isolation, activities and social contact, travel and face mask use. Questions around behavioural interventions, such as mask wearing and social distancing, aim to reflect the context and frequency/degree to which behaviours are practiced according to governmental and public health guidelines and relevant scientific literature. The survey includes questions to the household on activities undertaken in the week prior to symptom onset. The weekly survey will also be used to capture test results received from outside the study and requests to self isolate eg. via the UK Test-Trace-Isolate system. The weekly survey will also ask about participants' COVID-19 vaccination uptake, including their date of vaccination, dose (i.e. first or second), and which vaccine was administered.

3) Monthly Surveys. A number of questions will be asked every month. The monthly surveys also provide flexibility to ask additional questions (eg. behavioural changes) to reflect any new government directives on social distancing, testing, contact tracing, and vaccine delivery. Core questions will also allow us to follow up reasons for any non-response in a given month- (e.g. because of illness, hospitalisation or holiday). We will also ask about online health information seeking, social distancing, including recent (week before) contacts, activities, places visited and hand & respiratory hygiene. As with the

For peer review only

weekly questionnaire, questions around behavioural practices will reflect governmental and public health guidelines and the scientific literature; monthly questionnaires will also investigate barriers and enablers to health-related behaviours using purpose-developed questionnaires based on the Capability, Motivation, Opportunity, Behaviour (COM-B) model. We will also ask about finances, employment, and mental health to see how the COVID-19 response is affecting participants' wellbeing and ability to work. We will ask about access to healthcare for non–COVID-19 health problems to explore the indirect health impacts of the pandemic. We will ask about any COVID-19 PCR or antibody test results performed outside the study and not already reported through baseline surveys. We will ask about influenza vaccine uptake and COVID-19 vaccination intentions.

4) Data Linkage. NHS Digital will undertake quarterly data linkage between cohort 1 and Hospital Episode Statistics (HES), which includes admitted patient and critical care episodes, outpatient department bookings, and emergency care contacts. This linkage will also include Office for National Statistics mortality data, COVID-19 vaccination records, and virology testing data routinely collected by Public Health England, Public Health Wales, and the Department of Health and Social Care through 'Pillar 1' (testing in hospital patients and health and care workers) and 'Pillar 2' (community testing). These data sources will be linked to the cohort using name, NHS numbers, dates of birth and postal address. Identifying variables will be removed before the linked data are transferred back to UCL for analysis. These data linkages will continue for up to 5 years after the end of the study as we anticipate COVID-19 will become a recurring winter infection and we wish to understand its impact on health services in subsequent years. These linkage studies will identify any participants that have been admitted to hospital or died due to causes that could be directly or indirectly linked to the COVID-19 pandemic. Indirect causes include those related to limitations in healthcare access during the pandemic. Reductions in the use of routine health services will also be monitored via linkage to HES data.

5) Geo-location Tracking. All adult participants will be asked about optional consent to use a secure geo-location

For peer review only

tracking app (Tracker for ArcGIS) installed on their mobile phone for the duration of the study.

6) Monthly antibody testing using home finger prick kits. Adults aged 18 years and over enrolled in the online survey cohort, with the exception of those in laboratory testing sub-cohort Group 3, will be offered monthly antibody testing starting from February 2021 and continuing until the end of the study, using home finger prick kits for self-collection of capillary blood samples. Those aged under 18 and living with adults enrolled in monthly antibody testing will continue completing online surveys. Monthly antibody testing (February-August 2021) will utilise CE-marked at home finger prick kits designed to collect small-volume (400-600 microlitres) capillary blood samples. Samples are self-collected by adult participants and returned to a UKAS-accredited laboratory via pre-paid post, where they will be tested for anti-Nucleocapsid and anti-Spike antibodies using validated electro-chemiluminescence immunoassays.

Study 2: Laboratory testing sub-cohort

All participants agreeing to take part in the main cohort (study 1) will be asked to provide consent to be contacted and invited to participate in one of the three laboratory testing sub-groups. This will enable a cohort of 10,000 individuals selected from the main cohort of 50,000 individuals to be maximally representative of the population of England and Wales. All participants taking part in study 2 will be asked to use the national test, trace and isolation system in addition to providing samples as part of Virus Watch.

Study 2 will consist of three groups that will follow different schedules of antibody testing and nasal/throat swabs for PCR testing.

Group 1 (*n*=7000):
With data from this group we aim to identify infection in those with a wide range of respiratory symptoms. Participants will be asked to submit a nose/throat swab if they experience two consecutive days of: fever (>37.8), feeling feverish, or new persistent

For peer review only

cough, or loss or altered sense of smell or taste (COVID-19 suspected case definition), or shortness of breath, or ear pain or change in hearing, or sore throat, or sneezing, or blocked nose, or runny nose, or wheeze or sinus pain or congestion (other respiratory manifestations).

Group 2 (*n*=1000):

This group aims to identify the importance of non-respiratory presentations. Participants will be asked to submit a self-taken nasal/throat swab for PCR identification of COVID-19 and other respiratory viruses if:

- Either - two consecutive days of respiratory symptoms (e.g. cough, runny nose, sneezing, shortness of breath, sore throat, blocked nose, sinus pain or congestion, ear pain or change in hearing, wheezing, loss of or altered sense of taste or sense of smell).
- OR – two consecutive days of gastrointestinal symptoms (e.g. diarrhoea/loose stools, abdominal pain, nausea or vomiting, loss of appetite).
- OR - two consecutive days of general infection symptoms (e.g. feeling feverish, having a high temperature, feelings of severe unexplained tiredness, generalised muscle or joint aches)

Group 3 (*n* = 2000):

This group aims to identify the extent of household transmission. Participants will be asked to submit a nose/throat swab if they experience two consecutive days of cough or fever or loss of sense of taste or smell. Household contacts of the index case will also be asked to submit a swab on the same day whether or not they have symptoms.

If any of the swabs indicate SARS-COV-2 infection, all household members will be asked to repeat the swab on Day 7 and Day 14. If there are no new SARS-COV-2 cases in the household arising from swabs on Day 7 and 14 (assumed secondary cases) then all household members will be asked to undertake a home finger prick antibody test on Day 21. If there is one or more secondary cases in the household then the entire household will be asked to take an additional swab on day 21 and then undertake the fingerprick antibody tests on Day 28.

For peer review only - http://bmjopen.bmj.com/site/about/guidelines.xhtml

End of follow-up

Online participant follow-up will end in August 2021 for households enrolled in monthly antibody testing, and in May 2021 for others, although, depending on the progression of COVID-19, we may ask participants to continue in the study for longer. Participants will be sent an exit survey. Participants will be contacted to arrange a second blood sample collection from April 2021. Follow-up through data linkage with Hospital Episode Statistics, COVID-19 vaccination records, and Mortality data will continue for 5 years after the end of the study.

Laboratory testing

Antibody testing

Study 2 will be using two different types of antibody tests. First, full blood serology will be taken between October 2020 and January 2021. We will use experienced health care professionals, including research nurses from the NIHR Clinical Research Networks.[9] Depending on local circumstances, visits to participants' homes to take blood may also be arranged. Children aged 15 years or less can opt out of having their blood taken but will be offered a finger prick antibody test conducted by a healthcare worker instead. All participants from laboratory group 3 will additionally be offered a finger prick antibody test at the same time as blood taking. From April 2021 until July 2021, we will invite all participants back for full blood tests or, for children who do not wish to have a full bleed, healthcare worker-delivered finger prick based antibody tests.

Families of children who have not been able to attend for a blood test, or for a healthcare worker-delivered finger prick antibody test, will be provided with postal kits to perform these at home. We also plan to use finger prick antibody testing where local clinics are no longer able to undertake full blood tests due to COVID-19 travel restrictions. Extremely clinically vulnerable participants will be sent home fingerprick tests instead of being asked to provide a serological sample.

For peer review only

Virus detection

Participants will post swab samples for PCR assays of COVID-19, and subsequent testing for influenza virus, seasonal coronavirus, rhinovirus and respiratory syncytial virus (RSV). When COVID-19 is identified we will also undertake whole genome sequencing of the virus. Samples for COVID-19 diagnostics will be handled and processed according to the NHS and UCL guidance on sample handling during the COVID-19 pandemic.

COVID-19 PCR and serology results will be returned to participants via email message systems. These messages will include links to official support, information and advice from NHS and PHE as well as advice on how to interpret results based on current evidence. In laboratory group 3, where positive test results will trigger further testing of the household, the results email will also include details explaining the additional testing requests. We be not asking for inconclusive COVID-19 PCR results to be repeated

Statistical analysis

Our primary analyses during the winter 2020/21 will focus on estimating age-specific weekly rates of symptoms and risk factors for PCR-confirmed COVID-19 illness and hospitalisation. For these analyses we will use poisson regression models that account for clustering by household using robust standard errors and we will explore the use of stratification or weighting of the sample by age and region as necessary to give nationally representative estimates. Weekly rates will be expressed per 100,000 person-weeks for ease of comparison with national surveillance data.

We will examine the proportion of the population infected during the first wave (e.g. Feb 2020 to Sept 2020) and second and potentially future pandemics waves. We will estimate the percentage of the population infected by calculating age and wave-specific rates of serological infection and PCR-confirmed disease per 100 person-seasons using poisson regression with robust standard errors to account for household-level clustering. A person-season will be defined by the epidemic curve in the cohort and

For peer review only

therefore rates will account for differential follow-up time during each epidemic peak. In these analyses we will examine risk factors for infection, disease, disease severity and disease transmission.

We will estimate the proportion of serologically confirmed SARS-CoV-2 infections leading to symptomatic disease. First, we will calculate age-adjusted attributable rates of illness due to infection (subtracting rates of respiratory illness in non seroconverters from those in seroconverters). Second, we will measure the proportion of seroconverters with PCR-confirmed COVID-19. Analyses plans will be developed prior to conducting all analyses.

We will estimate vaccine effectiveness against asymptomatic SARS-CoV-2 infections and against symptomatic COVID-19 using anti-Nucleocapsid seroconversion, positive PCR testing, and self-reported symptoms data. We will utilise both time-to-event and test-negative analytical frameworks. Using quantitative antibody data, we will assess the dynamics of anti-spike antibodies over time and the relationship between antibody titres and the risk of infection.

Whilst the study is being conducted, we will produce early, preliminary results and analyses for participants, the general public, government scientific advisory groups and policy makers in order to inform the public health response to the pandemic. These analyses will be reactive to the epidemiological circumstances and are therefore not defined in this protocol.

Modelling

We will build on our experience of working with PHE, Google, and Microsoft to use anonymous national or subnational aggregate web search engine data[10,11] to monitor the spreading of the disease. We will use our study data as ground-truth to train real-time disease prevalence estimation algorithms. We will annotate GPS tracking data into standard categories including time at work and home, social venues, supermarkets, hospitals, GPs, and transport mode for incorporation in classical epidemiological analyses. Integrating the linked survey data, we will develop a predictive spatio-

For peer review only

temporal transmission model to investigate the impact of various social distancing strategies.

Missing data

We have several strategies that attempt to address the issue of missing data. First, we have sought to minimise the amount and impact of missing data for key outcomes and exposures through the study design. For example, for a number of our primary outcomes (PCR+ illness, hospitalisation and death) and exposures (vaccination) we collect data both as self-reported and through data linkage with the relevant national datasets and registries. Second, we sought to minimise missing serological and virus-watch specific swabbing outcomes in adults by making willingness to provide relevant specimens a prerequisite to study registration. Third, we know from our experience of previous community cohort studies of acute infections (Flu Watch[4] and Bug Watch[6]) that response to weekly surveys (where our symptom data is collected) is high at around 75%, which we believe is achieved by keeping these weekly data collections simple and quick to complete. We have aimed to replicate this approach in Virus Watch. Forth, for important missing baseline demographic data (e.g. age and sex) we have created follow-up surveys to try and collect missing data at a later time in time. Fifth, where necessary, we will address missing data in our analyses and use multiple imputation methods if appropriate.

Patient and Public Involvement

Due to the urgent nature of this study, we did not involve participants in its original design. We have previously conducted PPI to support similar community cohort studies of acute infections using similar methodologies. We have engaged the Young Persons' Advisory Group for research at Great Ormond Street Hospital to provide feedback on our Children's Participant Information Sheets. We have worked with the Race Equality Foundation and Doctors of the World in advising on the inclusion of people from minority ethnic backgrounds in Virus Watch and have set up an advisory group to inform the ongoing design and dissemination of health equity aspects of Virus Watch. They

were not asked to assess the burden of the intervention and time required to participate in the research due to the urgent nature of setting the study up. This advisory group (consisting of lay members of the public, community leaders, charities and policy organisation) will guide our health equity analyses and steer us on its implications for people, communities and policy. The advisory group will also help us prioritise what information and results to share, when, and in what format.

Ethics and dissemination

This is a national study that has been approved by the Hampstead NHS Health Research Authority Ethics Committee. Ethics approval number – 20/HRA/2320. The study is compliant with the requirements of General Data Protection Regulation (2016/679) and the Data Protection Act (2018). All investigators and study site staff will comply with the requirements of the General Data Protection Regulation (2016/679) with regards to the collection, storage, processing and disclosure of personal information, and will uphold the Act's core principles.

We will provide opportunities for survey participants to comment on survey methodology at the first monthly survey and consider revisions based on this. We are also monitoring participant queries through our study email address and using these to refine methodology where necessary.

Data sharing and access

We aim to share aggregate data from this project on our website and via a "Findings so far" section on our website - https://ucl-virus-watch.net/. We will also be sharing individual record level data with personal identifiers removed on a research data sharing service such as the Office of National Statistics Secure Research Service.[12] In sharing the data we will work within the principles set out in the UKRI Guidance on best practice in the management of research data.[13] Access to use of the data whilst research is being conducted will be managed by the Chief Investigators (ACH and

For peer review only

RWA) in accordance with the principles set out in the UKRI guidance on best practice in the management of research data. It is the intention that the data arising from this research will initially be collected, cleaned and validated by the UCL research team and once this has been completed will be shared for wider use. We aim to make subsets of the data more rapidly available both on our study website and via the public facing dashboard during the ongoing phase of data collection. In line with Principle 5 of the UKRI guidance on best practice in the management of research data, we plan to release data in batches as they become available or as updated results are published. Individual record data linked using NHS Digital will not be shared, only aggregated results. HES and mortality data may be obtained from a third party and are not publicly available. These data are owned by a third party and can be accessed by researchers applying to the Health and Social Care Information Centre for England. We will put analysis code on publicly available repositories to enable their reuse.

Author Contributions

Roles: Conceptualization (AH, EF, JK, PH, EN, BK, IC, VL, RAMcK, TC, AMJ, SM, JG, RG, AR, RWA) Investigation, Methodology (All authors), Project Administration (AH, EF, JK, VN, SB, TB, AA, PH, LW, WLEF, CG, PP, MSh, AMDN, EN, MSp, RWA) , Writing – Original Draft Preparation (All Authors), Software (VN, TB, SB, RWA), Resources (AH, EF, JK, PH, EN, BK, IC, VL, RAMcK, TC, YL, AMJ, SM, JG, RG, AR, RWA), Writing – Review & Editing (All Authors).

Funding

The research costs for the study have been supported by the MRC Grant Ref: MC_PC 19070 awarded to UCL on 30 March 2020 and MRC Grant Ref: MR/V028375/1 awarded on 17 August 2020. The study also received $15,000 of Facebook advertising credit to support a pilot social media recruitment campaign on 18th August 2020.

For peer review only

Competing interests: ACH serves on the UK New and Emerging Respiratory Virus Threats Advisory Group. AMJ was a Governor of Wellcome Trust from 2011-18 and is Chair of the Committee for Strategic Coordination for Health of the Public Research.

Figure legends

Figure 1. Overview of cohort recruitment, PCR swabbing schedules and data collection for the Virus Watch household community cohort study.

For peer review only

References

1 Official UK Coronavirus Dashboard. https://coronavirus.data.gov.uk/ (accessed 30 Nov 2020).

2 Statistics. Statistics » COVID-19 Daily Deaths. https://www.england.nhs.uk/statistics/statistical-work-areas/covid-19-daily-deaths/ (accessed 30 Nov 2020).

3 Coronavirus (COVID-19). https://www.ons.gov.uk/peoplepopulationandcommunity/healthandsocialcare/conditionsanddiseases (accessed 30 Nov 2020).

4 Fragaszy EB, Warren-Gash C, Wang L, *et al.* Cohort Profile: The Flu Watch Study. *Int J Epidemiol* 2017;**46**:e18. doi:10.1093/ije/dyv370

5 Hayward AC, Fragaszy EB, Bermingham A, *et al.* Comparative community burden and severity of seasonal and pandemic influenza: results of the Flu Watch cohort study. *Lancet Respir Med* 2014;**2**:445–54. doi:10.1016/S2213-2600(14)70034-7

6 Smith CM, Conolly A, Fuller C, *et al.* Symptom reporting, healthcare-seeking behaviour and antibiotic use for common infections: protocol for Bug Watch, a prospective community cohort study. *BMJ Open* 2019;**9**:e028676. doi:10.1136/bmjopen-2018-028676

7 Clinical Research Network. https://www.nihr.ac.uk/explore-nihr/support/clinical-research-network.htm (accessed 30 Nov 2020).

8 Harris PA, Taylor R, Thielke R, *et al.* Research electronic data capture (REDCap)--a metadata-driven methodology and workflow process for providing translational research informatics support. *J Biomed Inform* 2009;**42**:377–81. doi:10.1016/j.jbi.2008.08.010

9 Clinical Research Network. https://www.nihr.ac.uk/explore-nihr/support/clinical-research-

network.htm (accessed 30 Nov 2020).

10 Wagner M, Lampos V, Cox IJ, *et al.* The added value of online user-generated content in traditional methods for influenza surveillance. *Sci Rep* 2018;**8**:13963. doi:10.1038/s41598-018-32029-6

11 Lampos V, Majumder MS, Yom-Tov E, *et al.* Tracking COVID-19 using online search. arXiv [cs.SI]. 2020.http://arxiv.org/abs/2003.08086v10

12 Accessing secure research data as an accredited researcher. https://www.ons.gov.uk/aboutus/whatwedo/statistics/requestingstatistics/approvedresearcherscheme (accessed 12 Mar 2021).

13 UKRI. Guidance on best practice in the management of research data. https://www.ukri.org/wp-content/uploads/2020/10/UKRI-020920-GuidanceBestPracticeManagementResearchData.pdf

For peer review only

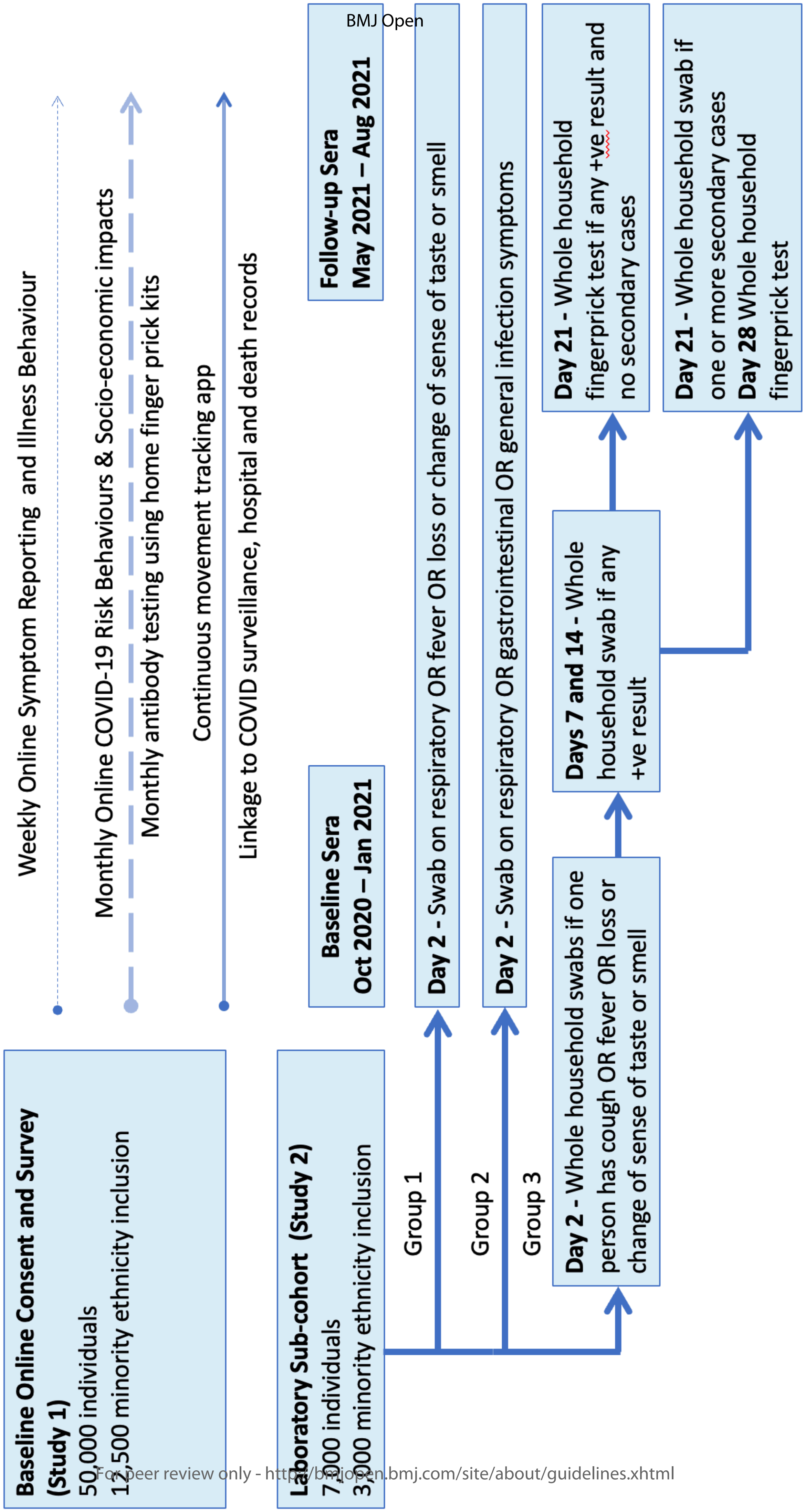

For peer review only - http://bmjopen.bmj.com/site/about/guidelines.xhtml

Appendix 1 - Study inclusion and exclusion criteria:

Inclusion:

- Households self-select into the study.
- Participants need to join as a household (all must take part).
- They need to have internet connection on a phone, tablet or computer, email and up until end of Feb 2021 at least one adult that can read English.
- From December 2020, online surveys will be translated into multiple languages.

Exclusion Criteria

We will exclude participants if:

- Number of householders exceeds 6.
- Those without internet connection on a phone, tablet or computer, or an email address available to them as they will be unable to register
- There is no adult in the household who can read English (from March 2021 this will no longer be an exclusion criteria)
- A household is defined as one or more people (not necessarily related) whose usual residence (4days/week or more) is at the same address. These householders share cooking facilities, a living room or sitting room or dining area.

For peer review only

Baseline for Lead Householder

Baseline Survey for [hh1_fname] [hh1_sname]

alive ______________________

The baseline survey collects some basic information about each person participating in Virus Watch. We will ask about each household member in turn, please ask each adult to complete their own information and help children to complete their sections or complete them for them

[hh1_fname] [hh1_sname]: At birth you were described as?
- ◯ Male
- ◯ Female
- ◯ Intersex
- ◯ Prefer not to say

[hh1_fname] [hh1_sname]: Do you know your NHS Number? This can usually be found on an NHS letter
- ◯ Yes
- ◯ No

[hh1_fname] [hh1_sname]: What is your NHS Number? ______________________

[hh1_fname] [hh1_sname]: Where is your place of Birth?
- ◯ United Kingdom
- ◯ Other

[hh1_fname] [hh1_sname]: When did you first come to live in the UK (approximately)? DD/MM/YYYY ______________________

For peer review only

[hh1_fname] [hh1_sname]: Please specify where you were born:

◯ Afghanistan
◯ Albania
◯ Algeria
◯ American Samoa
◯ Andorra
◯ Angola
◯ Anguilla
◯ Antarctica
◯ Antigua And Barbuda
◯ Argentina
◯ Armenia
◯ Aruba
◯ Australia
◯ Austria
◯ Azerbaijan
◯ Bahamas
◯ Bahrain
◯ Bangladesh
◯ Barbados
◯ Belarus
◯ Belgium
◯ Belize
◯ Benin
◯ Bermuda
◯ Bhutan
◯ Bolivia
◯ Bosnia And Herzegovina
◯ Botswana
◯ Bouvet Island
◯ Brazil
◯ British Indian Ocean Territory
◯ Brunei Darussalam
◯ Bulgaria
◯ Burkina Faso
◯ Burundi
◯ Cambodia
◯ Cameroon
◯ Canada
◯ Cape Verde
◯ Cayman Islands
◯ Central African Republic
◯ Chad
◯ Chile
◯ China
◯ Christmas Island
◯ Cocos (keeling) Islands
◯ Colombia
◯ Comoros
◯ Congo
◯ Congo, The Democratic Republic Of The
◯ Cook Islands
◯ Costa Rica
◯ Cote D'ivoire
◯ Croatia
◯ Cuba
◯ Cyprus
◯ Czech Republic
◯ Denmark
◯ Djibouti
◯ Dominica
◯ Dominican Republic
◯ East Timor
◯ Ecuador
◯ Egypt
◯ El Salvador
◯ Equatorial Guinea
◯ Eritrea
◯ Estonia
◯ Ethiopia

For peer review only

◯ Falkland Islands (malvinas)
◯ Faroe Islands
◯ Fiji
◯ Finland
◯ France
◯ French Guiana
◯ French Polynesia
◯ French Southern Territories
◯ Gabon
◯ Gambia
◯ Georgia
◯ Germany
◯ Ghana
◯ Gibraltar
◯ Greece
◯ Greenland
◯ Grenada
◯ Guadeloupe
◯ Guam
◯ Guatemala
◯ Guinea
◯ Guinea-bissau
◯ Guyana
◯ Haiti
◯ Heard Island And Mcdonald Islands
◯ Holy See (vatican City State)
◯ Honduras
◯ Hong Kong
◯ Hungary
◯ Iceland
◯ India
◯ Indonesia
◯ Iran, Islamic Republic Of
◯ Iraq
◯ Ireland
◯ Israel
◯ Italy (Italian Republic)
◯ Jamaica
◯ Japan
◯ Jordan
◯ Kazakstan
◯ Kenya
◯ Kiribati
◯ Korea, Democratic People's Republic Of
◯ Korea, Republic Of
◯ Kosovo
◯ Kuwait
◯ Kyrgyzstan
◯ Lao People's Democratic Republic
◯ Latvia
◯ Lebanon
◯ Lesotho
◯ Liberia
◯ Libyan Arab Jamahiriya
◯ Liechtenstein
◯ Lithuania
◯ Luxembourg
◯ Macau
◯ Macedonia, The Former Yugoslav Republic Of
◯ Madagascar
◯ Malawi
◯ Malaysia
◯ Maldives
◯ Mali
◯ Malta
◯ Marshall Islands
◯ Martinique
◯ Mauritania
◯ Mauritius
◯ Mayotte
◯ Mexico

For peer review only

◯ Micronesia, Federated States Of
◯ Moldova, Republic Of
◯ Monaco
◯ Mongolia
◯ Montserrat
◯ Montenegro
◯ Morocco
◯ Mozambique
◯ Myanmar
◯ Namibia
◯ Nauru
◯ Nepal
◯ Netherlands
◯ Netherlands Antilles
◯ New Caledonia
◯ New Zealand
◯ Nicaragua
◯ Niger
◯ Nigeria
◯ Niue
◯ Norfolk Island
◯ Northern Mariana Islands
◯ Norway
◯ Oman
◯ Pakistan
◯ Palau
◯ Palestinian Territory, Occupied
◯ Panama
◯ Papua New Guinea
◯ Paraguay
◯ Peru
◯ Philippines
◯ Pitcairn
◯ Poland
◯ Portugal
◯ Puerto Rico
◯ Qatar
◯ Reunion
◯ Romania
◯ Russian Federation
◯ Rwanda
◯ Saint Helena
◯ Saint Kitts And Nevis
◯ Saint Lucia
◯ Saint Pierre And Miquelon
◯ Saint Vincent And The Grenadines
◯ Samoa
◯ San Marino
◯ Sao Tome And Principe
◯ Saudi Arabia
◯ Senegal
◯ Serbia
◯ Seychelles
◯ Sierra Leone
◯ Singapore
◯ Slovakia
◯ Slovenia
◯ Solomon Islands
◯ Somalia
◯ South Africa
◯ South Georgia And The South Sandwich Islands
◯ Spain
◯ Sri Lanka
◯ Sudan
◯ Suriname
◯ Svalbard And Jan Mayen
◯ Swaziland
◯ Sweden
◯ Switzerland
◯ Syrian Arab Republic
◯ Taiwan, Province Of China

For peer review only

	◯ Tajikistan<br>◯ Tanzania, United Republic Of<br>◯ Thailand<br>◯ Togo<br>◯ Tokelau<br>◯ Tonga<br>◯ Trinidad And Tobago<br>◯ Tunisia<br>◯ Turkey<br>◯ Turkmenistan<br>◯ Turks And Caicos Islands<br>◯ Tuvalu<br>◯ Uganda<br>◯ Ukraine<br>◯ United Arab Emirates<br>◯ United Kingdom<br>◯ United States<br>◯ United States Minor Outlying Islands<br>◯ Uruguay<br>◯ Uzbekistan<br>◯ Vanuatu<br>◯ Venezuela<br>◯ VietNam<br>◯ Virgin Islands, British<br>◯ Virgin Islands, U.s.<br>◯ Wallis And Futuna<br>◯ Western Sahara<br>◯ Yemen<br>◯ Zambia<br>◯ Zimbabwe
[hh1_fname] [hh1_sname]: What is your ethnic group?	◯ White - English/ Welsh/ Scottish/ Northern Irish/ British<br>◯ White - Irish<br>◯ White - Gypsy or Irish Traveller<br>◯ Any other white background (please describe)<br>◯ Asian/ Asian British - Indian<br>◯ Asian/ Asian British - Pakistani<br>◯ Asian/ Asian British - Bangladeshi<br>◯ Asian/ Asian British - Chinese<br>◯ Any other Asian/ Asian British background (please describe)<br>◯ Black African<br>◯ Black Caribbean<br>◯ Any other Black/ African/ Caribbean background (please describe)<br>◯ Arab<br>◯ Any other ethnic group (please describe)<br>◯ Mixed/ multiple ethnic groups - White and Black Caribbean<br>◯ Mixed/ multiple ethnic groups - White and Black African<br>◯ Mixed/ multiple ethnic groups - White and Asian<br>◯ Any other mixed/ multiple ethnic background (please describe)<br>◯ Prefer not to say
[hh1_fname] [hh1_sname]: Please describe your ethnic group:	__________________________________
[hh1_fname] [hh1_sname]: Are you pregnant?	◯ Yes<br>◯ No

For peer review only

What trimester of pregnancy are you in?	◯ Less than 12 Weeks<br>◯ 12 Weeks to 24 Weeks<br>◯ More than 24 weeks
Contact details	
[hh1_fname] [hh1_sname]: What is your mobile phone number?<br>This is so we can call you to make blood taking appointments and send your test results if you are selected for the swabbing part of the study.<br>If you do not have a mobile phone, please enter your landline phone number, and we will seek alternative arrangements to send your results if you are selected for the swabbing part of the study.	__________________
Address Line 1:<br>This is to send you swabs (if you are chosen by the study team to partake in the swabbing study)	__________________
Address Line 2:	__________________
Address Line 3:	__________________
Post Code:	__________________

For peer review only

Medical Background for [hh1_fname] [hh1_sname]

People's health can influence the severity of COVID illness, we want to find out more about this.

[hh1_fname] [hh1_sname]: Please provide your general practitioner's details:
This will allow us to link to your information to hospital records more accurately.

Name of Surgery: ______________________

Address Line 1: ______________________

Address Line 2: ______________________

Address Line 3: ______________________

Post Code: ______________________

[hh1_fname] [hh1_sname] : Has a doctor or other health professional ever told you that you have any of the following conditions?
Please select all that apply.

- ☐ Asthma
- ☐ Arthritis
- ☐ Congestive heart failure
- ☐ Coronary heart disease
- ☐ Angina
- ☐ Heart attack or myocardial infarction
- ☐ Stroke
- ☐ Emphysema
- ☐ Chronic bronchitis
- ☐ COPD (Chronic Obstructive Pulmonary Disease)
- ☐ Cystic fibrosis
- ☐ Hypothyroidism or an under-active thyroid
- ☐ Any kind of liver condition
- ☐ Cancer or malignancy
- ☐ Insulin treated diabetes
- ☐ Other diabetes
- ☐ Epilepsy
- ☐ High blood pressure/hypertension
- ☐ An emotional, nervous or psychiatric problem
- ☐ Multiple Sclerosis
- ☐ HIV
- ☐ Chronic kidney disease
- ☐ Conditions affecting the brain and nerves, such as Parkinson's disease, motor neurone disease, multiple sclerosis (MS), a learning disability or cerebral palsy
- ☐ Problems with your spleen or you've had your spleen removed
- ☐ Sickle cell disease
- ☐ Other long standing/chronic condition
- ☐ None of these

[hh1_fname] [hh1_sname]: Please specify: ______________________

For peer review only

[hh1_fname] [hh1_sname]: What type of cancer or malignancy was that?<br>Please select all that apply	☐ Bowel/colorectal<br>☐ Lung<br>☐ Breast<br>☐ Prostate<br>☐ Liver<br>☐ Skin cancer or melanoma<br>☐ Blood or bone marrow cancer, such as leukaemia<br>☐ Other
[hh1_fname] [hh1_sname]: What type of cancer or malignancy was that?<br>Please select all that apply	☐ Bowel/colorectal<br>☐ Lung<br>☐ Breast<br>☐ Liver<br>☐ Skin cancer or melanoma<br>☐ Blood or bone marrow cancer, such as leukaemia<br>☐ Other
[hh1_fname] [hh1_sname]: Has a doctor or other health professional ever told you that you have any of these conditions?<br>Please select all that apply	☐ Asthma<br>☐ Cystic fibrosis<br>☐ Insulin treated diabetes<br>☐ Epilepsy<br>☐ Conditions affecting the brain and nerves, such as Parkinson's disease, motor neurone disease, multiple sclerosis (MS), a learning disability or cerebral palsy<br>☐ Sickle cell disease<br>☐ Other long standing/chronic condition<br>☐ None of these
[hh1_fname] [hh1_sname] : Have you received a letter from the NHS, saying that "the NHS has identified you as someone at risk of severe illness if you catch coronavirus, because you have an underlying disease or health condition that means if you catch the virus, you are more likely to be admitted to hospital than others"?	◯ Yes<br>◯ No
[hh1_fname] [hh1_sname] : Do you know your height and weight?	◯ Yes<br>◯ No
[hh1_fname] [hh1_sname]: Do you know your height in imperial (feet and inches) or metric (centimetres)?<br>Please select the unit you prefer if you know both	◯ Imperial (Feet and Inches)<br>◯ Metric (centimetres)
[hh1_fname] [hh1_sname]: What is your height in centimetres (cm)?<br>Please enter digits only, e.g. '5' and not 'five'	__________________________
[hh1_fname] [hh1_sname]: How many feet tall are you (rounded down)?<br>Please enter the feet component of your height. For example if you're 5 foot 4, please enter 5	__________________________
[hh1_fname] [hh1_sname]: How many inches tall are you above your feet value?<br>Please enter the inches component of your height. For example if you're 5 foot 4, please enter 4	__________________________

For peer review only

[hh1_fname] [hh1_sname]: Do you know your weight in imperial (stone and pounds-lbs) or metric(kilograms)? Please select the unit you prefer if you know both	◯ Imperial (stone and pounds)<br>◯ Metric (kilograms)
[hh1_fname] [hh1_sname]: How much do you weigh in kilograms (kg)? Please enter digits only, e.g. '5' and not 'five'	______________________
[hh1_fname] [hh1_sname]: What is your weight in stone, rounded down? For example if you are 8 stone, 10 pounds, please enter 8. If you do not use stone, please feel free to leave this blank and enter your weight fully in pounds	______________________
[hh1_fname] [hh1_sname]: How much do you weight in pounds (lbs) (above your stone weight)? For example, if you are 8 stone, 10 pounds, please enter 10.If you did not enter a value for stone, please enter your weight fully in lbs here	______________________

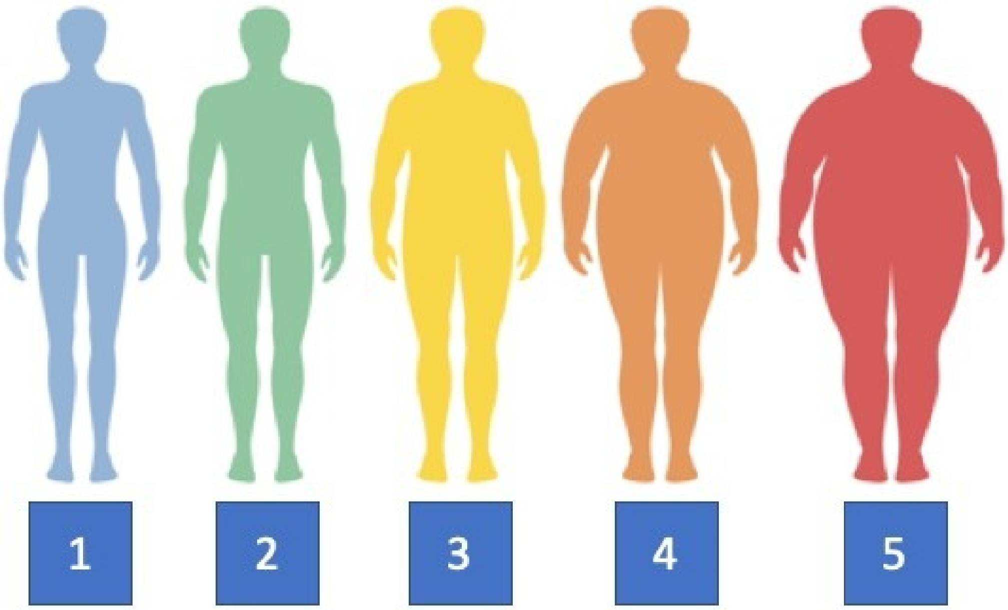

For peer review only - http://bmjopen.bmj.com/site/about/guidelines.xhtml

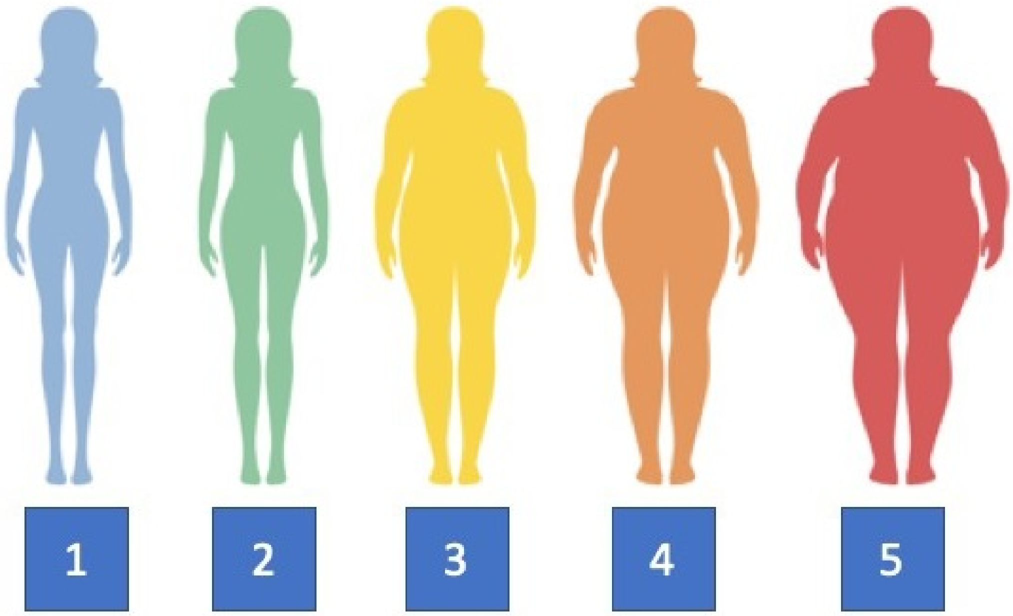

[hh1_fname] [hh1_sname]: Referring to the illustration above, which body shape bests describes your body shape?

- ◯ 1
- ◯ 2
- ◯ 3
- ◯ 4
- ◯ 5

HairSex hormones that affect male pattern baldness may also affect COVID severity

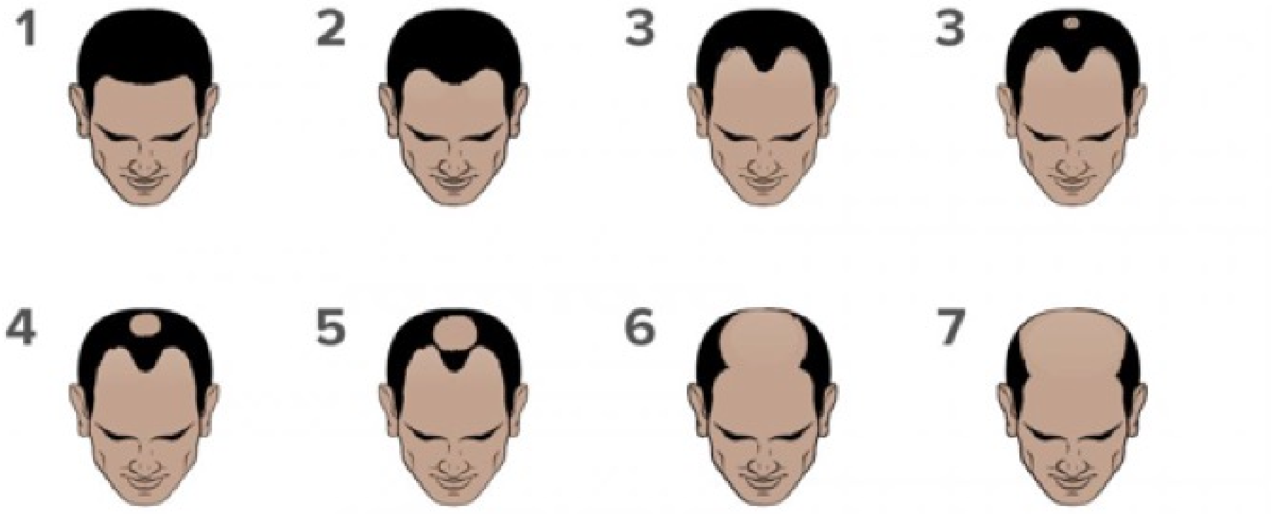

[hh1_fname] [hh1_sname]: Referring to the illustration above, please select a number from 1-7 that best describes your hair	◯ 1<br>◯ 2<br>◯ 3<br>◯ 4<br>◯ 5<br>◯ 6<br>◯ 7

MedicationSome medicines may affect your risk of getting respiratory infections and may either protect against severe diseases or possibly increase the risk. We want to find out more about this

[hh1_fname] [hh1_sname] : Do you take any medication?	◯ Yes<br>◯ No
[hh1_fname] [hh1_sname]: Are you currently receiving treatment or taking medications that may affect your immune system?<br>Please select all that apply	☐ Medication following an organ transplant<br>☐ Medicines such as steroid tablets that weaken the immune system<br>☐ Targeted therapy or chemotherapy for cancer treatment<br>☐ Radiotherapy for cancer treatment<br>☐ Other treatment or medication that may affect immune system<br>☐ None of these
[hh1_fname] [hh1_sname]: Do you regularly take medicine to surpress gastric acid?<br>Please select all that apply	☐ Ranitidine (e.g. Zantac)<br>☐ Omeprazole (e.g. Losec)<br>☐ Antacids (e.g. Rennies)<br>☐ None of these
[hh1_fname] [hh1_sname]: Which of the following medicines do you take?<br>Please select all that apply	☐ Regularly taking Aspirin<br>☐ Regularly taking "NSAIDS" e.g. Ibuprofen, nurofen, diclofenic, naproxen.<br>☐ Regularly taking blood pressure medicines ending in "-pril" such as enalapril, lisinopril, captopril, ramipril<br>☐ Regularly taking blood pressure measurements ending in "-sartan" such as losartan, valsartan, irbesartan<br>☐ Regularly taking anticoagulants e.g warfarin, ivaroxaban (Xarelto), dabigatran (Pradaxa), apixaban (Eliquis), edoxaban (Lixiana)<br>☐ Steroid tablets<br>☐ Regularly use a steroid inhaler<br>☐ Regularly take statins e.g. atorvastatin (Lipitor)<br>☐ None of these
[hh1_fname] [hh1_sname]: Which of the following medicines do you take?<br>Please select all that apply	☐ Steroid tablets<br>☐ Regularly use a steroid inhaler
[hh1_fname] [hh1_sname]: Do you take any vitamin supplements?<br>Please select all that apply	☐ Vitamin C Supplements<br>☐ Vitamin D Supplements<br>☐ Other<br>☐ None
[hh1_fname] [hh1_sname]: Have you ever had a flu vaccine?	◯ Yes<br>◯ No

For peer review only

For peer review only - http://bmjopen.bmj.com/site/about/guidelines.xhtml

[hh1_fname] [hh1_sname]: Approximately when did you have your most recent flu vaccine?
If you do not remember the exact date, please select an approximate date

____

Drinking and Smoking
Drinking and smoking affects the risk of many diseases. We want to find out if it affects the risk of COVID-19 infection

[hh1_fname] [hh1_sname]: Have you ever smoked cigarettes regularly?

- ◯ Yes
- ◯ No

[hh1_fname] [hh1_sname]: And do you smoke cigarettes at all nowadays?

- ◯ Yes
- ◯ No

[hh1_fname] [hh1_sname]: How many cigarettes do you smoke daily?
Please enter digits only, e.g. '5' and not 'five'

____

[hh1_fname] [hh1_sname]: When did you give up smoking?

- ◯ Less than 3 months ago
- ◯ 3 - 6 months ago
- ◯ More than 6 months ago but less than 1 year ago
- ◯ 1 year or more ago

[hh1_fname] [hh1_sname]: Thinking about the past month, how often did you have a drink containing alcohol?

- ◯ Daily
- ◯ 4-6 Times per week
- ◯ 2-3 Times per week
- ◯ Weekly or Less
- ◯ 2-4 times per month
- ◯ Never

[hh1_fname] [hh1_sname]: How many drinks do you have on a typical day when you are drinking?

- ◯ 1-2 Drinks
- ◯ 3-4 Drinks
- ◯ 5-6 Drinks
- ◯ 7-9 Drinks
- ◯ 10+

Accessing Health care during the lockdown

[hh1_fname] [hh1_sname]: Have you had any healthcare appointments cancelled, postponed or changed to a telephone or online (including video) consultation since the start of the pandemic?

- ◯ Yes
- ◯ No

For peer review only

Please tick all that applied

- ☐ I had an operation cancelled or postponed
- ☐ I had a planned hospital admission cancelled or postponed
- ☐ I had a hospital outpatient appointment cancelled or postponed
- ☐ I had a hospital outpatient appointment changed to a phone call or online (including video) consultation
- ☐ I had a GP appointment cancelled or postponed
- ☐ I had a GP appointment changed to a phone call or online (including video) consultation
- ☐ I had another NHS appointment cancelled or postponed
- ☐ I had another NHS appointment changed to a phone call or online (including video)

For peer review only

Employment status for [hh1_fname] [hh1_sname]

Many people's work has been affected by the coronavirus and people's work can affect their chance of catching coronavirus. We'd like to know about your work and how it has been effected by the coronavirus.

[hh1_fname] [hh1_sname]: Thinking back to earlier this year, before the outbreak of the coronavirus pandemic. Which of these description best describes your work status?

- ◯ Employed full time
- ◯ Employed part time
- ◯ Self employed full time
- ◯ Self employed part time
- ◯ Retired
- ◯ Student
- ◯ Looking after house/family (not looking for work)
- ◯ Permanently sick or disabled
- ◯ Unemployed
- ◯ None of the above

[hh1_fname] [hh1_sname]: What is/was the name of your job?

____

[hh1_fname] [hh1_sname]: Please describe what you do/did at work

____

[hh1_fname] [hh1_sname]: Are you a health or social care worker?

- ◯ Yes
- ◯ No

What setting do you work in?

- ◯ Secondary Care
- ◯ Accident and emergency
- ◯ Primary Care
- ◯ Care home (residential or nursing)
- ◯ Community
- ◯ Other (specify)

Other (Please Specify)

____

[hh1_fname] [hh1_sname]: Please select your healthcare profession

- ◯ Doctor
- ◯ Nurse
- ◯ Profession allied to medicine (e.g. occupational therapy, physiotherapy, podiatry)
- ◯ Psychological Professions
- ◯ Pharmacy
- ◯ Midwifery
- ◯ Healthcare science (e.g. laboratory, radiology)
- ◯ Management
- ◯ Porter
- ◯ Cleaner
- ◯ Administrative Staff with regular patient contact
- ◯ Administrative Staff with minimal or no patient contact
- ◯ Care Worker
- ◯ Personal Assistant
- ◯ Social Worker
- ◯ Community support and outreach worker
- ◯ Other

[hh1_fname] [hh1_sname]: Please specify

____

For peer review only

[hh1_fname] [hh1_sname]: What was your last job when you were working?

____

[hh1_fname] [hh1_sname]: Are you a healthcare student? e.g. medical student, student nurse

◯ Yes
◯ No

Employment during the pandemic

[hh1_fname] [hh1_sname]: BEFORE THE PANDEMIC, how many hours of paid work did you work per week?
Please enter digits only, e.g. '5' and not 'five'

____

[hh1_fname] [hh1_sname]: SINCE THE PANDEMIC, during the months of March, April, May, how many hours of paid work have you worked since each week?
Please enter digits only, e.g. '5' and not 'five'

____

It looks like you have reduced your paid work during the lockdown.

[hh1_fname] [hh1_sname] : Why have you had to reduce the number of hours?

◯ Laid off by employer with certain recall date
◯ Laid off or made redundant by employer with some prospect of recall
◯ Employer cut hours
◯ Have been put on furlough or paid leave
◯ Using annual leave
◯ On paid or statutory sick leave
◯ On unpaid sick leave
◯ Caring for children or others
◯ Other reasons

[hh1_fname] [hh1_sname] : Why have you had to reduce the number of hours?

◯ My business has been directly affected by regulations on opening or other new regulations
◯ My business has been directly affected by limited supplies that I need for my business
◯ My business has been directly affected by reduced demand for my services or products
◯ Illness
◯ Self-isolating
◯ Caring for children or others
◯ Other reasons

[hh1_fname] [hh1_sname]: Have you received a written letter from your employer informing you that you have been furloughed under the Coronavirus Job Retention scheme?

◯ Yes
◯ No

Working from home during the pandemic

[hh1_fname] [hh1_sname]: How often did you WORK FROM HOME during the following three time periods:

	(Nearly) Always	Often	Sometimes	(Almost) Never
Before the lockdown (before March 2020)	◯	◯	◯	◯

For peer review only

During the main lockdown (Mid March to Mid May) when we were asked to stay at Home, Save Lives, Support the NHS	○	○	○	○
After the easing of restrictions in mid-May, when we were encouraged to go back to work if we could not work from home (Stay Alert, Control the Virus, Save Lives)	○	○	○	○

[hh1_fname] [hh1_sname]: How do/did you usually travel to work?
Please choose one or more to represent a typical day

	Before the lockdown (before March 2020)	During the main lockdown (Mid March to Mid May) when we were asked to stay at Home, Save Lives, Support the NHS	After the easing of restrictions in mid-May, when we were encouraged to go back to work if we could not work from home (Stay Alert, Control the Virus, Save Lives)
By car or van	☐	☐	☐
Motorcycle, moped or scooter	☐	☐	☐
Taxi or minicab	☐	☐	☐
Train	☐	☐	☐
Underground	☐	☐	☐
Bus	☐	☐	☐
Tram or light railway	☐	☐	☐
Cycle	☐	☐	☐
Walk	☐	☐	☐
Other	☐	☐	☐
I did not travel to work during this period	☐	☐	☐

[hh1_fname] [hh1_sname]: BEFORE LOCKDOWN, how long did it take to get to work each day?
Please provide the time for a one-way journey in minutes or enter 0 if you did not travel to work in this period

____

[hh1_fname] [hh1_sname]: DURING LOCKDOWN, how long did it take to get to work each day?
Please provide the time for a one-way journey in minutes or enter 0 if you did not travel to work in this period

____

For peer review only

[hh1_fname] [hh1_sname]: AFTER THE EASING OF RESTRICTIONS, how long did it take to get to work each day?
Please provide the time for a one-way journey in minutes or enter 0 if you did not travel to work in this period

____

For peer review only

Finances details for [hh1_fname] [hh1_sname]

Many people have been affected financially by the coronavirus. We'd like to know how you have been affected and how you and your household are coping.

[hh1_fname] [hh1_sname]: What is your combined household income last year?<br>We want to understand how COVID-19 impacts households with different levels of income	◯ 0-9,999<br>◯ 10,000- 24,999<br>◯ 25,000 - 49,999<br>◯ 50,000 - 74,999<br>◯ 75,000 - 99,999<br>◯ 100,000 - 124,999<br>◯ 125,000 - 149,999<br>◯ 150,000 - 174,999<br>◯ 175,000 - 199,999<br>◯ 200,000 or more<br>◯ Prefer not to say
[hh1_fname] [hh1_sname] : BEFORE THE PANDEMIC, how would you say you were managing financially? Would you say you were:	◯ Living comfortably<br>◯ Doing alright<br>◯ Just about getting by<br>◯ Finding it quite difficult<br>◯ Finding it very difficult
[hh1_fname] [hh1_sname]: How would you say you are managing financially now? Would you say you were:	◯ Living comfortably<br>◯ Doing alright<br>◯ Just about getting by<br>◯ Finding it quite difficult<br>◯ Finding it very difficult
[hh1_fname] [hh1_sname]: BEFORE THE PANDEMIC, have you ever needed to use a food bank?	◯ Never<br>◯ Less than once a week<br>◯ Once a week or more
[hh1_fname] [hh1_sname]: SINCE THE PANDEMIC, have you needed to use a food bank?	◯ Never<br>◯ Less than once a week<br>◯ Once a week or more

For peer review only

Caring information during the lockdown - [hh1_fname] [hh1_sname]

[hh1_fname] [hh1_sname]: About how many hours a week did you spend on childcare or home-schooling during the lockdown?
Please enter digits only, e.g. '5' and not 'five'

____

[hh1_fname] [hh1_sname]: Who is mainly responsible for looking after the children or home schooling?

- ◯ Mainly you
- ◯ Mainly your husband/wife/partner
- ◯ Jointly with your husband/wife/partner
- ◯ Someone else

For peer review only

Previous COVID-19 like illness for [hh1_fname] [hh1_sname]

[hh1_fname] [hh1_sname]: Have you EVER come into contact with anyone that was known or presumed to have COVID-19?
Close contact includes:
Physical contact with another personA five minute conversation with someone less than 2 metres awayBeing less than 2 metres away from someone for 15 minutes or more, even if you didn't talk to each otherPlease select all that apply.

- ☐ No
- ☐ Yes (a household member)
- ☐ Yes (at work)
- ☐ Yes (a non-household friend or relation)
- ☐ Yes (in public)
- ☐ Yes (other)
- ☐ Don't know

Did this person have COVID confirmed by a laboratory test?

- ◯ Yes
- ◯ No
- ◯ Don't know

[hh1_fname] [hh1_sname]: Have you had an illness involving Cough, or Fever, or Loss of sense of smell since the 1st of January 2020?

- ◯ Yes
- ◯ No

[hh1_fname] [hh1_sname]: Which month did the illness start and which symptoms did you have?
If you had more than one illness, you may select multiple start months

	Jan 2020	Feb 2020	Mar 2020	Apr 2020	May 2020	Jun 2020	Jul 2020	Aug 2020	Sep 2020	Oct 2020	Nov 2020	Dec 2020	Jan 2021	Feb 2021
Cough	☐	☐	☐	☐	☐	☐	☐	☐	☐	☐	☐	☐	☐	☐
Fever	☐	☐	☐	☐	☐	☐	☐	☐	☐	☐	☐	☐	☐	☐
Loss of sense of smell	☐	☐	☐	☐	☐	☐	☐	☐	☐	☐	☐	☐	☐	☐

[hh1_fname] [hh1_sname]: COVID-19 can cause a wide range of symptoms. Have you had an illness that you think might have been COVID-19, or which was confirmed as COVID-19?

- ◯ Yes
- ◯ No

Please describe in your own words how did the illness started and what you did when you started to feel ill?

____

Please describe in your own words, what symptoms developed and what did you do?

____

Please describe in your own words, how long did your symptoms last and are any symptoms still there?

____

[hh1_fname] [hh1_sname]: Have you ever had a nose or throat swab test for COVID-19?

- ◯ Yes
- ◯ No
- ◯ Not Sure

What month was the swab taken and what was the result?
Please select all the months in which you took each test (if you took multiple) and their results

For peer review only

	The test was positive	The test was negative	The test was unclear	I haven't had the result
Jan 2020	◯	◯	◯	◯
Feb 2020	◯	◯	◯	◯
Mar 2020	◯	◯	◯	◯
Apr 2020	◯	◯	◯	◯
May 2020	◯	◯	◯	◯
Jun 2020	◯	◯	◯	◯
Jul 2020	◯	◯	◯	◯
Aug 2020	◯	◯	◯	◯
Sep 2020	◯	◯	◯	◯
Oct 2020	◯	◯	◯	◯
Nov 2020	◯	◯	◯	◯
Dec 2020	◯	◯	◯	◯
Jan 2021	◯	◯	◯	◯
Feb 2021	◯	◯	◯	◯

For peer review only

Thank You

We want to know what questions you think are important so we can try to answer them with the help of tens of thousands of Virus Watch participants. Each month we will ask our participants to say what questions they would like to see answered.

[hh1_fname] [hh1_sname]: What questions would you like Virus Watch to answer?

____

As we come out of a very difficult and tragic period we want to know about your three main worries related to COVID-19 and the COVID-19 response.

[hh1_fname] [hh1_sname]: What are your three main worries about the COVID-19 pandemic?

1st most worrying aspect: __________________________________

2nd most worrying aspect: __________________________________

3rd most worrying aspect: __________________________________

Please click submit to continue

For peer review only

Weekly Survey

Welcome to the weekly followup survey

surveydate ______________________

In Virus Watch we are interested in the following types of symptoms:
General symptoms (e.g. fevers, general muscle aches, headache, joint pain,extreme tiredness, trouble with daily activities around the house)Respiratory Symptoms (e.g. cough, shortness of breath, earache, sore throat, runny nose, blocked nose, sneezing, wheeze, loss or altered senses of smell or taste).Eyes (e.g. eye redness, eye pain,sticky eye, deterioration of eyesight)RashesDigestive symptoms (e.g. diarrhoea or loose stools, vomiting, nausea, abdominal pain)This survey is about symptoms in the week (Monday to Sunday) before you received the email with the survey link.

Download Symptom Diary

[Attachment: "Virus_Watch_Symptom_Diary.pdf"]

Have you or anyone in the household had any of these symptoms in the past week?

Please continue to report weekly symptoms even if you believe them to be related to a recent vaccine you have had.

◯ Yes
◯ No

Did any household members receive a COVID-19 test result in the past week?

◯ Yes
◯ No

Has anyone in the household been advised to self-isolate in the past week?

◯ Yes
◯ No

Please indicate who received a result for a COVID-19 (swab or lateral flow) test in the past week: If you have had multiple tests this week please report any positive test dates OR your last negative test dates (if all were negative)

[go_arm_1][hh1_fname] [go_arm_1][hh1_sname] ☐

[go_arm_1][hh1_fname] [go_arm_1][hh1_sname]: What was the result of the COVID-19 test?

◯ Positive
◯ Negative
◯ Unclear

[go_arm_1][hh1_fname] [go_arm_1][hh1_sname]: When was the test taken? ______________________

[go_arm_1][hh1_fname] [go_arm_1][hh1_sname]: When did you receive the test result? ______________________

[go_arm_1][hh2_fname] [go_arm_1][hh2_sname] ☐

[go_arm_1][hh2_fname] [go_arm_1][hh2_sname]: What was the result of the COVID-19 test?

◯ Positive
◯ Negative
◯ Unclear

[go_arm_1][hh2_fname] [go_arm_1][hh2_sname]: When was the test taken? ______________________

For peer review only

[go_arm_1][hh2_fname] [go_arm_1][hh2_sname]: When did you receive the test result?	______________________________
[go_arm_1][hh3_fname] [go_arm_1][hh3_sname]	☐
[go_arm_1][hh3_fname] [go_arm_1][hh3_sname]: What was the result of the COVID-19 test?	◯ Positive<br>◯ Negative<br>◯ Unclear
[go_arm_1][hh3_fname] [go_arm_1][hh3_sname]: When was the test taken?	______________________________
[go_arm_1][hh3_fname] [go_arm_1][hh3_sname]: When did you receive the test result?	______________________________
[go_arm_1][hh4_fname] [go_arm_1][hh4_sname]	☐
[go_arm_1][hh4_fname] [go_arm_1][hh4_sname]: What was the result of the COVID-19 test?	◯ Positive<br>◯ Negative<br>◯ Unclear
[go_arm_1][hh4_fname] [go_arm_1][hh4_sname]: When was the test taken?	______________________________
[go_arm_1][hh4_fname] [go_arm_1][hh4_sname]: When did you receive the test result?	______________________________
[go_arm_1][hh5_fname] [go_arm_1][hh5_sname]	☐
[go_arm_1][hh5_fname] [go_arm_1][hh5_sname]: What was the result of the COVID-19 test?	◯ Positive<br>◯ Negative<br>◯ Unclear
[go_arm_1][hh5_fname] [go_arm_1][hh5_sname]: When was the test taken?	______________________________
[go_arm_1][hh5_fname] [go_arm_1][hh5_sname]: When did you receive the test result?	______________________________
[go_arm_1][hh6_fname] [go_arm_1][hh6_sname]	☐
[go_arm_1][hh6_fname] [go_arm_1][hh6_sname]: What was the result of the COVID-19 test?	◯ Positive<br>◯ Negative<br>◯ Unclear
[go_arm_1][hh6_fname] [go_arm_1][hh6_sname]: When was the test taken?	______________________________
[go_arm_1][hh6_fname] [go_arm_1][hh6_sname]: When did you receive the test result?	______________________________
Please indicate who was advised to self isolate in the past week:	
[go_arm_1][hh1_fname] [go_arm_1][hh1_sname]	☐

For peer review only

[go_arm_1][hh1_fname] [go_arm_1][hh1_sname]: Which of the following led to the request to self-isolate?	◯ The Test and Trace programme advised me to self-isolate because I have symptoms of COVID-19<br>◯ The Test and Trace programme advised me to self-isolate because I am had a positive test result<br>◯ The Test and Trace programme advised me to self isolate because I was in contact with a COVID-19 case<br>◯ The NHS COVID-19 app alerted me that I had been in contact with a COVID-19 case<br>◯ I have returned from a country where quarantine is advised after return.<br>◯ Other reason
[go_arm_1][hh2_fname] [go_arm_1][hh2_sname]	☐
[go_arm_1][hh2_fname] [go_arm_1][hh2_sname]: Which of the following led to the request to self-isolate?	◯ The Test and Trace programme advised me to self-isolate because I have symptoms of COVID-19<br>◯ The Test and Trace programme advised me to self-isolate because I am had a positive test result<br>◯ The Test and Trace programme advised me to self isolate because I was in contact with a COVID-19 case<br>◯ The NHS COVID-19 app alerted me that I had been in contact with a COVID-19 case<br>◯ I have returned from a country where quarantine is advised after return.<br>◯ Other reason
[go_arm_1][hh3_fname] [go_arm_1][hh3_sname]	☐
[go_arm_1][hh3_fname] [go_arm_1][hh3_sname]: Which of the following led to the request to self-isolate?	◯ The Test and Trace programme advised me to self-isolate because I have symptoms of COVID-19<br>◯ The Test and Trace programme advised me to self-isolate because I am had a positive test result<br>◯ The Test and Trace programme advised me to self isolate because I was in contact with a COVID-19 case<br>◯ The NHS COVID-19 app alerted me that I had been in contact with a COVID-19 case<br>◯ I have returned from a country where quarantine is advised after return.<br>◯ Other reason
[go_arm_1][hh4_fname] [go_arm_1][hh4_sname]	☐

For peer review only

[go_arm_1][hh4_fname] [go_arm_1][hh4_sname]: Which of the following led to the request to self-isolate?	◯ The Test and Trace programme advised me to self-isolate because I have symptoms of COVID-19<br>◯ The Test and Trace programme advised me to self-isolate because I am had a positive test result<br>◯ The Test and Trace programme advised me to self isolate because I was in contact with a COVID-19 case<br>◯ The NHS COVID-19 app alerted me that I had been in contact with a COVID-19 case<br>◯ I have returned from a country where quarantine is advised after return.<br>◯ Other reason
[go_arm_1][hh5_fname] [go_arm_1][hh5_sname]	☐
[go_arm_1][hh5_fname] [go_arm_1][hh5_sname]: Which of the following led to the request to self-isolate?	◯ The Test and Trace programme advised me to self-isolate because I have symptoms of COVID-19<br>◯ The Test and Trace programme advised me to self-isolate because I am had a positive test result<br>◯ The Test and Trace programme advised me to self isolate because I was in contact with a COVID-19 case<br>◯ The NHS COVID-19 app alerted me that I had been in contact with a COVID-19 case<br>◯ I have returned from a country where quarantine is advised after return.<br>◯ Other reason
[go_arm_1][hh6_fname] [go_arm_1][hh6_sname]	☐
[go_arm_1][hh6_fname] [go_arm_1][hh6_sname]: Which of the following led to the request to self-isolate?	◯ The Test and Trace programme advised me to self-isolate because I have symptoms of COVID-19<br>◯ The Test and Trace programme advised me to self-isolate because I am had a positive test result<br>◯ The Test and Trace programme advised me to self isolate because I was in contact with a COVID-19 case<br>◯ The NHS COVID-19 app alerted me that I had been in contact with a COVID-19 case<br>◯ I have returned from a country where quarantine is advised after return.<br>◯ Other reason
Please indicate who has had symptoms in the past week:	
[go_arm_1][hh1_fname] [go_arm_1][hh1_sname]:	◯ Yes, symptoms that have been present for less than 2 months<br>◯ Yes, symptoms that developed after a COVID-19 like illness and have lasted for more than 2 months<br>◯ Yes, symptoms that are part of a long term chronic illness<br>◯ No symptoms

For peer review only

[go_arm_1][hh2_fname] [go_arm_1][hh2_sname]:	◯ Yes, symptoms that have been present for less than 2 months<br>◯ Yes, symptoms that developed after a COVID-19 like illness and have lasted for more than 2 months<br>◯ Yes, symptoms that are part of a long term chronic illness<br>◯ No symptoms
[go_arm_1][hh3_fname] [go_arm_1][hh3_sname]:	◯ Yes, symptoms that have been present for less than 2 months<br>◯ Yes, symptoms that developed after a COVID-19 like illness and have lasted for more than 2 months<br>◯ Yes, symptoms that are part of a long term chronic illness<br>◯ No symptoms
[go_arm_1][hh4_fname] [go_arm_1][hh4_sname]:	◯ Yes, symptoms that have been present for less than 2 months<br>◯ Yes, symptoms that developed after a COVID-19 like illness and have lasted for more than 2 months<br>◯ Yes, symptoms that are part of a long term chronic illness<br>◯ No symptoms
[go_arm_1][hh5_fname] [go_arm_1][hh5_sname]:	◯ Yes, symptoms that have been present for less than 2 months<br>◯ Yes, symptoms that developed after a COVID-19 like illness and have lasted for more than 2 months<br>◯ Yes, symptoms that are part of a long term chronic illness<br>◯ No symptoms
[go_arm_1][hh6_fname] [go_arm_1][hh6_sname]:	◯ Yes, symptoms that have been present for less than 2 months<br>◯ Yes, symptoms that developed after a COVID-19 like illness and have lasted for more than 2 months<br>◯ Yes, symptoms that are part of a long term chronic illness<br>◯ No symptoms

For peer review only

Please indicate whether the following members have received a result for a COVID-19 (swab or lateral flow) test in the past week: If you have had multiple tests this week please report any positive test dates OR your last negative test dates (if all were negative)

[go_arm_1][hh1_fname] [go_arm_1][hh1_sname]:	☐
[go_arm_1][hh1_fname] [go_arm_1][hh1_sname]: What was the result of this test?	◯ Positive<br>◯ Negative<br>◯ Unclear
[go_arm_1][hh1_fname] [go_arm_1][hh1_sname]: When was the test taken?	__________________________________
[go_arm_1][hh1_fname] [go_arm_1][hh1_sname]: When did you receive the test result?	__________________________________
[go_arm_1][hh2_fname] [go_arm_1][hh2_sname]:	☐

[go_arm_1][hh2_fname] [go_arm_1][hh2_sname]: What was the result of this test?	◯ Positive<br>◯ Negative<br>◯ Unclear
[go_arm_1][hh2_fname] [go_arm_1][hh2_sname]: When was the test taken?	__________________
[go_arm_1][hh2_fname] [go_arm_1][hh2_sname]: When did you receive the test result?	__________________
[go_arm_1][hh3_fname] [go_arm_1][hh3_sname]:	☐
[go_arm_1][hh3_fname] [go_arm_1][hh3_sname]: What was the result of this test?	◯ Positive<br>◯ Negative<br>◯ Unclear
[go_arm_1][hh3_fname] [go_arm_1][hh3_sname]: When was the test taken?	__________________
[go_arm_1][hh3_fname] [go_arm_1][hh3_sname]: When did you receive the test result?	__________________
[go_arm_1][hh4_fname] [go_arm_1][hh4_sname]:	☐
[go_arm_1][hh4_fname] [go_arm_1][hh4_sname]: What was the result of this test?	◯ Positive<br>◯ Negative<br>◯ Unclear
[go_arm_1][hh4_fname] [go_arm_1][hh4_sname]: When was the test taken?	__________________
[go_arm_1][hh4_fname] [go_arm_1][hh4_sname]: When did you receive the test result?	__________________
[go_arm_1][hh5_fname] [go_arm_1][hh5_sname]:	☐
[go_arm_1][hh5_fname] [go_arm_1][hh5_sname]: What was the result of this test?	◯ Positive<br>◯ Negative<br>◯ Unclear
[go_arm_1][hh5_fname] [go_arm_1][hh5_sname]: When was the test taken?	__________________
[go_arm_1][hh5_fname] [go_arm_1][hh5_sname]: When did you receive the test result?	__________________
[go_arm_1][hh6_fname] [go_arm_1][hh6_sname]:	☐
[go_arm_1][hh6_fname] [go_arm_1][hh6_sname]: What was the result of this test?	◯ Positive<br>◯ Negative<br>◯ Unclear
[go_arm_1][hh6_fname] [go_arm_1][hh6_sname]: When was the test taken?	__________________

For peer review only

[go_arm_1][hh6_fname] [go_arm_1][hh6_sname]: When did you receive the test result?	______________________________
Please indiciate whether the following household members been advised to self-isolate in the past week:	
[go_arm_1][hh1_fname] [go_arm_1][hh1_sname]	☐
[go_arm_1][hh1_fname] [go_arm_1][hh1_sname]: Which of the following led to the request to self-isolate?	◯ The Test and Trace programme advised me to self-isolate because I have symptoms of COVID-19<br>◯ The Test and Trace programme advised me to self-isolate because I am had a positive test result<br>◯ The Test and Trace programme advised me to self isolate because I was in contact with a COVID-19 case<br>◯ The NHS COVID-19 app alerted me that I had been in contact with a COVID-19 case<br>◯ I have returned from a country where quarantine is advised after return.<br>◯ Other reason
[go_arm_1][hh2_fname] [go_arm_1][hh2_sname]	☐
[go_arm_1][hh2_fname] [go_arm_1][hh2_sname]: Which of the following led to the request to self-isolate?	◯ The Test and Trace programme advised me to self-isolate because I have symptoms of COVID-19<br>◯ The Test and Trace programme advised me to self-isolate because I am had a positive test result<br>◯ The Test and Trace programme advised me to self isolate because I was in contact with a COVID-19 case<br>◯ The NHS COVID-19 app alerted me that I had been in contact with a COVID-19 case<br>◯ I have returned from a country where quarantine is advised after return.<br>◯ Other reason
[go_arm_1][hh3_fname] [go_arm_1][hh3_sname]	☐
[go_arm_1][hh3_fname] [go_arm_1][hh3_sname]: Which of the following led to the request to self-isolate?	◯ The Test and Trace programme advised me to self-isolate because I have symptoms of COVID-19<br>◯ The Test and Trace programme advised me to self-isolate because I am had a positive test result<br>◯ The Test and Trace programme advised me to self isolate because I was in contact with a COVID-19 case<br>◯ The NHS COVID-19 app alerted me that I had been in contact with a COVID-19 case<br>◯ I have returned from a country where quarantine is advised after return.<br>◯ Other reason
[go_arm_1][hh4_fname] [go_arm_1][hh4_sname]	☐

For peer review only

[go_arm_1][hh4_fname] [go_arm_1][hh4_sname]: Which of the following led to the request to self-isolate?	◯ The Test and Trace programme advised me to self-isolate because I have symptoms of COVID-19<br>◯ The Test and Trace programme advised me to self-isolate because I am had a positive test result<br>◯ The Test and Trace programme advised me to self isolate because I was in contact with a COVID-19 case<br>◯ The NHS COVID-19 app alerted me that I had been in contact with a COVID-19 case<br>◯ I have returned from a country where quarantine is advised after return.<br>◯ Other reason
[go_arm_1][hh5_fname] [go_arm_1][hh5_sname]	☐
[go_arm_1][hh5_fname] [go_arm_1][hh5_sname]: Which of the following led to the request to self-isolate?	◯ The Test and Trace programme advised me to self-isolate because I have symptoms of COVID-19<br>◯ The Test and Trace programme advised me to self-isolate because I am had a positive test result<br>◯ The Test and Trace programme advised me to self isolate because I was in contact with a COVID-19 case<br>◯ The NHS COVID-19 app alerted me that I had been in contact with a COVID-19 case<br>◯ I have returned from a country where quarantine is advised after return.<br>◯ Other reason
[go_arm_1][hh6_fname] [go_arm_1][hh6_sname]	☐
[go_arm_1][hh6_fname] [go_arm_1][hh6_sname]: Which of the following led to the request to self-isolate?	◯ The Test and Trace programme advised me to self-isolate because I have symptoms of COVID-19<br>◯ The Test and Trace programme advised me to self-isolate because I am had a positive test result<br>◯ The Test and Trace programme advised me to self isolate because I was in contact with a COVID-19 case<br>◯ The NHS COVID-19 app alerted me that I had been in contact with a COVID-19 case<br>◯ I have returned from a country where quarantine is advised after return.<br>◯ Other reason

For peer review only

Vaccination

Has anyone in the household received a COVID-19 vaccine in the past week?	◯ Yes<br>◯ No<br>◯ Unsure (e.g. as part of a blinded COVID-19 trial)
Please indicate who received a COVID-19 vaccine:	
[go_arm_1][hh1_fname] [go_arm_1][hh1_sname]	☐
[go_arm_1][hh1_fname] [go_arm_1][hh1_sname]: Please select which dose(s) of the COVID-19 vaccine you received?	☐ 1st Dose<br>☐ 2nd Dose
[go_arm_1][hh1_fname] [go_arm_1][hh1_sname]: Which type of vaccine did you receive as the 1st dose?	◯ Pfizer Biontech vaccine<br>◯ Oxford AstraZeneca vaccine<br>◯ Other vaccine<br>◯ Don't know/Don't remember
[go_arm_1][hh1_fname] [go_arm_1][hh1_sname]: What date did you receive the 1st dose?<br>(dd-mm-yyyy)<br><br>Please provide an estimate if you cannot recall the date	______________________________
[go_arm_1][hh1_fname] [go_arm_1][hh1_sname]: Which type of vaccine did you receive as the 2nd dose?	◯ Pfizer Biontech vaccine<br>◯ Oxford AstraZeneca vaccine<br>◯ Other vaccine<br>◯ Don't know/Don't remember
[go_arm_1][hh1_fname] [go_arm_1][hh1_sname]: What date did you receive the 2nd dose?<br>(dd-mm-yyyy)<br><br>Please provide an estimate if you cannot recall the date	______________________________
[go_arm_1][hh2_fname] [go_arm_1][hh2_sname]	☐
[go_arm_1][hh2_fname] [go_arm_1][hh2_sname]: Please select which dose(s) of the COVID-19 vaccine you received?	☐ 1st Dose<br>☐ 2nd Dose
[go_arm_1][hh2_fname] [go_arm_1][hh2_sname]: Which type of vaccine did you receive as the 1st dose?	◯ Pfizer Biontech vaccine<br>◯ Oxford AstraZeneca vaccine<br>◯ Other vaccine<br>◯ Don't know/Don't remember
[go_arm_1][hh2_fname] [go_arm_1][hh2_sname]: What date did you receive the 1st dose? (dd-mm-yyyy)<br><br>Please provide an estimate if you cannot recall the date	______________________________

For peer review only

[go_arm_1][hh2_fname] [go_arm_1][hh2_sname]: Which type of vaccine did you receive as the 2nd dose?	◯ Pfizer Biontech vaccine<br>◯ Oxford AstraZeneca vaccine<br>◯ Other vaccine<br>◯ Don't know/Don't remember
[go_arm_1][hh2_fname] [go_arm_1][hh2_sname]: What date did you receive the 2nd dose? (dd-mm-yyyy)<br><br>Please provide an estimate if you cannot recall the date	______________________
[go_arm_1][hh3_fname] [go_arm_1][hh3_sname]	☐
[go_arm_1][hh3_fname] [go_arm_1][hh3_sname]: Please select which dose(s) of the COVID-19 vaccine you received?	☐ 1st Dose<br>☐ 2nd Dose
[go_arm_1][hh3_fname] [go_arm_1][hh3_sname]: Which type of vaccine did you receive as the 1st dose?	◯ Pfizer Biontech vaccine<br>◯ Oxford AstraZeneca vaccine<br>◯ Other vaccine<br>◯ Don't know/Don't remember
[go_arm_1][hh3_fname] [go_arm_1][hh3_sname]: What date did you receive the 1st dose? (dd-mm-yyyy)<br><br>Please provide an estimate if you cannot recall the date	______________________
[go_arm_1][hh3_fname] [go_arm_1][hh3_sname]: Which type of vaccine did you receive as the 2nd dose?	◯ Pfizer Biontech vaccine<br>◯ Oxford AstraZeneca vaccine<br>◯ Other vaccine<br>◯ Don't know/Don't remember
[go_arm_1][hh3_fname] [go_arm_1][hh3_sname]: What date did you receive the 2nd dose? (dd-mm-yyyy)<br><br>Please provide an estimate if you cannot recall the date	______________________
[go_arm_1][hh4_fname] [go_arm_1][hh4_sname]	☐
[go_arm_1][hh4_fname] [go_arm_1][hh4_sname]: Please select which dose(s) of the COVID-19 vaccine you received?	☐ 1st Dose<br>☐ 2nd Dose
[go_arm_1][hh4_fname] [go_arm_1][hh4_sname]: Which type of vaccine did you receive as the 1st dose?	◯ Pfizer Biontech vaccine<br>◯ Oxford AstraZeneca vaccine<br>◯ Other vaccine<br>◯ Don't know/Don't remember
[go_arm_1][hh4_fname] [go_arm_1][hh4_sname]: What date did you receive the 1st dose? (dd-mm-yyyy)<br><br>Please provide an estimate if you cannot recall the date	______________________

For peer review only

[go_arm_1][hh4_fname] [go_arm_1][hh4_sname]: Which type of vaccine did you receive as the 2nd dose?	◯ Pfizer Biontech vaccine<br>◯ Oxford AstraZeneca vaccine<br>◯ Other vaccine<br>◯ Don't know/Don't remember
[go_arm_1][hh4_fname] [go_arm_1][hh4_sname]: What date did you receive the 2nd dose? (dd-mm-yyyy)<br><br>Please provide an estimate if you cannot recall the date	______________________
[go_arm_1][hh5_fname] [go_arm_1][hh5_sname]	☐
[go_arm_1][hh5_fname] [go_arm_1][hh5_sname]: Please select which dose(s) of the COVID-19 vaccine you received?	☐ 1st Dose<br>☐ 2nd Dose
[go_arm_1][hh5_fname] [go_arm_1][hh5_sname]: Which type of vaccine did you receive as the 1st dose?	◯ Pfizer Biontech vaccine<br>◯ Oxford AstraZeneca vaccine<br>◯ Other vaccine<br>◯ Don't know/Don't remember
[go_arm_1][hh5_fname] [go_arm_1][hh5_sname]: What date did you receive the 1st dose? (dd-mm-yyyy)<br><br>Please provide an estimate if you cannot recall the date	______________________
[go_arm_1][hh5_fname] [go_arm_1][hh5_sname]: Which type of vaccine did you receive as the 2nd dose?	◯ Pfizer Biontech vaccine<br>◯ Oxford AstraZeneca vaccine<br>◯ Other vaccine<br>◯ Don't know/Don't remember
[go_arm_1][hh5_fname] [go_arm_1][hh5_sname]: What date did you receive the 2nd dose? (dd-mm-yyyy)<br><br>Please provide an estimate if you cannot recall the date	______________________
[go_arm_1][hh6_fname] [go_arm_1][hh6_sname]	☐
[go_arm_1][hh6_fname] [go_arm_1][hh6_sname]: Please select which dose(s) of the COVID-19 vaccine you received?	☐ 1st Dose<br>☐ 2nd Dose
[go_arm_1][hh6_fname] [go_arm_1][hh6_sname]: Which type of vaccine did you receive as the 1st dose?	◯ Pfizer Biontech vaccine<br>◯ Oxford AstraZeneca vaccine<br>◯ Other vaccine<br>◯ Don't know/Don't remember
[go_arm_1][hh6_fname] [go_arm_1][hh6_sname]: What date did you receive the 1st dose? (dd-mm-yyyy)<br><br>Please provide an estimate if you cannot recall the date	______________________

For peer review only

[go_arm_1][hh6_fname] [go_arm_1][hh6_sname]: Which type of vaccine did you receive as the 2nd dose?	◯ Pfizer Biontech vaccine<br>◯ Oxford AstraZeneca vaccine<br>◯ Other vaccine<br>◯ Don't know/Don't remember
[go_arm_1][hh6_fname] [go_arm_1][hh6_sname]: What date did you receive the 2nd dose? (dd-mm-yyyy)<br><br>Please provide an estimate if you cannot recall the date	________________________

Thank you for letting us know that someone in your household has been ill. We hope they feel better soon. Please always follow NHS and Public Health advice when someone is ill. We will always have a link to the latest COVID-19 advice on the Virus Watch Website.

If anyone has new symptoms to report, the following survey will ask about any illness and related health care as well as asking about isolation, time off work, measures to help stop infections spreading and activities that household members have done in the last week.

The survey usually takes about 10 minutes to complete for each member of the household who has been ill.

Thank you for completing the survey - we will be in touch again next week.

For peer review only

Wkhh1

Symptoms - [go_arm_1][hh1_fname] [go_arm_1][hh1_sname]

The following sections are about the symptoms, use of treatments and access of medical services in the past week (the Monday-Sunday before you received the email with this survey link).

[go_arm_1][hh1_fname]: What parts of the body did your symptoms affect?<br>Select all that apply	☐ General symptoms (fevers, general muscle aches, headache, joint pain,extreme tiredness, trouble with daily activities around the house)<br>☐ Respiratory Symptoms (e.g. cough, shortness of breath, earache, sore throat, runny nose, blocked nose, sneezing, wheeze, loss or altered senses of smell or taste)<br>☐ Eyes (e.g. eye redness, eye pain, sticky eye, deterioration of eyesight)<br>☐ Rash<br>☐ Digestive symptoms (e.g. diarrhoea or loose stools, vomiting, nausea, abdominal pain)
[go_arm_1][hh1_fname] [go_arm_1][hh1_sname]: Which days did you have symptoms?<br>Please check all days that you had any of the above symptoms.	☐ Monday<br>☐ Tuesday<br>☐ Wednesday<br>☐ Thursday<br>☐ Friday<br>☐ Saturday<br>☐ Sunday

For peer review only

General symptoms - [go_arm_1][hh1_fname] [go_arm_1][hh1_sname]

[go_arm_1][hh1_fname] [go_arm_1][hh1_sname]: Which of these general symptoms did you have?
Select all that apply

- ☐ Fever
- ☐ Feeling Feverish
- ☐ Chills and shakes
- ☐ Night Sweats
- ☐ Muscle Aches
- ☐ Bone or Joint Aches
- ☐ Loss of Appetite
- ☐ Headache
- ☐ Lack of concentration
- ☐ Lighheaded or dizzy
- ☐ Not sleeping
- ☐ Fatigue / Feeling unusually tired
- ☐ Difficulty with daily activities around the house
- ☐ Needed extra time in bed
- ☐ Could not get out of bed
- ☐ Confusion, disorientation, or hallucinations (altered mental state)
- ☐ None of these symptoms

Please identify how severe your symptoms were

Fever

	Less than 37.8 C (100.0 F)	37.8-38.9 C (100-102 F)	39-39.9 C (102-103.9 F)	40 C (104 F) or more	Did not take temperature Don't remember
Monday	○	○	○	○	○
Tuesday	○	○	○	○	○
Wednesday	○	○	○	○	○
Thursday	○	○	○	○	○
Friday	○	○	○	○	○
Saturday	○	○	○	○	○
Sunday	○	○	○	○	○

Feeling Feverish

	None	Mild	Moderate	Severe
Monday	○	○	○	○
Tuesday	○	○	○	○
Wednesday	○	○	○	○
Thursday	○	○	○	○
Friday	○	○	○	○
Saturday	○	○	○	○
Sunday	○	○	○	○

Chills and Shakes

For peer review only

	None	Mild	Moderate	Severe
Monday	◯	◯	◯	◯
Tuesday	◯	◯	◯	◯
Wednesday	◯	◯	◯	◯
Thursday	◯	◯	◯	◯
Friday	◯	◯	◯	◯
Saturday	◯	◯	◯	◯
Sunday	◯	◯	◯	◯

Night Sweats

	None	Mild	Moderate	Severe
Monday	◯	◯	◯	◯
Tuesday	◯	◯	◯	◯
Wednesday	◯	◯	◯	◯
Thursday	◯	◯	◯	◯
Friday	◯	◯	◯	◯
Saturday	◯	◯	◯	◯
Sunday	◯	◯	◯	◯

Muscle Ache

	None	Mild	Moderate	Severe
Monday	◯	◯	◯	◯
Tuesday	◯	◯	◯	◯
Wednesday	◯	◯	◯	◯
Thursday	◯	◯	◯	◯
Friday	◯	◯	◯	◯
Saturday	◯	◯	◯	◯
Sunday	◯	◯	◯	◯

Bone or Joint ache

	None	Mild	Moderate	Severe
Monday	◯	◯	◯	◯
Tuesday	◯	◯	◯	◯
Wednesday	◯	◯	◯	◯
Thursday	◯	◯	◯	◯
Friday	◯	◯	◯	◯
Saturday	◯	◯	◯	◯
Sunday	◯	◯	◯	◯

Loss of Appetite

For peer review only

	None	Mild	Moderate	Severe
Monday	◯	◯	◯	◯
Tuesday	◯	◯	◯	◯
Wednesday	◯	◯	◯	◯
Thursday	◯	◯	◯	◯
Friday	◯	◯	◯	◯
Saturday	◯	◯	◯	◯
Sunday	◯	◯	◯	◯

Headache

	None	Mild	Moderate	Severe
Monday	◯	◯	◯	◯
Tuesday	◯	◯	◯	◯
Wednesday	◯	◯	◯	◯
Thursday	◯	◯	◯	◯
Friday	◯	◯	◯	◯
Saturday	◯	◯	◯	◯
Sunday	◯	◯	◯	◯

Confusion, disorientation, or hallucinations (altered mental state)

	None	Mild	Moderate	Severe
Monday	◯	◯	◯	◯
Tuesday	◯	◯	◯	◯
Wednesday	◯	◯	◯	◯
Thursday	◯	◯	◯	◯
Friday	◯	◯	◯	◯
Saturday	◯	◯	◯	◯
Sunday	◯	◯	◯	◯

Lack of Concentration

	None	Mild	Moderate	Severe
Monday	◯	◯	◯	◯
Tuesday	◯	◯	◯	◯
Wednesday	◯	◯	◯	◯
Thursday	◯	◯	◯	◯
Friday	◯	◯	◯	◯
Saturday	◯	◯	◯	◯
Sunday	◯	◯	◯	◯

Lightheaded or Dizzy

For peer review only

	None	Mild	Moderate	Severe
Monday	◯	◯	◯	◯
Tuesday	◯	◯	◯	◯
Wednesday	◯	◯	◯	◯
Thursday	◯	◯	◯	◯
Friday	◯	◯	◯	◯
Saturday	◯	◯	◯	◯
Sunday	◯	◯	◯	◯

Not Sleeping

	None	Mild	Moderate	Severe
Monday	◯	◯	◯	◯
Tuesday	◯	◯	◯	◯
Wednesday	◯	◯	◯	◯
Thursday	◯	◯	◯	◯
Friday	◯	◯	◯	◯
Saturday	◯	◯	◯	◯
Sunday	◯	◯	◯	◯

Fatigue/Feeling Unusually Tired

	None	Mild	Moderate	Severe
Monday	◯	◯	◯	◯
Tuesday	◯	◯	◯	◯
Wednesday	◯	◯	◯	◯
Thursday	◯	◯	◯	◯
Friday	◯	◯	◯	◯
Saturday	◯	◯	◯	◯
Sunday	◯	◯	◯	◯

Difficulties with Daily Activities

	None	Mild	Moderate	Severe
Monday	◯	◯	◯	◯
Tuesday	◯	◯	◯	◯
Wednesday	◯	◯	◯	◯
Thursday	◯	◯	◯	◯
Friday	◯	◯	◯	◯
Saturday	◯	◯	◯	◯
Sunday	◯	◯	◯	◯

Needed extra time in bed

For peer review only

	None	Mild	Moderate	Severe
Monday	◯	◯	◯	◯
Tuesday	◯	◯	◯	◯
Wednesday	◯	◯	◯	◯
Thursday	◯	◯	◯	◯
Friday	◯	◯	◯	◯
Saturday	◯	◯	◯	◯
Sunday	◯	◯	◯	◯

Could not get out of bed

	None	Mild	Moderate	Severe
Monday	◯	◯	◯	◯
Tuesday	◯	◯	◯	◯
Wednesday	◯	◯	◯	◯
Thursday	◯	◯	◯	◯
Friday	◯	◯	◯	◯
Saturday	◯	◯	◯	◯
Sunday	◯	◯	◯	◯

For peer review only

Respiratory Symptoms - [go_arm_1][hh1_fname] [go_arm_1][hh1_sname]

[go_arm_1][hh1_fname] [go_arm_1][hh1_sname]: Which Respiratory symptoms did you have?
Select all that apply

- ☐ Runny Nose
- ☐ Blocked Nose
- ☐ Sinus pain / congestion
- ☐ Dry cough
- ☐ Coughing up Green Phlegm
- ☐ Coughing up White Phlegm
- ☐ Loss or change to sense of smell
- ☐ Loss or change to sense of taste
- ☐ Sneezing
- ☐ Sore Throat
- ☐ Swollen tonsils
- ☐ Swollen glands (enlarged lymph nodes)
- ☐ Ear pain or change in hearing
- ☐ Fluid leaking from ear
- ☐ Shortness of breath difficulty breathing
- ☐ Wheezing
- ☐ Chest pain (not changed by breathing or moving)
- ☐ Chest pain when breathing in
- ☐ None of these symptoms

Please identify how severe your symptoms were

Runny Nose

	None	Mild	Moderate	Severe
Monday	○	○	○	○
Tuesday	○	○	○	○
Wednesday	○	○	○	○
Thursday	○	○	○	○
Friday	○	○	○	○
Saturday	○	○	○	○
Sunday	○	○	○	○

For peer review only

Blocked Nose

	None	Mild	Moderate	Severe
Monday	○	○	○	○
Tuesday	○	○	○	○
Wednesday	○	○	○	○
Thursday	○	○	○	○
Friday	○	○	○	○
Saturday	○	○	○	○
Sunday	○	○	○	○

Sinus Pain/Congestion

	None	Mild	Moderate	Severe
Monday	○	○	○	○

	None	Mild	Moderate	Severe
Tuesday	○	○	○	○
Wednesday	○	○	○	○
Thursday	○	○	○	○
Friday	○	○	○	○
Saturday	○	○	○	○
Sunday	○	○	○	○

Dry cough

	None	Mild	Moderate	Severe
Monday	○	○	○	○
Tuesday	○	○	○	○
Wednesday	○	○	○	○
Thursday	○	○	○	○
Friday	○	○	○	○
Saturday	○	○	○	○
Sunday	○	○	○	○

Coughing up Green Phlegm

	None	Mild	Moderate	Severe
Monday	○	○	○	○
Tuesday	○	○	○	○
Wednesday	○	○	○	○
Thursday	○	○	○	○
Friday	○	○	○	○
Saturday	○	○	○	○
Sunday	○	○	○	○

Coughing up White Phlegm

	None	Mild	Moderate	Severe
Monday	○	○	○	○
Tuesday	○	○	○	○
Wednesday	○	○	○	○
Thursday	○	○	○	○
Friday	○	○	○	○
Saturday	○	○	○	○
Sunday	○	○	○	○

Loss or change to sense of smell

For peer review only

	None	Mild	Moderate	Severe
Monday	◯	◯	◯	◯
Tuesday	◯	◯	◯	◯
Wednesday	◯	◯	◯	◯
Thursday	◯	◯	◯	◯
Friday	◯	◯	◯	◯
Saturday	◯	◯	◯	◯
Sunday	◯	◯	◯	◯

Loss or change to sense of taste

	None	Mild	Moderate	Severe
Monday	◯	◯	◯	◯
Tuesday	◯	◯	◯	◯
Wednesday	◯	◯	◯	◯
Thursday	◯	◯	◯	◯
Friday	◯	◯	◯	◯
Saturday	◯	◯	◯	◯
Sunday	◯	◯	◯	◯

Sneezing

	None	Mild	Moderate	Severe
Monday	◯	◯	◯	◯
Tuesday	◯	◯	◯	◯
Wednesday	◯	◯	◯	◯
Thursday	◯	◯	◯	◯
Friday	◯	◯	◯	◯
Saturday	◯	◯	◯	◯
Sunday	◯	◯	◯	◯

Sore Throat

	None	Mild	Moderate	Severe
Monday	◯	◯	◯	◯
Tuesday	◯	◯	◯	◯
Wednesday	◯	◯	◯	◯
Thursday	◯	◯	◯	◯
Friday	◯	◯	◯	◯
Saturday	◯	◯	◯	◯
Sunday	◯	◯	◯	◯

Swollen Tonsils

For peer review only

	None	Mild	Moderate	Severe
Monday	◯	◯	◯	◯
Tuesday	◯	◯	◯	◯
Wednesday	◯	◯	◯	◯
Thursday	◯	◯	◯	◯
Friday	◯	◯	◯	◯
Saturday	◯	◯	◯	◯
Sunday	◯	◯	◯	◯

Swollen Glands (enlarged lymph nodes)

	None	Mild	Moderate	Severe
Monday	◯	◯	◯	◯
Tuesday	◯	◯	◯	◯
Wednesday	◯	◯	◯	◯
Thursday	◯	◯	◯	◯
Friday	◯	◯	◯	◯
Saturday	◯	◯	◯	◯
Sunday	◯	◯	◯	◯

Ear pain or change in hearing

	None	Mild	Moderate	Severe
Monday	◯	◯	◯	◯
Tuesday	◯	◯	◯	◯
Wednesday	◯	◯	◯	◯
Thursday	◯	◯	◯	◯
Friday	◯	◯	◯	◯
Saturday	◯	◯	◯	◯
Sunday	◯	◯	◯	◯

Fluid leaking from ear

	None	Mild	Moderate	Severe
Monday	◯	◯	◯	◯
Tuesday	◯	◯	◯	◯
Wednesday	◯	◯	◯	◯
Thursday	◯	◯	◯	◯
Friday	◯	◯	◯	◯
Saturday	◯	◯	◯	◯
Sunday	◯	◯	◯	◯

Shortness of Breath/difficulty breathing

For peer review only

	None	Mild	Moderate	Severe
Monday	○	○	○	○
Tuesday	○	○	○	○
Wednesday	○	○	○	○
Thursday	○	○	○	○
Friday	○	○	○	○
Saturday	○	○	○	○
Sunday	○	○	○	○

Wheezing

	None	Mild	Moderate	Severe
Monday	○	○	○	○
Tuesday	○	○	○	○
Wednesday	○	○	○	○
Thursday	○	○	○	○
Friday	○	○	○	○
Saturday	○	○	○	○
Sunday	○	○	○	○

Chest pain (not changed by breathing or moving)

	None	Mild	Moderate	Severe
Monday	○	○	○	○
Tuesday	○	○	○	○
Wednesday	○	○	○	○
Thursday	○	○	○	○
Friday	○	○	○	○
Saturday	○	○	○	○
Sunday	○	○	○	○

Chest pain when breathing in

	None	Mild	Moderate	Severe
Monday	○	○	○	○
Tuesday	○	○	○	○
Wednesday	○	○	○	○
Thursday	○	○	○	○
Friday	○	○	○	○
Saturday	○	○	○	○
Sunday	○	○	○	○

For peer review only

Other Symptoms - [go_arm_1][hh1_fname] [go_arm_1][hh1_sname]

[go_arm_1][hh1_fname] [go_arm_1][hh1_sname]: Which eye related symptoms did you have this week?
Select all that apply

- ☐ Eye redness
- ☐ Sticky Eye
- ☐ Eye pain
- ☐ Deterioration of eyesight
- ☐ None of these symptoms

[go_arm_1][hh1_fname] [go_arm_1][hh1_sname]: Where did the rash affect you?

- ☐ Rash (all over)
- ☐ Rash (local)
- ☐ None of these symptoms

Where on the body did the rash affect you?

____

[go_arm_1][hh1_fname] [go_arm_1][hh1_sname]: Which gastrointestinal symptoms did you have?
Select all that apply

- ☐ Diarrhoea (even mild)
- ☐ Vomiting (being sick)
- ☐ Nausea (feeling sick)
- ☐ Abdominal pain (not including menstrual pain)
- ☐ None of these symptoms

Please identify how severe your symptoms were

Red Eye(s)

	None	Mild	Moderate	Severe
Monday	○	○	○	○
Tuesday	○	○	○	○
Wednesday	○	○	○	○
Thursday	○	○	○	○
Friday	○	○	○	○
Saturday	○	○	○	○
Sunday	○	○	○	○

Sticky Eye(s)

	None	Mild	Moderate	Severe
Monday	○	○	○	○
Tuesday	○	○	○	○
Wednesday	○	○	○	○
Thursday	○	○	○	○
Friday	○	○	○	○
Saturday	○	○	○	○
Sunday	○	○	○	○

Eye Pain

For peer review only

	None	Mild	Moderate	Severe
Monday	○	○	○	○
Tuesday	○	○	○	○
Wednesday	○	○	○	○
Thursday	○	○	○	○
Friday	○	○	○	○
Saturday	○	○	○	○
Sunday	○	○	○	○

Deterioration of eyesight

	None	Mild	Moderate	Severe
Monday	○	○	○	○
Tuesday	○	○	○	○
Wednesday	○	○	○	○
Thursday	○	○	○	○
Friday	○	○	○	○
Saturday	○	○	○	○
Sunday	○	○	○	○

Rash - All Over

	None	Mild	Moderate	Severe
Monday	○	○	○	○
Tuesday	○	○	○	○
Wednesday	○	○	○	○
Thursday	○	○	○	○
Friday	○	○	○	○
Saturday	○	○	○	○
Sunday	○	○	○	○

Rash - Local

	None	Mild	Moderate	Severe
Monday	○	○	○	○
Tuesday	○	○	○	○
Wednesday	○	○	○	○
Thursday	○	○	○	○
Friday	○	○	○	○
Saturday	○	○	○	○
Sunday	○	○	○	○

Diarrhoea (even mild)

For peer review only

	None	Mild	Moderate	Severe
Monday	◯	◯	◯	◯
Tuesday	◯	◯	◯	◯
Wednesday	◯	◯	◯	◯
Thursday	◯	◯	◯	◯
Friday	◯	◯	◯	◯
Saturday	◯	◯	◯	◯
Sunday	◯	◯	◯	◯

Vomiting (being sick)

	None	Mild	Moderate	Severe
Monday	◯	◯	◯	◯
Tuesday	◯	◯	◯	◯
Wednesday	◯	◯	◯	◯
Thursday	◯	◯	◯	◯
Friday	◯	◯	◯	◯
Saturday	◯	◯	◯	◯
Sunday	◯	◯	◯	◯

Nausea (feeling sick)

	None	Mild	Moderate	Severe
Monday	◯	◯	◯	◯
Tuesday	◯	◯	◯	◯
Wednesday	◯	◯	◯	◯
Thursday	◯	◯	◯	◯
Friday	◯	◯	◯	◯
Saturday	◯	◯	◯	◯
Sunday	◯	◯	◯	◯

Abdominal pain (not including menstrual pain)

	None	Mild	Moderate	Severe
Monday	◯	◯	◯	◯
Tuesday	◯	◯	◯	◯
Wednesday	◯	◯	◯	◯
Thursday	◯	◯	◯	◯
Friday	◯	◯	◯	◯
Saturday	◯	◯	◯	◯
Sunday	◯	◯	◯	◯

For peer review only

For peer review only - http://bmjopen.bmj.com/site/about/guidelines.xhtml

COVID-19 testing for [go_arm_1][hh1_fname] [go_arm_1][hh1_sname]

[go_arm_1][hh1_fname] [go_arm_1][hh1_sname]: Have you sought COVID-19 testing for this illness via the NHS, government or your employer?	◯ Yes<br>◯ No
[go_arm_1][hh1_fname] [go_arm_1][hh1_sname]: You said you tried to get tested for COVID-19 via the NHS, government or your employer for this illness. Did you get tested?	◯ Yes<br>◯ No
[go_arm_1][hh1_fname] [go_arm_1][hh1_sname]: Why did you not get an NHS test for COVID-19?	◯ I was advised the test was not needed<br>◯ I am waiting for an NHS test kit to arrive by post<br>◯ I could not get to a testing centre<br>◯ I felt better so decided not to get tested<br>◯ Other - please specify
Other (please specify):	__________________________________
[go_arm_1][hh1_fname] [go_arm_1][hh1_sname]: How long was the gap between your symptoms starting and you having an NHS COVID-19 test?	◯ Same day<br>◯ Next day<br>◯ 2 days later<br>◯ 3 to 4 days later<br>◯ 5 to 7 days later<br>◯ More than a week
What was the result of that test?	◯ Positive for COVID-19<br>◯ Negative for COVID-19<br>◯ The result was unclear<br>◯ Still awaiting result
[go_arm_1][hh1_fname] [go_arm_1][hh1_sname]: How long was the gap between your symptoms starting and you getting the NHS test results?	◯ Same day<br>◯ Next day<br>◯ 2 days later<br>◯ 3 to 4 days later<br>◯ 5 to 7 days later<br>◯ More than a week

For peer review only

Tracing - [go_arm_1][hh1_fname] [go_arm_1][hh1_sname]

If you had an illness that may be COVID-19 you should follow national advice on reporting this illness and seeking care. We will always have a link to the latest advice on the Virus Watch website.

[go_arm_1][hh1_fname] [go_arm_1][hh1_sname]: Have you reported your illness to any organisation other than Virus Watch?<br>Select all that apply	☐ I did not report my illness to any other organisation<br>☐ Yes to the NHS Contact tracing app<br>☐ Yes to Google/Android contact tracing app<br>☐ Yes to the NHS Test and Trace service<br>☐ Yes to my employer<br>☐ Yes to my GP<br>☐ Yes to the NHS 111 online coronavirus service<br>☐ Yes to the general NHS 111 service<br>☐ Other (please specify)
Other (please specify)	__________________________________
[go_arm_1][hh1_fname] [go_arm_1][hh1_sname]: Why did you not report your illness?	☐ I did not think it was COVID-19<br>☐ I didn't feel ill enough to need help<br>☐ I didn't know where to report it<br>☐ I didn't know I was supposed to report it<br>☐ I didn't want to self isolate<br>☐ I didn't want my contacts to have to self isolate<br>☐ I didn't want others to know that I might have COVID-19
[go_arm_1][hh1_fname] [go_arm_1][hh1_sname]: From the time two days before your illness started to now, have you had direct or close contact with anyone OTHER THAN HOUSEHOLD MEMBERS?	☐ Non-Household Direct Contacts - these are people you had direct physical contact with or with whom you exchanged at least a few words within a 2 metre distance (e.g. a handshake, embracing, kissing, contact sports).<br>☐ Non-Household Close contacts - these are people who were within 2 metres of you for 15 minutes or more but who you did not speak to or touch.<br>☐ I did not have direct or close contact with anybody other than household members
How many people did you have DIRECT CONTACT with other than household members?<br>Please enter digits only, e.g. '5' and not 'five'	__________________________________
How many people did you have CLOSE CONTACT with other than household members?<br>Please enter digits only, e.g. '5' and not 'five'	__________________________________
[go_arm_1][hh1_fname] [go_arm_1][hh1_sname]: Has anyone contacted you to ask about who you have been in contact with prior to or during your illness(contact tracing)?<br>Please select all that apply	☐ No<br>☐ Yes - my GP<br>☐ Yes - the NHS Test and Trace System<br>☐ Yes - telephone advisory service<br>☐ Yes - through an online form<br>☐ Yes - through an app<br>☐ Yes - through my employer<br>☐ Yes - through my place of education<br>☐ Yes - the local public team<br>☐ Other - please specify
Other please specify:	__________________________________

For peer review only

[go_arm_1][hh1_fname] [go_arm_1][hh1_sname]: Was anyone other than a household member asked to self-isolate because of contact with you?	◯ Yes - and I know how many were asked to self isolate<br>◯ Yes - but I don't know how many were asked to self isolate<br>◯ Nobody was asked to self isolate<br>◯ I don't know if anyone was asked to self isolate
How many people other than household contacts were asked to self-isolate because of contact with you?<br>Please enter digits only, e.g. '5' and not 'five'	__________________________

For peer review only

Health Advice / Consultation - [go_arm_1][hh1_fname] [go_arm_1][hh1_sname]

[go_arm_1][hh1_fname] [go_arm_1][hh1_sname]: You should follow official national advice about what to do if you have symptoms of COVID-19. We will always include a link to the latest advice on the Virus Watch website.

[go_arm_1][hh1_fname] [go_arm_1][hh1_sname]: Did the illness lead you (or someone else on your behalf) to seek advice about your symptoms this week?
This includes advice from: NHS 111 PharmaciesNurses or doctorsThe internetFriends and family

◯ Yes
◯ No

Where was the advice or information sought from?

☐ NHS 111 COVID-19 website
☐ NHS 111 COVID-19 phone line
☐ COVID-19 testing centre
☐ Internet (e.g. WebMD , NHS choices)
☐ Pharmacist
☐ GP (by phone)
☐ GP (visit to practice)
☐ GP (online (including video))
☐ Walk-in centre
☐ A&E
☐ Hospital
☐ Friends or family
☐ Other (please specify)

Other (Please Specify): ______________________________

On which days was the medical advice or information sought from these sources Please check all that that apply on each day

	Monday	Tuesday	Wednesday	Thursday	Friday	Saturday	Sunday
Internet (e.g. WebMD , NHS choices)	☐	☐	☐	☐	☐	☐	☐
NHS 111 COVID-19 phone line	☐	☐	☐	☐	☐	☐	☐
NHS 111 COVID-19 website	☐	☐	☐	☐	☐	☐	☐
Pharmacist	☐	☐	☐	☐	☐	☐	☐
GP (by phone)	☐	☐	☐	☐	☐	☐	☐
GP (visit to practice)	☐	☐	☐	☐	☐	☐	☐
GP (online (including video))	☐	☐	☐	☐	☐	☐	☐
Walk-in centre	☐	☐	☐	☐	☐	☐	☐
COVID-19 testing centre	☐	☐	☐	☐	☐	☐	☐
A&E	☐	☐	☐	☐	☐	☐	☐
Hospital	☐	☐	☐	☐	☐	☐	☐

For peer review only

Medication - [go_arm_1][hhl_fname] [go_arm_1][hh1_sname]

[go_arm_1][hh1_fname] [go_arm_1][hh1_sname]: Did your symptoms lead to taking any medicines during the survey week?
This includes prescribed medicines, medicine brought at the chemist or shops, or vitamin supplements

◯ Yes
◯ No

On which days did you take the medicines:
Please select all that apply

	Mon	Tues	Wed	Thurs	Fri	Sat	Sun
Paracetamol	☐	☐	☐	☐	☐	☐	☐
Ibuprofen, nurofen, diclofenac, naproxen or other NSAID	☐	☐	☐	☐	☐	☐	☐
Aspirin	☐	☐	☐	☐	☐	☐	☐
Antibiotics	☐	☐	☐	☐	☐	☐	☐
Cold or flu remedies - over the counter	☐	☐	☐	☐	☐	☐	☐
Vitamin Supplements	☐	☐	☐	☐	☐	☐	☐
Other (please describe)	☐	☐	☐	☐	☐	☐	☐

Please specify: ____________________

For peer review only

Isolation and Infection Control - [go_arm_1][hh1_fname]

[go_arm_1][hh1_fname] [go_arm_1][hh1_sname]: During the past week, on which days did you:

	Mon	Tues	Wed	Thurs	Fri	Sat	Sun	None
Leave the house/flat or garden	☐	☐	☐	☐	☐	☐	☐	☐
Wear a facemask or face covering outside the home	☐	☐	☐	☐	☐	☐	☐	☐
Sleep in a room with no one else in it?	☐	☐	☐	☐	☐	☐	☐	☐
Wear a face mask or face covering at home?	☐	☐	☐	☐	☐	☐	☐	☐
Have a meal with other members of your household?	☐	☐	☐	☐	☐	☐	☐	☐
Watch television with other members of your household?	☐	☐	☐	☐	☐	☐	☐	☐

[go_arm_1][hh1_fname] [go_arm_1][hh1_sname]: During your illness, what best describes your approach to preventing spread of infection in the household?

- ○ I did not think my illness was COVID-19, so I did not take any special precautions
- ○ I thought it would spread whatever I did so did not take special precautions
- ○ I was worried about the illness spreading to others in the household so did what I could to stop this
- ○ None of the above

[go_arm_1][hh1_fname] [go_arm_1][hh1_sname]: Since your illness started, on which days did OTHER MEMBERS OF YOUR HOUSEHOLD:

	Mon	Tues	Wed	Thur	Fri	Sat	Sun	None
Wear a face mask or face covering when in the same room as you	☐	☐	☐	☐	☐	☐	☐	☐
Leave the house/flat or garden at all	☐	☐	☐	☐	☐	☐	☐	☐
Go to work	☐	☐	☐	☐	☐	☐	☐	☐
Wear a facemask or face covering outside the home	☐	☐	☐	☐	☐	☐	☐	☐

[go_arm_1][hh1_fname] [go_arm_1][hh1_sname]: Why did other household members leave the house?
Please select all that apply

- ☐ They did not think my symptoms were related to COVID-19
- ☐ They did not think there was any need for people without symptoms to stay at home
- ☐ They needed to go out to earn money
- ☐ They needed to go out to an important meeting
- ☐ Other reason

For peer review only

For peer review only - http://bmjopen.bmj.com/site/about/guidelines.xhtml

During your illness, how frequently have you washed your hands thoroughly and regularly with soap and water	◯ Not at all<br>◯ 1 or 2 times a day<br>◯ 3 or 4 times a day<br>◯ 5 or 6 times a day<br>◯ 7 or 8 times a day<br>◯ 9 or 10 times a day<br>◯ More than 10 times a day
During your illness, on average, how frequently have you (or someone else) disinfected surfaces you might touch?<br>Such as door knobs or hard surfaces	◯ Several times a day<br>◯ Daily<br>◯ Less than daily<br>◯ Never

[go_arm_1][hh1_fname] [go_arm_1][hh1_sname]: During your illness, how frequently have you:

	Not applicable	Almost always	Most of the time	Sometimes	Rarely	Never
Washed your hands after blowing your nose, sneezing or coughing	◯	◯	◯	◯	◯	◯
Used tissues when sneezing or coughing	◯	◯	◯	◯	◯	◯
Put tissues in the bin immediately after use	◯	◯	◯	◯	◯	◯

For peer review only

Thank You

Please click submit to continue

For peer review only
